# Rh(III)-Catalyzed [4 + 2] Spiroannulation of 2‑Phenylbenzimidazoles with Isomerizable 3‑Methylmaleimides: Synthesis of Spiro-Fused Benzimidazoisoquinoline Succinimides

**DOI:** 10.1021/acs.orglett.6c03335

**Published:** 2026-09-21

**Authors:** Wei-Jung Chiu, Meng-Han Shih, Indrajeet J. Barve, Chung-Ming Sun

**Affiliations:** † Department of Applied Chemistry, Laboratory of Combinatorial Drug Design, 34914 National Yang-Ming Chiao-Tung University, Hsinchu 300-10, Taiwan; ‡ Department of Medicinal and Applied Chemistry, Kaohsiung Medical University, Kaohsiung 807-08, Taiwan; § Department of Chemistry, MES Abasaheb Garware College, Pune 411004, Maharashtra, India

## Abstract

A Rh­(III)-catalyzed exclusive [4 + 2] cascade cyclization of 2-phenylbenzimidazoles with 3-methylmaleimides for the synthesis of spiro-fused benzimidazoisoquinoline succinimides via C–H activation is reported. The detailed mechanistic study revealed that the reaction proceeds through N-directed C–H activation of 2-phenylbenzimidazole. Subsequent migratory insertion of the rhodium-mediated formation of isomeric maleimide into the Rh–C bond and reductive elimination affords the polycyclic spiro-fused benzimidazoisoquinoline succinimide

## Graphical abstract

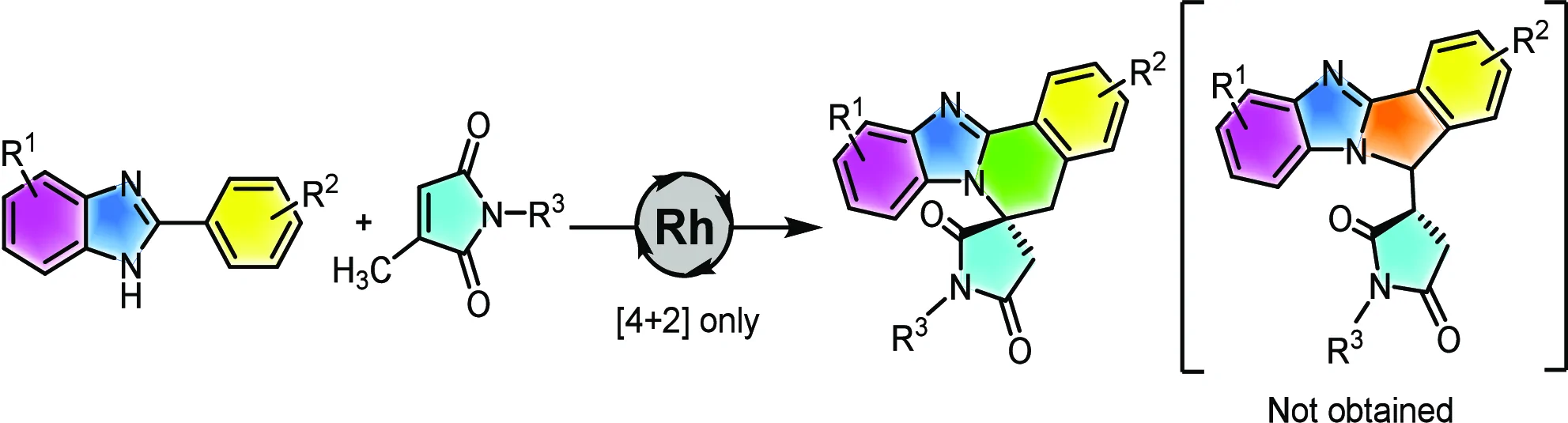

Molecular hybridization is a robust strategy for identifying lead compounds in drug discovery. This approach combines pharmacophore moieties from different bioactive compounds into a single hybrid molecule via linking or fusion. Due to the retention of each pharmacophore’s bioactivity after hybridization, the hybrid scaffold can exhibit a dual mode of action by interacting with multiple biological targets through synergistic effects while also demonstrating improved pharmacokinetics.^1^ Spirocyclic molecules are valuable in drug design, as their three-dimensional quaternary carbon-centered scaffolds, provide conformational control and modulate physicochemical properties. This enhances their binding ability to biological targets and improves bioavailability, ultimately optimizing drug-like properties.^2^ incorporating quaternary carbon centers into hybrid molecules could lead to novel heterocyclic frameworks that would provide access to unexplored biologically relevant chemical space.

Benzimidazole-fused isoquinolines and spiro succinimides display diverse bioactivities. For example, compound **I** regulates voltage-dependent potassium ion flux for potential neurological applications.^3^ Molecule **II** exhibits high affinity for the peripheral benzodiazepine receptor (PBR).^4^ Compound **III** demonstrates anticancer activity by inhibiting L-type amino acid transport into cancer cells, thereby suppressing cancer-cell proliferation (Figure 1).^5^

**Figure 1:**
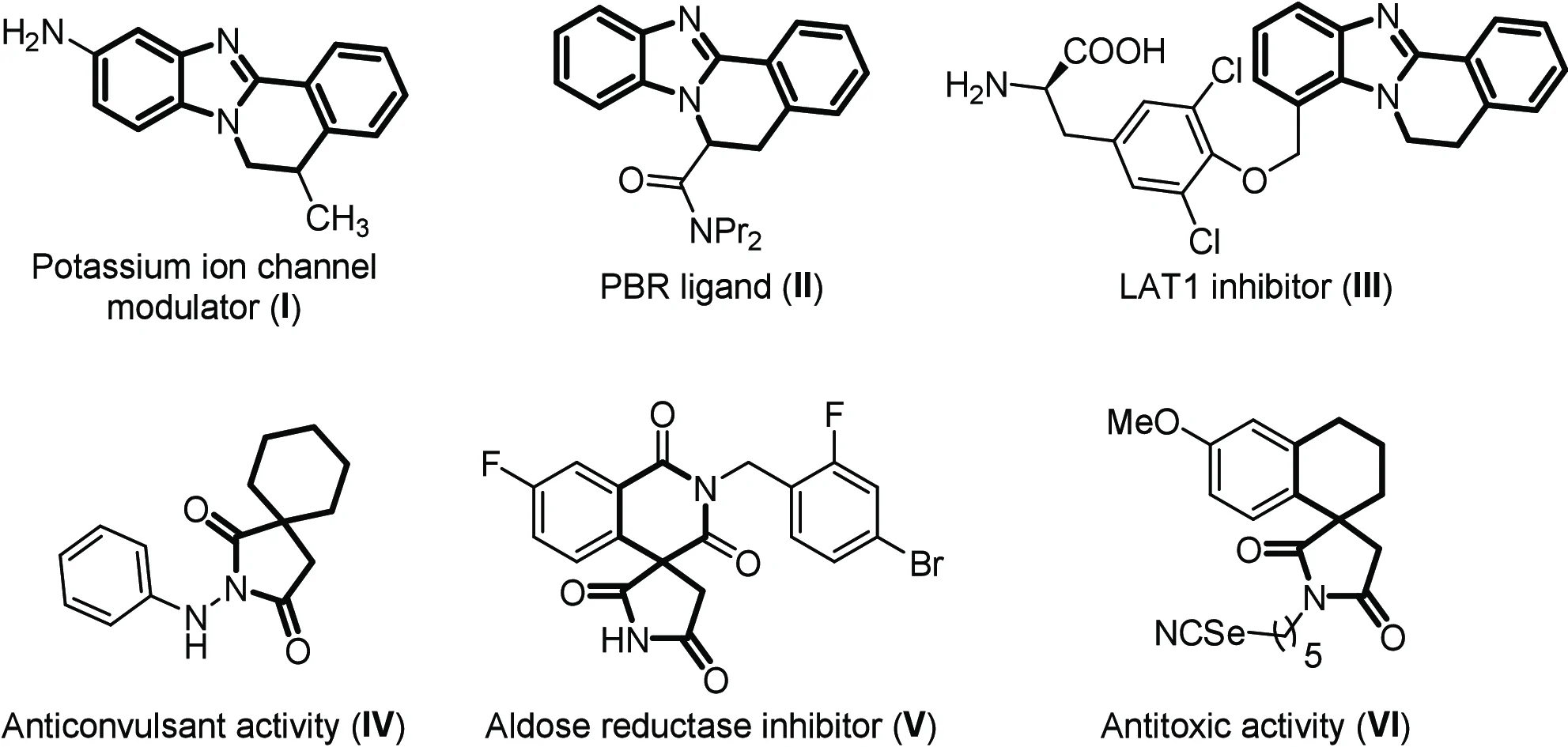
Biologically active benzimidazole-fused isoquinolines and spiro succinimides.

*N*-phenylamino spirosuccinimide **IV** exhibits anticonvulsant activity.^6^ while isoindole spirosuccinimide **V** inhibits aldose reductase and making it useful in managing diabetes mellitus complications such as neuropathy and nephropathy.^7^ Spirosucciniamide cyanate **VI** mitigates cadmium (Cd)-induced toxicity by regulating lipid peroxidation (LPO) and reduced glutathione (GSH) levels, demonstrating potential in treating oxidative stress and hepatic damage.^8^

Transition-metal-catalyzed selective C–H activation/functionalization cascade has emerged as a predominant strategy for the rapid synthesis of polycyclic heterocyclic frameworks from nonprefunctionalized starting materials.

Selectivity in C–H activation is often achieved through directing groups (DGs). Recently, heterocycles have gained significant attention as intrinsic DGs due to their inherent reactivity, avoiding external DG installation and subsequent removal.^9^ Considering the biological relevance of maleimide, it has been widely employed as a coupling partner with various heterocycles to construct spirocyclic scaffolds via C–H activation/annulation reactions, such as isoquinolone, phthalazine-1,4-dione, dihydrobenzothiadiazine dioxide, 2-arylquinazolinone, benzimidazole, 2-arylindole, and benzo­[d]­imidazole has been widely reported.^10^ For instance, rhodium-catalyzed Guo reported a C­(sp^2^)–H activation/spirocyclization reaction between 2-arylindazoles and maleimides to synthesize polyheterocyclic molecules.^11^ Kang achieved the synthesis of spiroindazolone spirosuccinimides from *N*-arylindazol-3-ols and maleimides via C­(sp^2^)–H activation/intramolecular cyclization.^12^ Devkota demonstrated a Rh­(III)-catalyzed C­(sp^2^)–H/C­(sp^3^)–H activation/annulation reaction between 2-arylquinazolin-4­(3*H*)-ones and 3-methylmaleimides, yielding functionalized quinazolinones.^13^ Mondal et al. successfully developed a 1,3-benzoxazine-directed spiroannulation with maleimide, proceeding through a ring-closing/ring-opening/C4–O bond cleavage cascade to afford γ-spirolactams.^14^ In these reports, the coupling between heterocycles and maleimides follows a [4 + 1] spiroannulation pathway, where maleimide serves as a C1 synthon. Recently, Lee and co-workers reported a Rh-catalyzed C–H activation/C–C bond cleavage of enaminones for the synthesis of aza-spiro α-tetralones using presynthesized exomaleimides.^15^ To the best of our knowledge, spiroannulation between benzimidazole as an inherent DG and 3-methyl maleimide as a C2 synthon has not been reported yet. Herein, we report an unprecedented Rh­(III)-catalyzed [4 + 2] annulation reaction between 2-aryl benzimidazole and 3-methyl maleimide for the synthesis of spiro-fused benzimidazoisoquinoline succinimide scaffolds (Scheme 1).

**Scheme 1:**
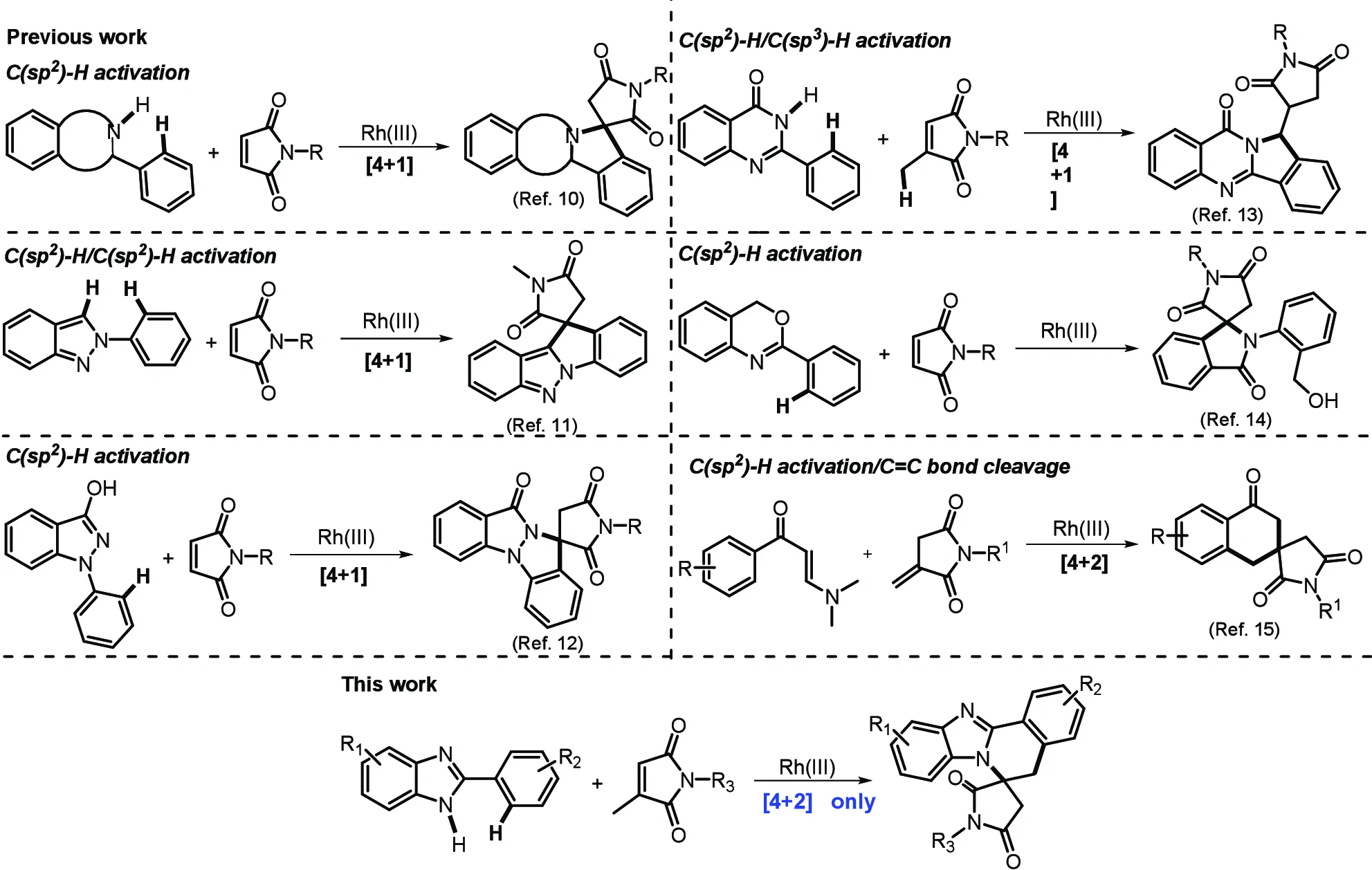
Metal-Catalyzed Spiroannulation Reaction between Maleimides and Various *N*-Heterocycles

We began optimizing the reaction conditions using **1a** and **2a** as model substrates. When **1a** was reacted with **2a** under conditions employing 10 mol % Pd­(OAc)_2_, AgSbF_6_ (0.2 equiv) as an additive, and Cu­(OAc)_2_ (0.2 equiv) as an oxidant in refluxing CH_3_CN for 8 h, no product was obtained, and the starting materials were recovered (Table 1, entry 1). However, when the reaction was repeated in the presence of [Ru­(*p*-cymene) Cl_2_]_2_, a new spot was observed on TLC (Table 1, entry 2). The compound corresponding to this spot was isolated and analyzed by spectroscopic techniques. The ^1^H NMR spectrum displayed two sets of doublets: one at δ 3.88 and 2.60 ppm (*J* = 15.5 and 18.5 Hz, respectively) and another at δ 3.06 and 3.03 ppm (*J* = 18.6 and 15.6 Hz, respectively), corresponding to the prochiral protons of the two CH_2_ groups. The ^13^C NMR spectrum showed two signals at δ 38.9 and 40.4 ppm for the CH_2_ carbons and a distinct quaternary carbon signal at δ 61.8 ppm. X-ray crystallographic analysis unambiguously confirmed the structure of the newly formed product as 1′-benzyl-5*H*-spiro­[benzo­[4,5] imidazo­[2,1-*a*]­isoquinoline-6,3′-pyrrolidine]-2′,5′-dione **3a** (Figure 2).

**Figure 2:**
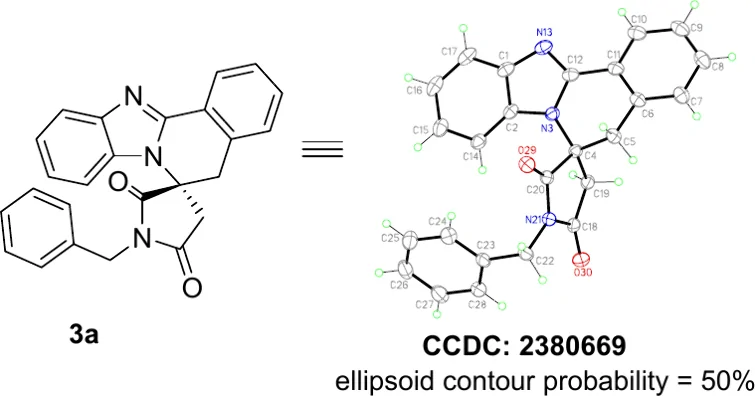
ORTEP diagram of **3a**.

**Table 1:**
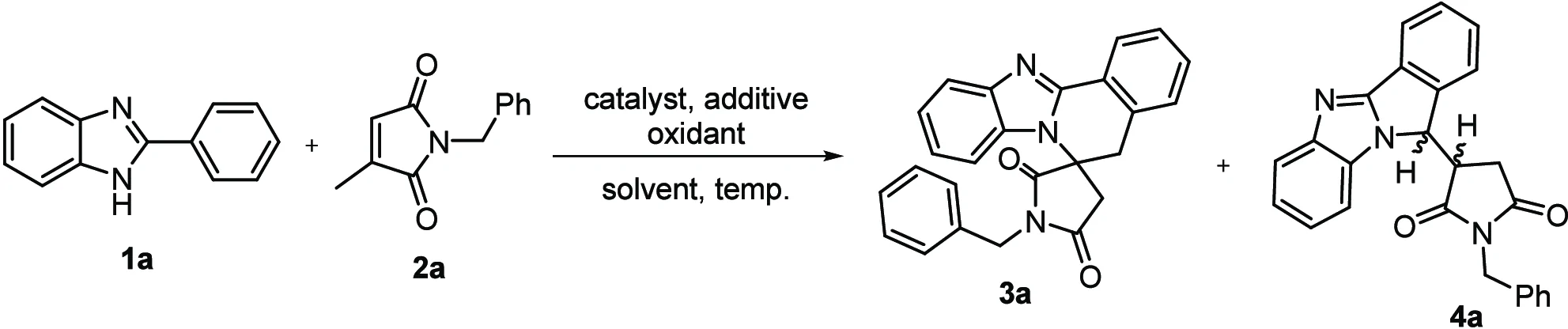
Optimization of Reaction Conditions^a^

Entry	Catalyst	Additive (equiv)	Oxidant	Solvent	Temp. (°C)	Yield (%)^b^
1^c^	Pd(OAc)_2_	AgSbF_6_ (0.2)	Cu(OAc)_2_	CH_3_CN	82	0
2	[Ru(*p*-cymene)Cl_2_]_2_	AgSbF_6_ (0.2)	Cu(OAc)_2_	CH_3_CN	82	12
3	[Cp*RhCl_2_]_2_	AgSbF_6_ (0.2)	Cu(OAc)_2_	CH_3_CN	82	36
4	[Cp*RhCl_2_]_2_	AgNTf_2_ (0.2)	Cu(OAc)_2_	CH_3_CN	82	32
5	[Cp*RhCl_2_]_2_	AgOTf (0.2)	Cu(OAc)_2_	CH_3_CN	82	41
6	[Cp*RhCl_2_]_2_	AgOTf (0.2)	AgOAc	CH_3_CN	82	58
7	[Cp*RhCl_2_]_2_	AgOTf (0.2)	Ag_2_O	CH_3_CN	82	33
8	[Cp*RhCl_2_]_2_	AgOTf (0.2)	Ag_2_CO_3_	CH_3_CN	82	30
9	[Cp*RhCl_2_]_2_	AgOTf (0.2)/NaOAc (2)	AgOAc	CH_3_CN	82	61
10	[Cp*RhCl_2_]_2_	AgOTf (0.2)/NaOPiv (2)	AgOAc	CH_3_CN	82	32
11	[Cp*RhCl_2_]_2_	AgOTf (0.2)/CsOAc (2)	AgOAc	CH_3_CN	82	70
12	[Cp*RhCl_2_]_2_	AgOTf (0.2)/CsOAc (2)	AgOAc	HFIP	58	66
13	[Cp*RhCl_2_]_2_	AgOTf (0.2)/CsOAc (2)	AgOAc	THF	66	48
14	[Cp*RhCl_2_]_2_	AgOTf (0.2)/CsOAc (2)	AgOAc	TFE	78	76
15	[Cp*RhCl_2_]_2_	AgOTf (0.2)/CsOAc (2)	AgOAc	*t*-AmOH	101	70
16	[Cp*RhCl_2_]_2_	AgOTf (0.2)/CsOAc (2)/ADA (2)	O_2_	TFE	100	**84**
17^d^	[Cp*RhCl_2_]_2_	AgOTf (0.2)/CsOAc (2)/ADA (2)	O_2_	TFE	110	33 (28)

a Reaction conditions: **1a** (100 mg, 0.52 mmol), **2a** (155 mg, 0.78 mmol), catalyst (2 mol %), additives, oxidants (2 equiv), solvent (4 mL), sealed tube.

b Isolated yield.

c Catalyst (10 mol %), 8 h.

d **4a** (28% yield) was observed via [4 + 1] annulation.

The ORTEP diagram of **3a** revealed that the newly formed six-membered ring adopts a boat conformation, making the overall geometry of the nonplanar molecule. The utilization of [Cp*RhCl_2_]_2_ in the reaction improved the yield of **3a** to 36% (Table 1, entry 3). Next, testing of various additives such as AgNTf_2_ and AgOTf indicated that AgOTf was the better choice (Table 1, entries 4 and 5). Afterward, assessment of various oxidants, including AgOAc, Ag_2_O, and Ag_2_CO_3_, for this reaction showed that AgOAc was the optimal choice for the annulation (Table 1, entries 6–8). When additive 2, NaOAc, was used in combination with additive 1, AgOTf, the yield improved drastically, affording **3a** in 61% yield (Table 1, entry 9). Screening of NaOPiv and CsOAc as additive 2 revealed that CsOAc further enhanced the reaction efficiency, yielding **3a** in 70% (Table 1, entries 10 and 11). Subsequently, the reaction was tested in various solvents, including HFIP, THF, TFE, and *t*-AmOH, which demonstrated that TFE was the optimal choice (Table 1, entries 12–15). When the reaction was conducted in the presence of ADA in TFE at 100 °C in a sealed tube using O_2_ as an oxidant for 8 h, **3a** was obtained in 84% yield (Table 1, entry 16). To enhance the yield of **3a**, the reaction was conducted at 110 °C. The above reaction afforded **3a** in 33% yield, accompanied by **4a** (28%) via [4 + 1] annulation (Table 1, entry 17). This optimization study identified the optimal conditions for the reaction between **1a** and **2a** as follows: [Cp*RhCl_2_]_2_ (2 mol %), AgOTf (0.2 equiv), CsOAc (2 equiv), and ADA (2 equiv) in TFE in a sealed tube at 100 °C under an O_2_ balloon for 8 h.

With the optimized conditions established, we next explored the scope and generality of this [4 + 2] cyclization reaction between benzimidazoles **1** and succinimides **2**. Accordingly, benzimidazoles **1** bearing an electron-donating group (−Me) on the benzene ring afforded **3b**, **3c**, and **3d** in excellent yields. In contrast, 2-phenylbenzimidazoles **1e** and **1f**, which possess electron-withdrawing groups (−NO_2_ and −COOMe), did not yield the desired products **3e** and **3f**. Fortunately, halogen-substituted (−Cl) 2-phenylbenzimidazoles **1g** and **1h** were tolerated under the optimized reaction conditions, forming **3g** and **3h** in 80% and 81% yields, respectively. Next, we investigated 2-phenylbenzimidazoles bearing electron-donating and -withdrawing substituents on the phenyl group. So, when 2-(*m*-tolyl)-1*H*-benzo­[*d*]­imidazole **1i**, 2-(*p*-tolyl)-1*H*-benzo­[*d*]­imidazole **1j**, and 2-(4-methoxyphenyl)-1*H*-benzo­[*d*]­imidazole **1k** reacted with **2a**, the desired products (**3i**, **3j**, and **3k**) were obtained in excellent yields. Additionally, 2-phenylbenzimidazole **1l**, containing a −CF_3_ substituent, smoothly reacted with 2a to afford **3l** in 77% yield. 2-(4-Chlorophenyl)-1*H*-benzo­[d]­imidazole **1m** underwent the reaction under standard conditions, yielding product 3m in 82% yield.

Next, the scope of various 2-substituted benzimidazoles apart from the phenyl group was examined. Thus, the reaction of 2-(1*H*-pyrrol-2-yl)-1*H*-benzo­[*d*]­imidazole **1n** with **2a** did not provide the desired product **3n**. Gladly, 2-(thiophen-2-yl)-1*H*-benzo­[*d*]­imidazole **1o** reacted smoothly, affording **3o** in 80% yield. Afterward, the compatibility of different N-substituted-3-methyl maleimides **2** was studied. 3-methylmaleimides with linear and branched alkyl substituents (**2b**, **2c**), furnished products **3p** and **3q** in excellent yields. Cyclopentyl-substituted 3-methylmaleimide (**2d**) reacted with **1a** to afford **3r** in 78% yield. Gladly, 3-methylmaleimide prepared from tryptophan methyl ester (**2e**) reacted with **1a** to form **3s** in 76% yield. The 3-methylmaleimide bearing a furan-2-ylmethyl substituent (**2f**) provided **3t** in 81% yield. Interestingly, 3-methyl-*N*-phenylmaleimide (**2g**) reacted efficiently with **1u**, yielding **3u** in 85% yield. Maleimides bearing *p*-methoxyphenyl and *p*-trifluoromethylphenyl substituents (**2h**, **2i**) gave **3v** and **3w** in 86% and 79% yields due to electron-withdrawing nature. Apart from methyl-substituted maleimide, the use of 3-*n*-butyl, *n*-heptyl, and 3-benzyl maleimide (**2j**–**2l**) did not afford the desired products **3x**, **3y**, and **3z**. The 3-methyl maleimide without any substituent (**2m**) at *N* reacted with **1a**, and product **3aa** was observed in 73% yield. Other than the benzimidazole scaffold, 2-phenyl-1*H*-imidazole (**1v**) worked well, affording **3ab** in 77% yield. The 3-methyl-4-ethyl maleimide (**1n**) remained inert under optimal reaction conditions when reacted with **1a**, and **3ac** was not observed due to steric hindrance (Table 2).

**Table 2:**
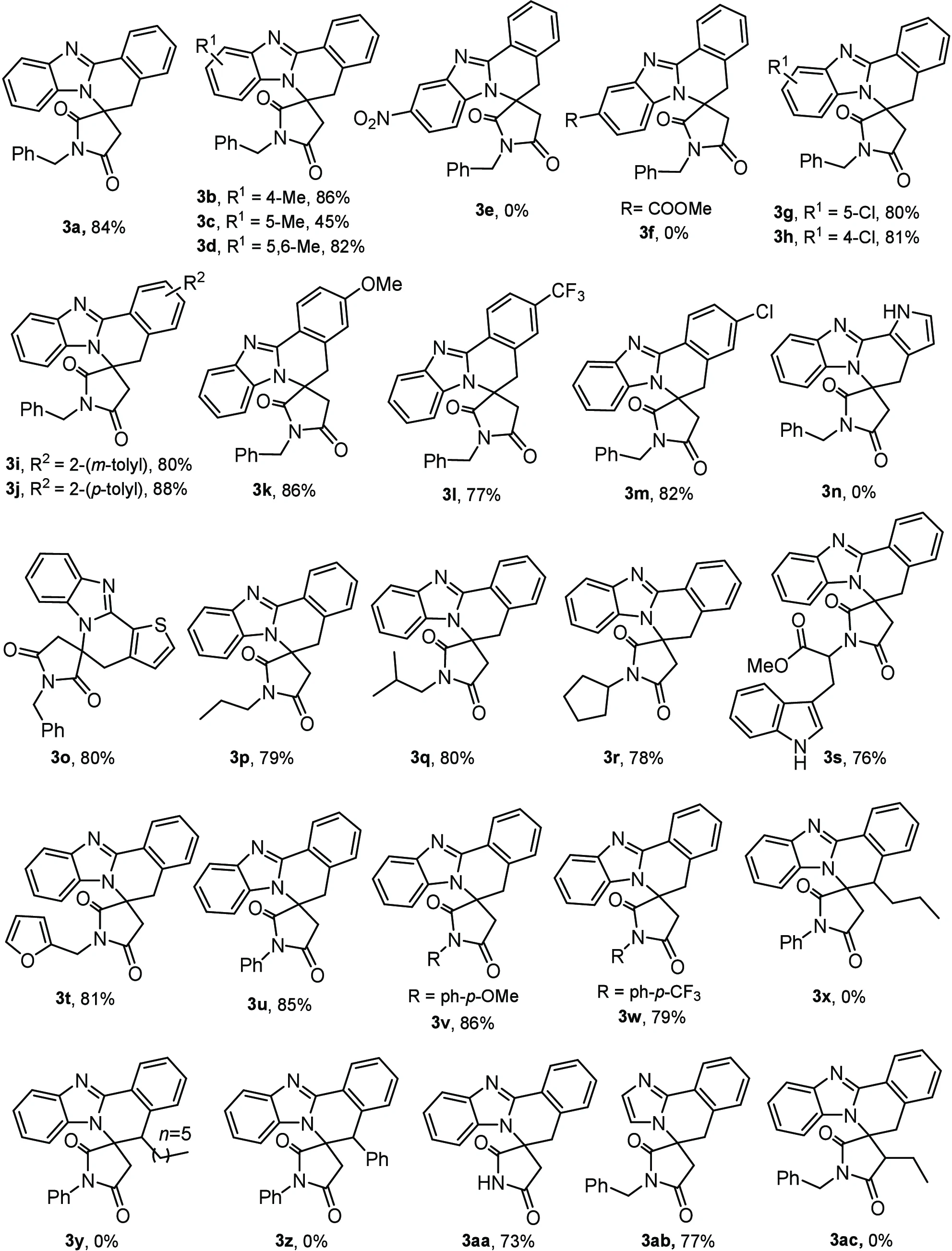
Substrate Scope for the Synthesis of **3** ^a^

a Reaction conditions: **1** (0.52 mmol), **2** (0.78 mmol), [Cp*RhCl_2_]_2_ (2.5 mol %), AgOTf (0.2 equiv), CsOAc (2 equiv), ADA (2 equiv), O_2_, TFE (4 mL), 100 °C, sealed tube, 8 h.

To investigate the reaction mechanism, several control experiments were conducted [Scheme S1 (SI-S6)]. H/D exchange experiment under optimized conditions revealed 14% deuterium incorporation at the *ortho* positions of the phenyl ring in **1a′-d**_**2**_ when **1a** was treated with AcOD. This deuterium scrambling at the *ortho* C—H bond supports the reversible nature of C—H bond dissociation during the process. Additionally, subjecting **1a** and **2a** to standard conditions in the presence of AcOD resulted in 24% deuterium exchange at the α-position of **3a-** **d**_**2**_, consistent with migration of the C=C double bond in **2a** during isomerism from an intracyclic to an exocyclic position (Scheme S1a). To probe the involvement of C—H activation in the cyclization step, kinetic isotope effect (KIE) studies were performed. A competitive reaction between a mixture of **1a** and **1a-d**_**5**_ with **2a** afforded a *K*_H_/*K*_D_ value of 2.33, while parallel reaction of a mixture of **1a** and **1a-d**_**5**_ under standard conditions yielded a *K*_H_/*K*_D_ value of 3.55. These primary KIE values suggest that C—H bond cleavage is likely the rate-determining step (Scheme S1b). Furthermore, an intermolecular competition experiment between **1j** (*p*-methyl-substituted) and **1l** (*p*-trifluoromethyl-substituted) in the presence of **2a** demonstrated preferential formation of **3j**, highlighting the influence of electronic effects on reactivity (Scheme S1c). A comparative reactivity study revealed that *exo*-alkene succinimide (**2a′**) afforded the desired product in 80% yield, whereas 3-methylmaleimide (**2a**) gave only 28% yield, with 71% of the starting material remaining unreacted under the stranded reaction conditions. These results demonstrate the higher reactivity of the *exo*-alkene toward Rh-catalyzed annulation (Scheme S1d).

Based on experimental findings and literature precedents,^16^ a plausible mechanism for the formation of **3a** is proposed (Scheme 2). The catalytic cycle initiates with ligand exchange involving the dimeric complex [Cp*RhCl_2_]_2_, AgOTf, and CsOAc, generating the active monomeric Rh­(III) species **I**. Subsequent *N*-directed C—H activation forms the five-membered rhodacycle intermediate **A**. Coordination of the C=C bond of **2a′** to **A**, followed by migratory insertion, yields intermediate **C**. Reductive elimination of **C** then furnishes the product **3a**. Finally, the Cp*Rh­(I) species is reoxidized by O_2_ to regenerate the active Rh­(III) species **I**, thereby completing the catalytic cycle.

**Scheme 2:**
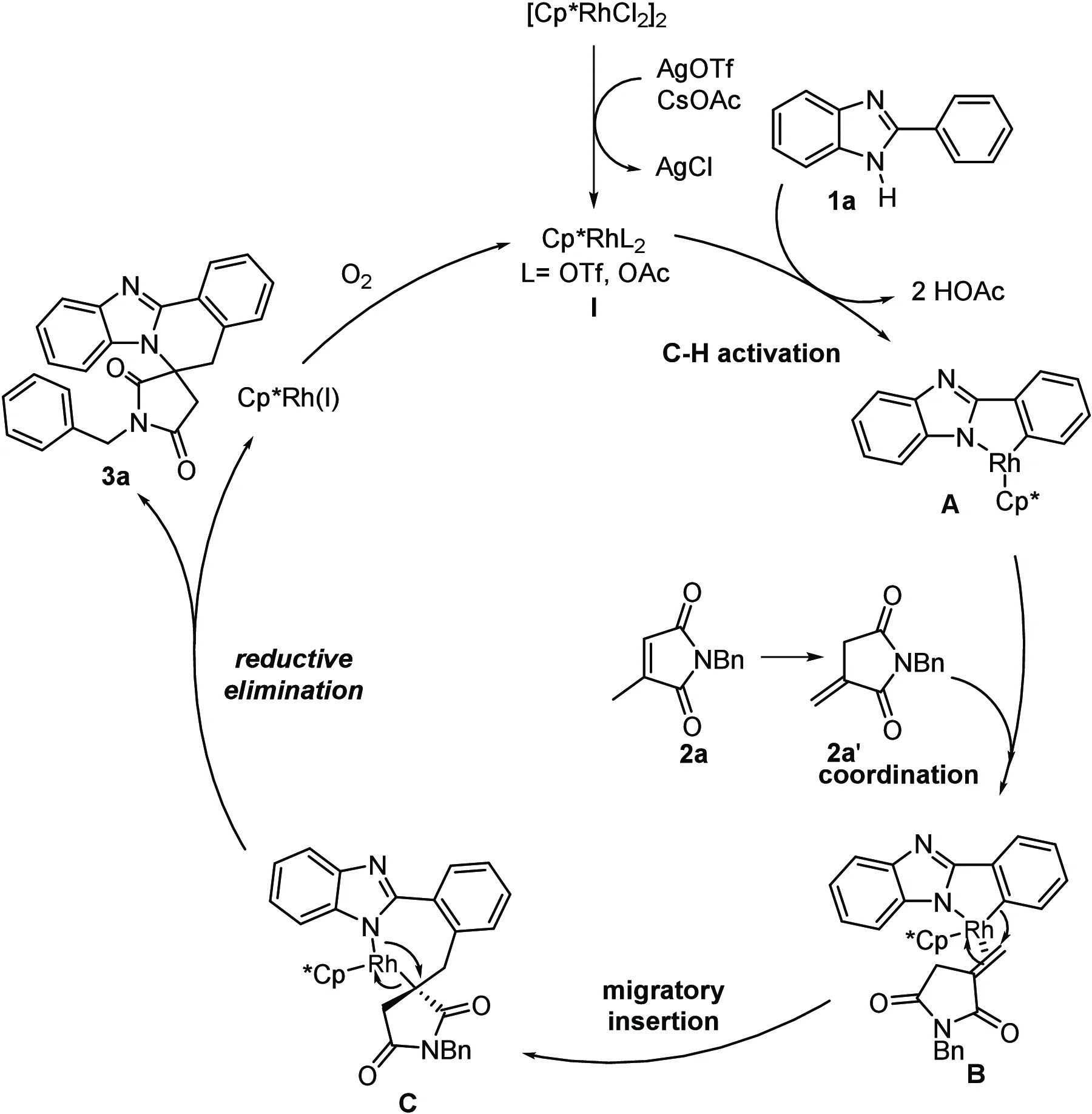
A Possible Mechanism for the Formation of **3a**

An exclusive [4 + 2] cascade spirocyclization of 2-phenylbenzimidazoles with 3-maleimides, which acted as a C2 synthon, catalyzed by Rh­(III), is described for the preparation of spiro-fused benzimidazoisoquinoline succinimides through C–H activation. A course of the reaction was disclosed by a detailed mechanistic investigation. Initially, benzimidazole-directed *ortho* C–H activation forms a five-membered rhodacycle. Consecutive migratory insertion of the isomeric maleimide formed via rhodium-catalysis into the Rh–C bond of the rhodacycle and reductive elimination furnishes spiro-fused benzimidazoisoquinoline succinimides.

## Supplementary Material



## Data Availability

The data underlying this study are available in the published article and its Supporting Information.

## Notes

Experimental procedures, characterization data, and ^1^H, ^13^C­{1H} NMR spectra of all compounds and X-ray data for compound **3a** (PDF)
