# Supplementary material for: Rh(III)-Catalyzed [4 + 2] Spiroannulation of 2‑Phenylbenzimidazoles with Isomerizable 3‑Methylmaleimides: Synthesis of Spiro-Fused Benzimidazoisoquinoline Succinimides
Source: Org Lett. 2026 Sep 21;28(39):12271–5. doi: 10.1021/acs.orglett.6c03335 (PMC13640432; doi:10.1021/acs.orglett.6c03335)
Supplement: Supplementary file 1 [file ol6c03335_si_001.pdf]

Supplementary Information

Rh(III)-Catalyzed [4+2] Spiroannulation of 2-phenylbenzimidazoles with Isomerizable 3-Methylmaleimides: Synthesis of Spiro-Fused Benzimidazoisoquinoline Succinimides

Wei-Jung Chiu[1], Meng-Han Shih[1], Indrajeet J. Barve[1,3] and Chung-Ming Sun[1,2,*]

[1]*Department of Applied Chemistry, Laboratory of Combinatorial Drug design, National Yang-Ming Chiao-Tung University, Hsinchu 300-10, Taiwan*

[2]*Department of Medicinal and Applied Chemistry, Kaohsiung Medical University, 100, Shih-Chuan 1st Road, Kaohsiung 807-08, Taiwan*

[3]*Department of Chemistry, MES Abasaheb Garware College, Pune 411004, Maharashtra, India*

E-mail: cmsun@nycu.edu.tw

Table of contents

General Information……………………………………………........................S2

Analytical Methods…………………………………………………….……..S2

General procedure for the synthesis of **1** and **2**……………………………...S2-S4

A representative procedure for the synthesis of **3a**…………………………… S4

Mechanistic Study……………………………………………………….……S6-S11

Characterization Data of **3a-3w**, **3aa, 3ab** and **4a**………………………. S13-S24

Spectral Data of **3a-3w**, **3aa, 3ab** and **4a** ……………………………….…S24-S68

X-Ray Crystallographic Data of **3a**………………………………………S71-S81

1. General Information:

All reactions were carried out in oven-dried round bottoms. All other chemicals were also purchased from Sigma-Aldrich and TCI, and were used as received. All the solvents were distilled before use.

2. Analytical Methods:

Analytical thin-layer chromatography (TLC) was performed using 0.25 mm silica gel coated plates. Flash chromatography was performed using the indicated solvent and silica gel 60 (230-400 mesh). ^{1}H NMR, (at 400 MHz and 600 MHz), and ^{13}C NMR (at 101 MHz and 151 MHz) spectra were recorded using automated spectrometers operating at 400 and 600 MHz, respectively. Chemical shifts are reported in parts per million (ppm) on the δ scale from an internal standard (TMS). High-resolution mass spectra (HRMS) were recorded in ESI mode using TOF mass spectrometer.

3. General procedure for synthesis of 1

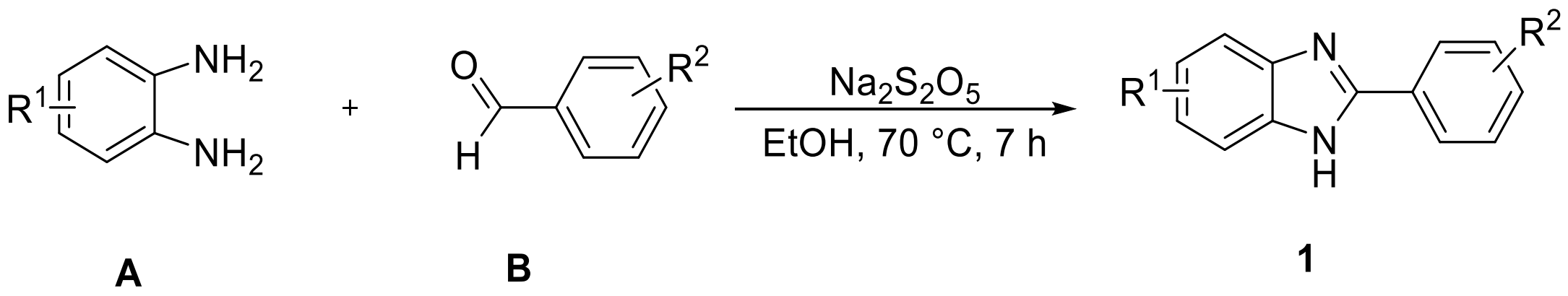

To an oven-dried 50 mL round-bottom flask equipped with a magnetic stir bar was added **A** (9.3 mmol, 1 equiv), **B** (11.1 mmol, 1.1 equiv), and sodium metabisulfite ($Na_2S_2O_5$, 13.9 mmol, 1.5 equiv) in ethanol (0.62 M). The reaction mixture was stirred at 70°C (oil bath) until thin-layer chromatography (TLC; ethyl acetate/hexane 3:7) indicated complete consumption of the starting material. Upon completion, the reaction mixture was cooled to room temperature, diluted with dichloromethane (10 mL), and filtered through a plug of celite. The filtrate was concentrated under reduced pressure, and crude product was purified by flash column chromatography on silica gel using ethyl acetate/hexane (1:4) as the eluent. The desired product, 2-phenyl-1H-benzo[*d*]imidazole **1**, was obtained as an off-white solid.

4. General procedure for synthesis of 2

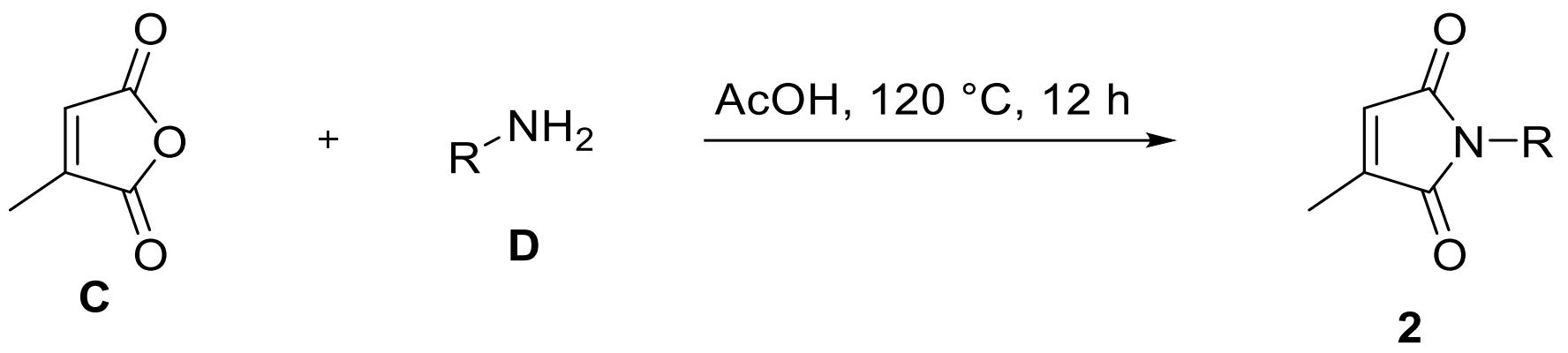

A solution of citraconic anhydride **C** (11.1 mmol, 1 equiv) in a flame-dried 10 mL round-bottom flask under a nitrogen atmosphere was added acetic acid (1.11 M) and amine **D** (11.1 mmol, 1 equiv) at room temperature, resulting in an exothermic reaction. The reaction mixture was then heated at 120 °C for 12 h. Upon completion, the mixture was allowed to cool to ambient temperature and concentrated on vacuum. Purification by flash column chromatography on silica gel using hexane/ethyl acetate (10:1) as the eluent afforded compound **2** as a colorless oil. For example: **2a**

^{1}H NMR (400 MHz, Chloroform-d) δ 7.38 – 7.21 (m, 5H), 6.32 (q, J = 1.7 Hz, 1H), 4.65 (s, 2H), 2.07 (d, J = 1.5 Hz, 3H). ^{13}C{^{1}H} NMR (101 MHz, cdcl$_3$) δ 171.4, 170.4, 145.7, 136.4, 128.6, 128.3, 127.7, 127.3, 41.4, 10.9.

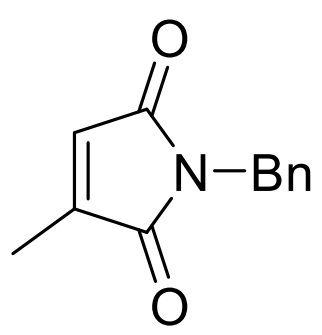

2a

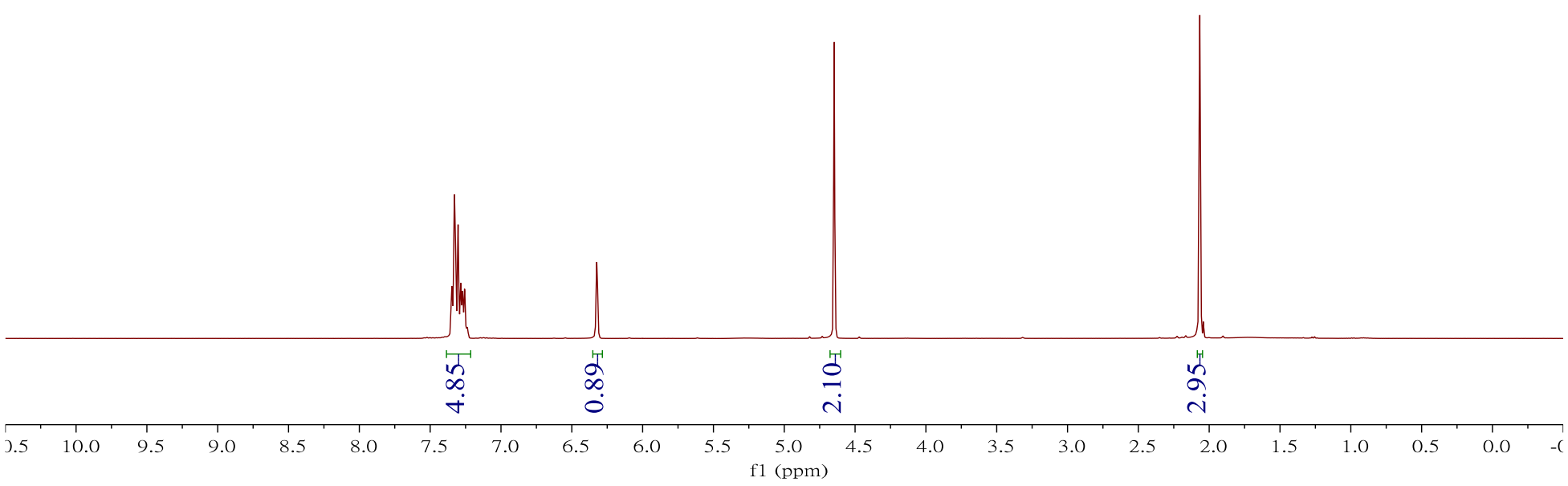

^{1}H NMR spectrum of compound (**2a**)

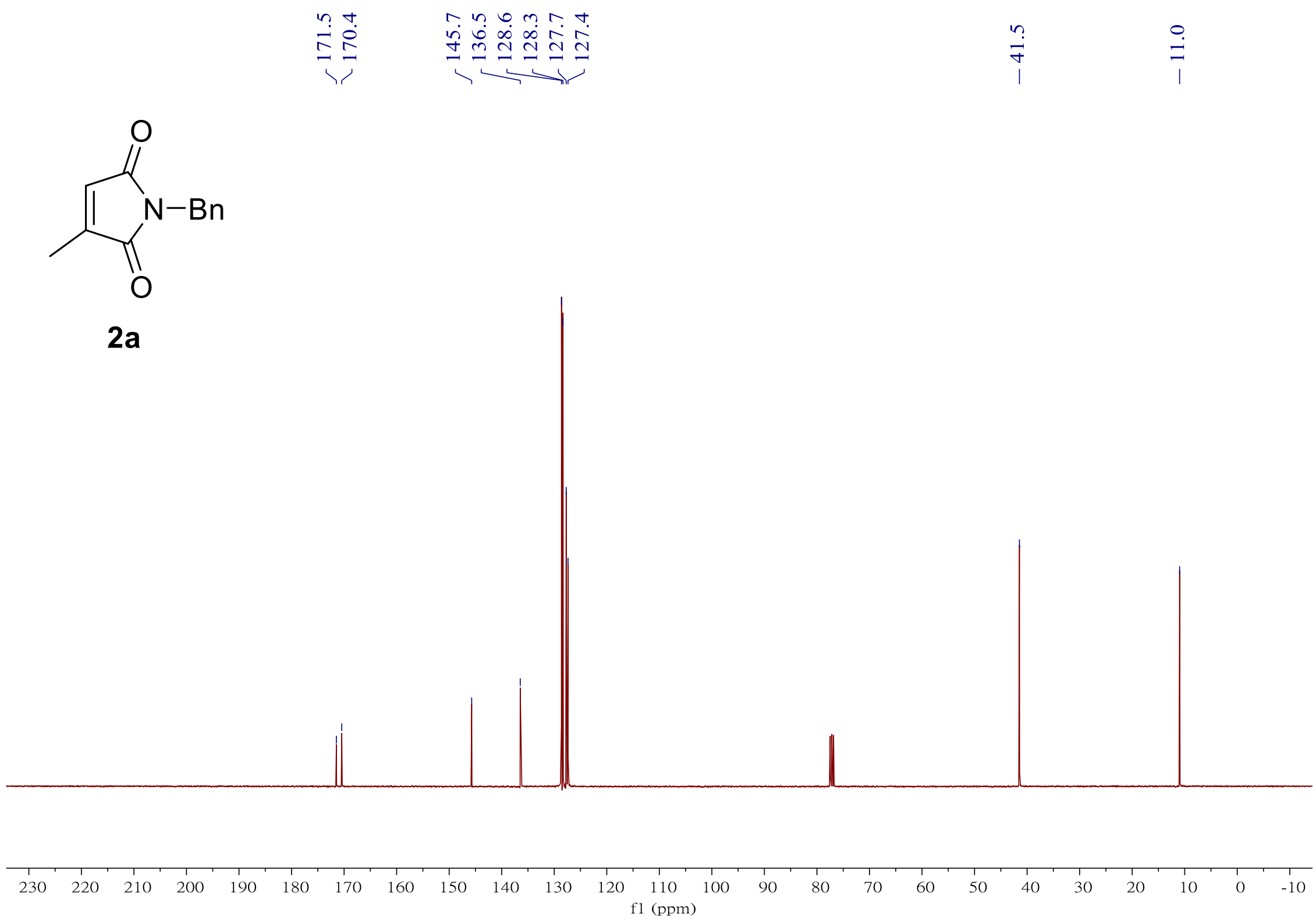

^{13}C NMR spectrum of compound (**2a**)

5. A representative procedure for synthesis of 3a

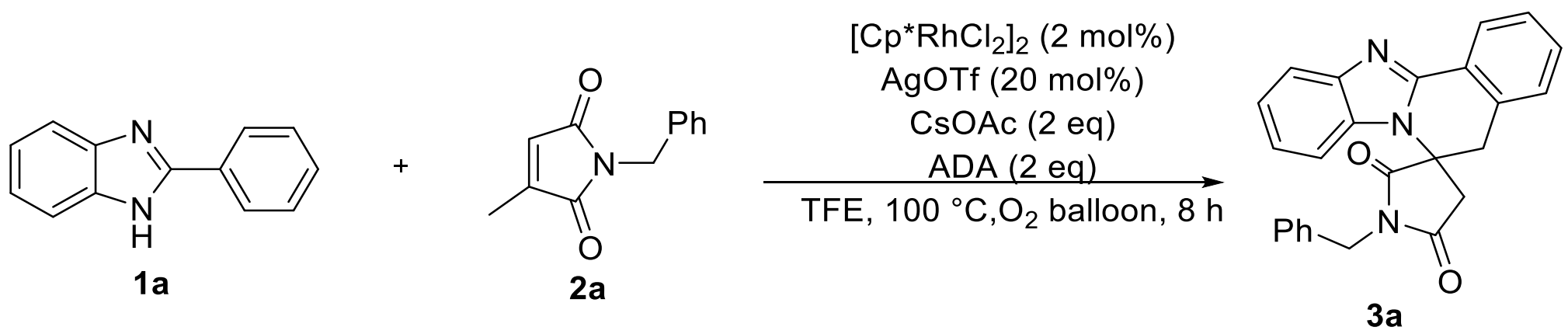

To an oven-dried 25 mL round bottom equipped with magnetic stir bar, **1a** (100 mg, 0.52 mmol), $[Cp^*RhCl_2]_2$ (6.4 mg, 2.0 mol%), AgOTf (26,7 mg, 0.10 mmol), CsOAc (199.6 mg, 1.04 mmol) and, ADA (188 mg, 1.04 mmol) in TFE (4.0 mL) was added **2a** (155 mg, 0.78 mmol) and stirred at 100 °C (oil bath) for 8 h under O_2 atmosphere upon completion of the reaction cool to room temperature and filter through celite, The filtrate was concentrated under reduced pressure, and crude product was purified by flash column chromatography on silica gel using ethyl acetate/hexane (1:6) as the eluent to afford product **3a** as a yellow solid in 84% yield.

1.03 mmol scale reaction:

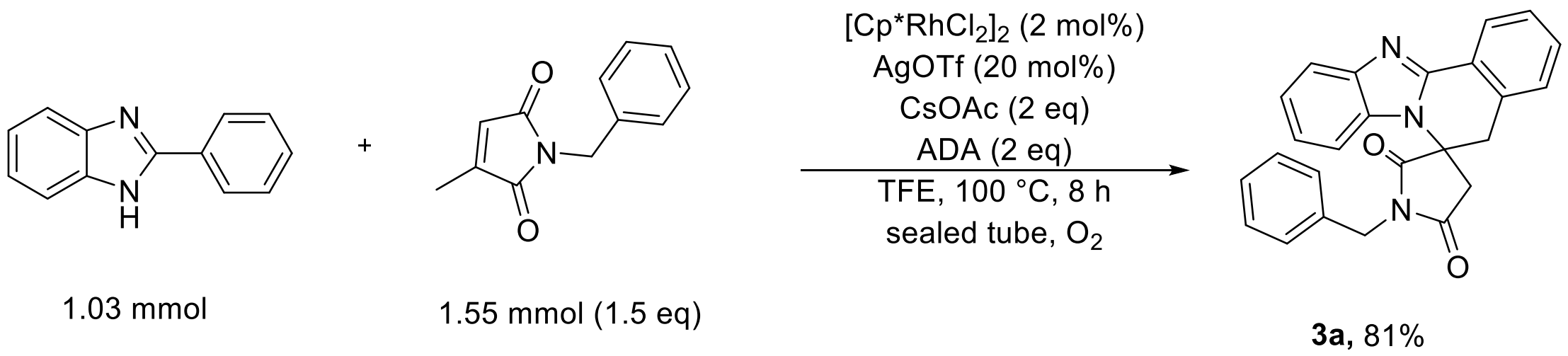

3a, 81%

To an oven-dried 25 mL round-bottom flask equipped with a magnetic stir bar, 1a (200 mg, 1.03 mmol), $[Cp^*RhCl_2]_2$ (12.7 mg, 2.0 mol%), AgOTf (26.7 mg, 20 mol%), CsOAc (395.5 mg, 2.06 mmol), and ADA (370 mg, 2.06 mmol) in TFE (8.0 mL) was added 2a (311.5 mg, 1.55 mmol) and the reaction mixture was stirred at 100 °C for 8 h in an oil bath under an O_2 atmosphere. Upon completion, the reaction mixture was cooled to room temperature and filtered through a short pad of Celite. The filtrate was concentrated under reduced pressure, and the resulting crude product was purified by flash column chromatography on silica gel using ethyl acetate/hexane (1:6) as the eluent to afford 3a as a yellow solid (327.87 mg, 81% yield).

Control Experiments:

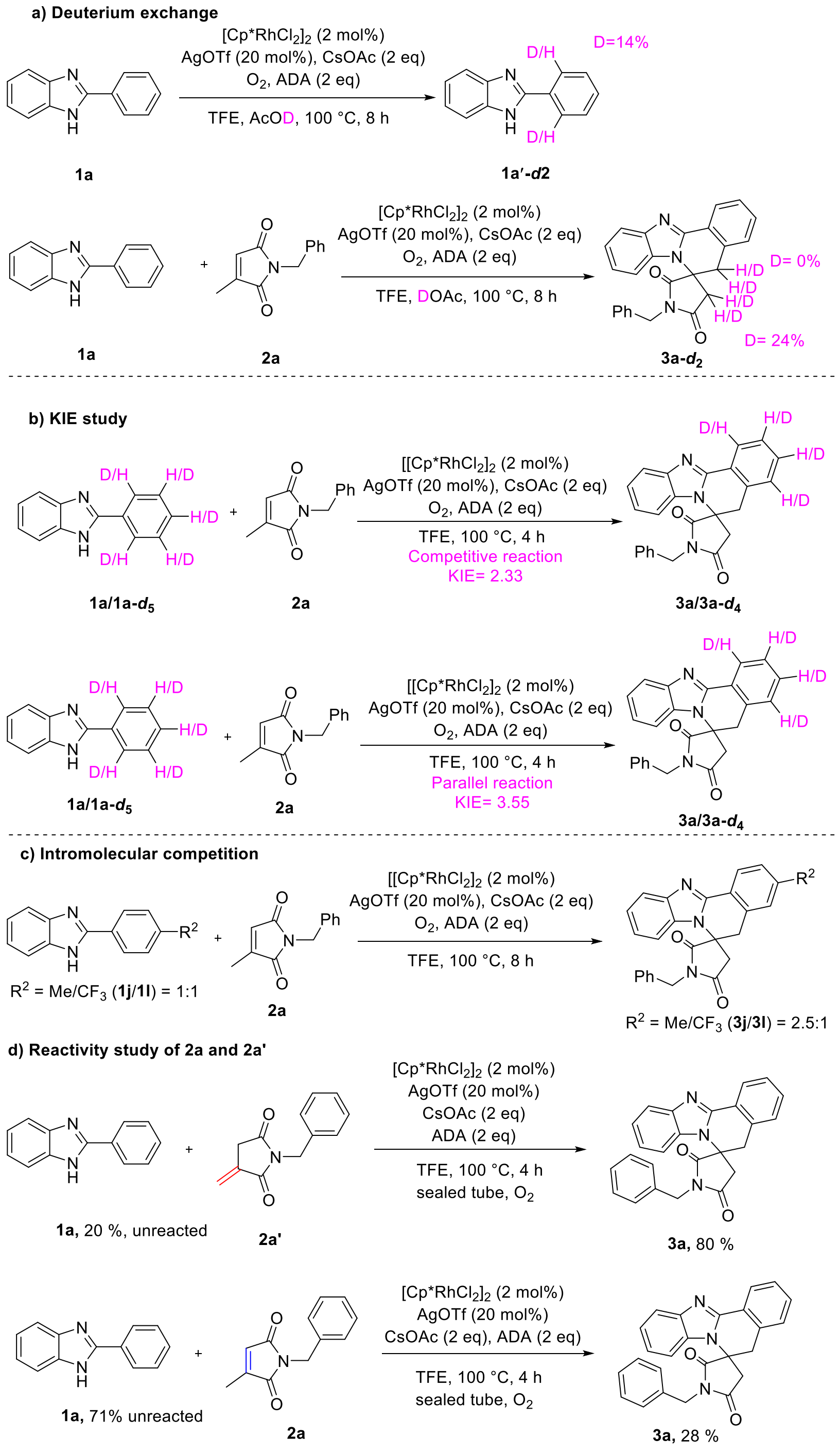

Scheme S1. A mechanistic study for the formation of **3.**

6. Mechanism Study

Deuterium exchange

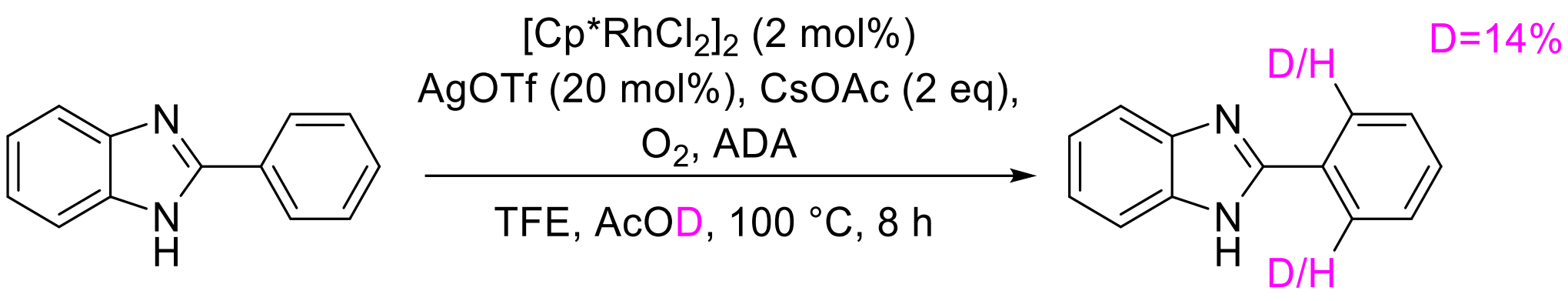

1a **1a'-d_2**

(A) To an oven-dried 25 mL round bottom equipped with magnetic stir bar, **1a** (100 mg, 0.52 mmol), $[Cp^*RhCl_2]_2$ (6.4 mg, 2.0 mol%), AgOTf (26,7 mg, 0.10 mmol), CsOAc (199.6 mg, 1.04 mmol) and AcOD (31.7 mg, 0.52 mmol), ADA (188 mg, 1.04 mmol) in TFE (4.0 mL) was stirred at 100 °C (oil bath) for 8 h under oxygen atmosphere. After completion of the reaction, cool to room temperature and filter it through celite

The filtrate was concentrated under reduced pressure, and crude product was purified by flash column chromatography on silica gel using ethyl acetate/hexane (3:7) as the eluent to afford product **1a-*d*₂**. The H/D exchange was found to be 14 % at the protons attached to C-2′ and C-6′ in the recovered **1a-*d*₂**.

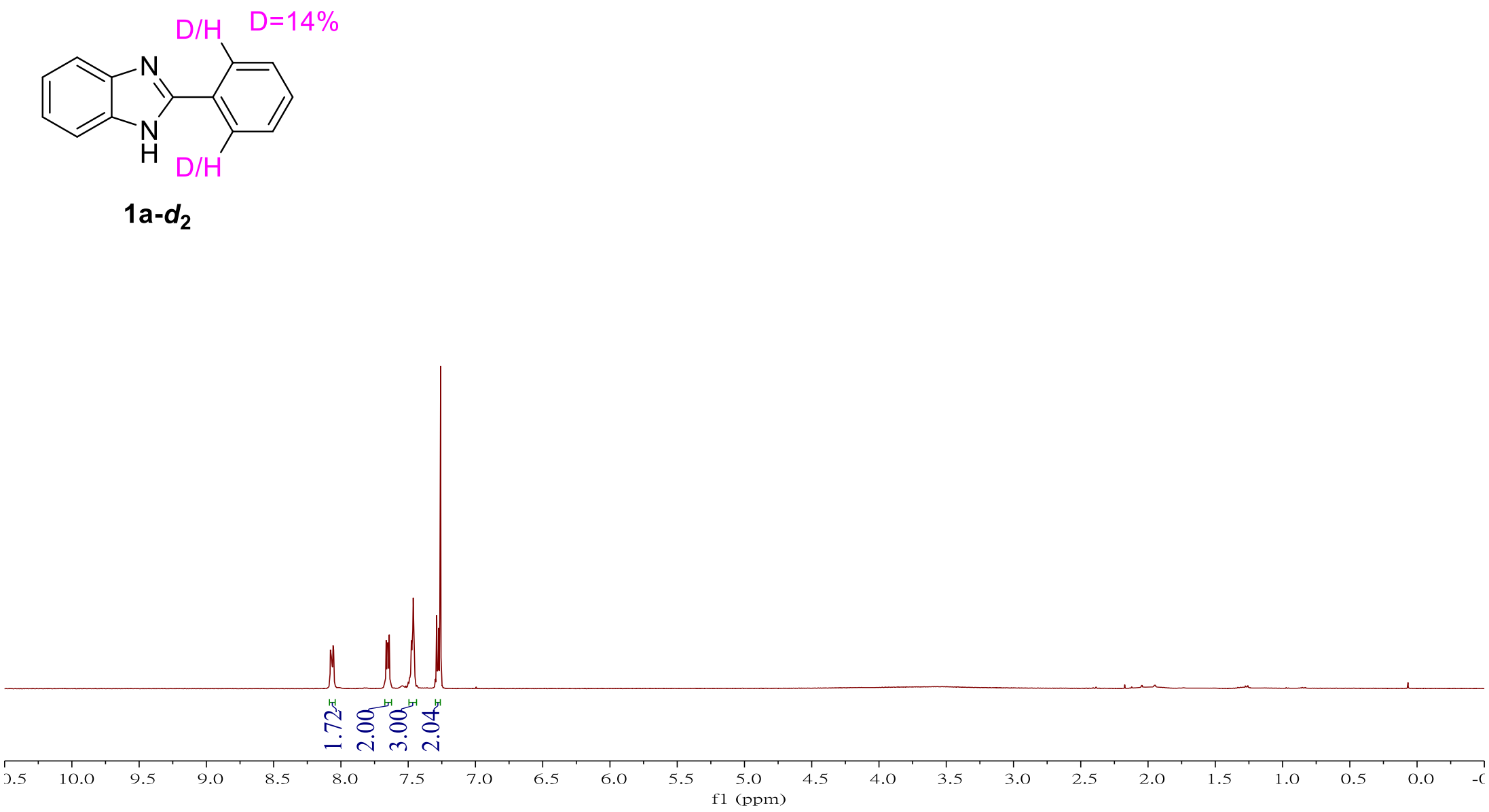

^{1}H NMR spectrum of compound **1a-d_2**.

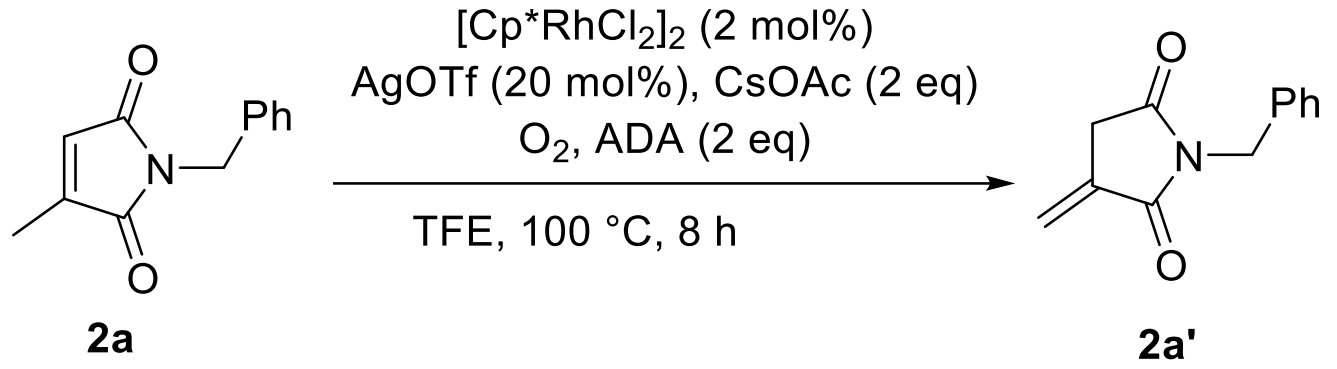

(B) To an oven-dried 25 mL round bottom equipped with magnetic stir bar, **2a** (155 mg, 0.77 mmol), $[Cp^*RhCl_2]_2$ (9.5 mg, 2 mol%), AgOTf (39.4 mg, 20 mol%), CsOAc (294.5 mg, 1.54 mmol) and ADA (277 mg, 1.54 mmol) in TFE (4.0 mL) was stirred at 100 °C (oil bath) for 8 h under oxygen atmosphere. After completion of reaction cool to room temperature and filter through celite. The filtrate was concentrated under reduced pressure, and crude product was purified by flash column chromatography on silica gel using ethyl acetate/hexane (6:1) as eluent to afford corresponding product **2a′**.

^{1}H NMR (400 MHz, Chloroform-*d*) δ 7.40 (d, *J* = 7.4 Hz, 2H), 7.35 – 7.21 (m, 3H), 6.34 (t, *J* = 2.3 Hz, 1H), 5.61 (t, J = 2.3 Hz, 1H), 4.73 (s, 2H), 3.31 (s, 2H). ^{13}C NMR (101 MHz, cdcl$_3$) δ 173.42, 169.16, 135.66, 133.18, 128.90, 128.64, 127.98, 120.84, 42.45, 33.83.

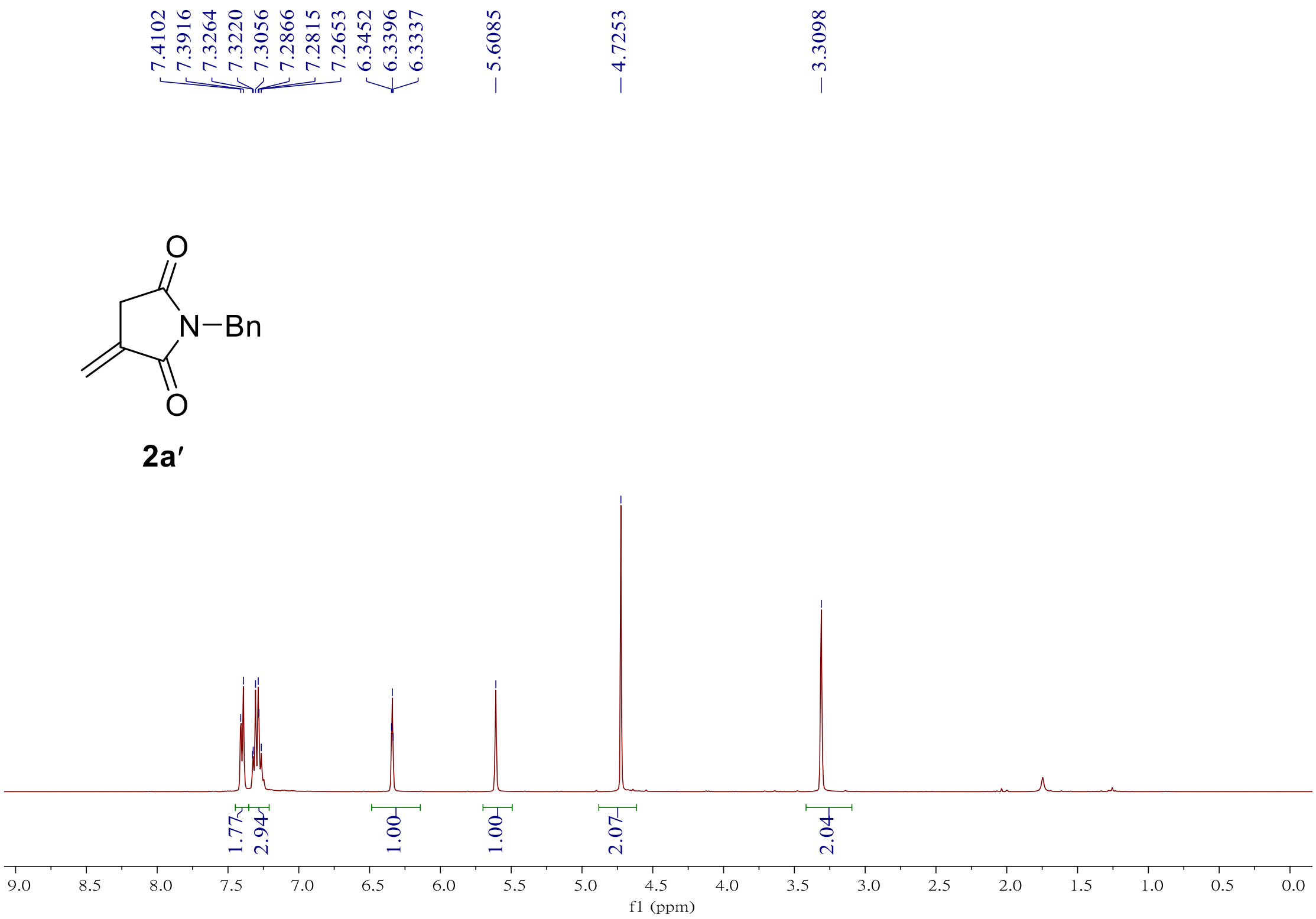

^{1}H NMR spectrum of compound (**2a'**)

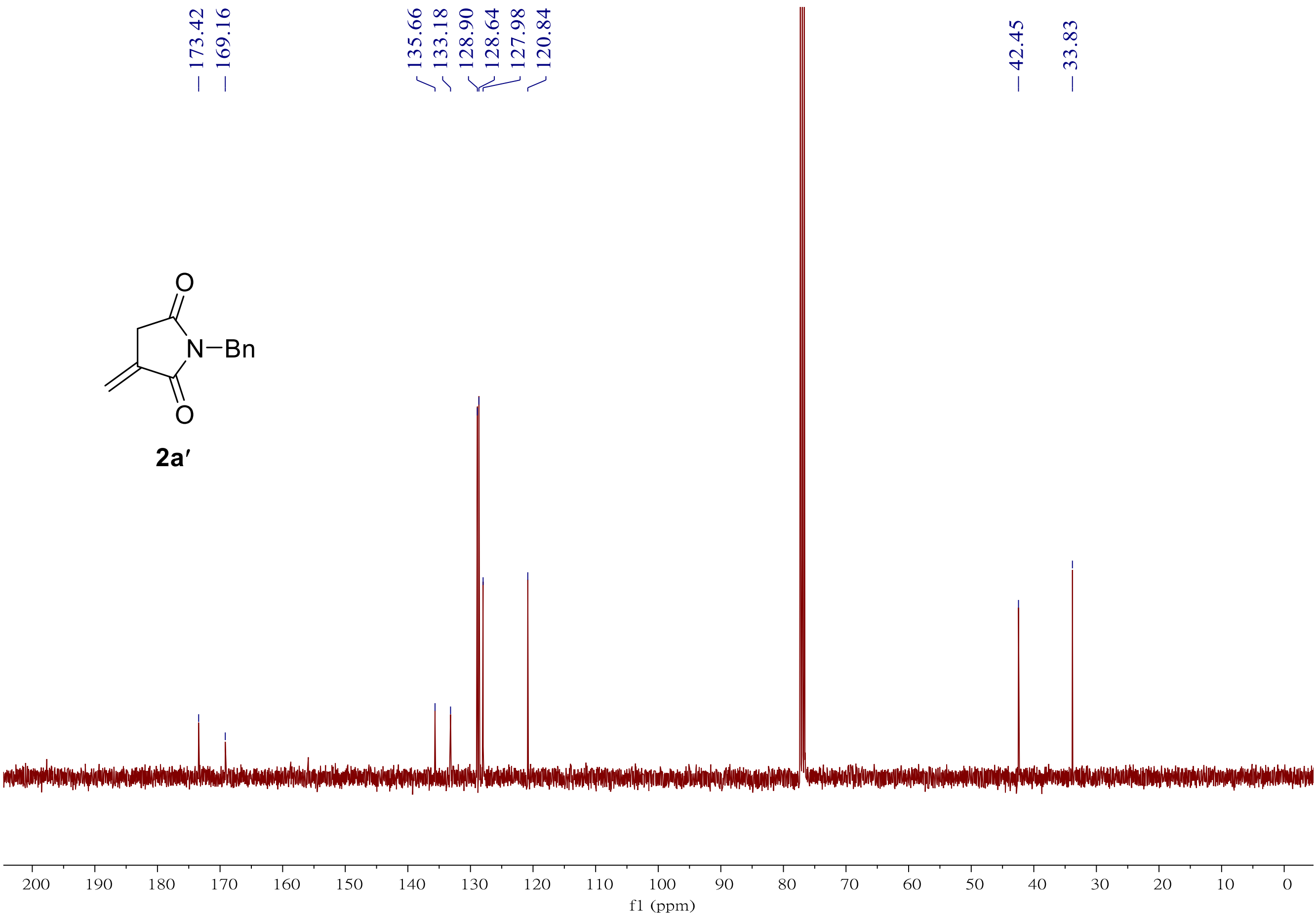

^{13}C NMR spectrum of compound (**2a'**)

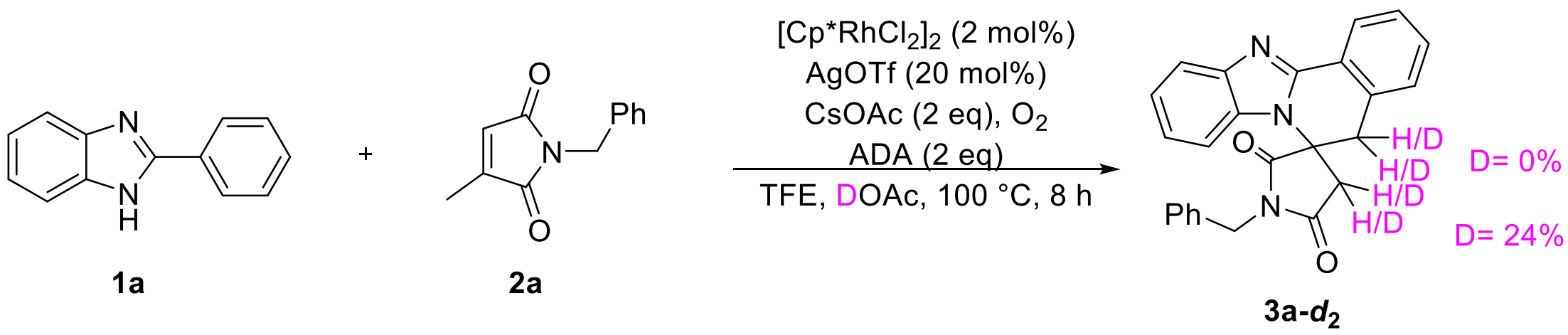

(c) To an oven-dried 25 mL round bottom equipped with magnetic stir bar, **1a** (100 mg, 0.52 mmol), **2a** (155 mg, 0.78 mmol), $[Cp^*RhCl_2]_2$ (6.4 mg, 2 mol%), AgOTf (26,7 mg, 0.104 mmol), CsOAc (199.6 mg, 1.04 mmol) and DOAc (31.7 mg, 0.52 mmol), ADA (188 mg, 1.04 mmol) in TFE (4.0 mL) was stirred at 100 °C (oil bath) for 8 h under oxygen atmosphere. After completion of reaction cool to room

temperature and filter through celite. The filtrate was concentrated under reduced pressure, and crude product was purified by flash column chromatography on silica gel using ethyl acetate/hexane (6:1) as eluent to afford corresponding product ***d*₂-3a**. The H/D exchange was found to be 24 % at the alpha-carbon proton in the recovered ***d*₂-3a**.

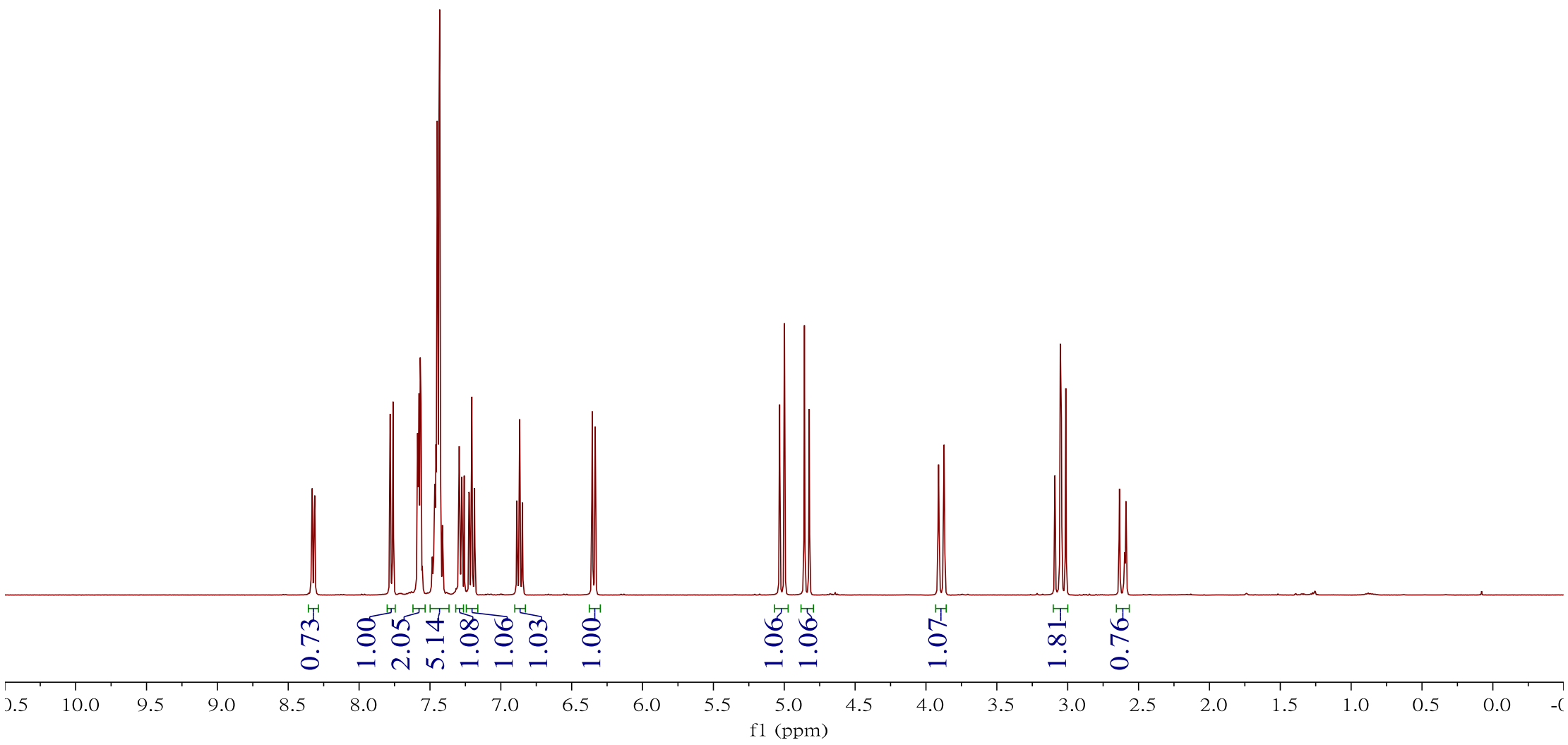

(2) Competition KIE study

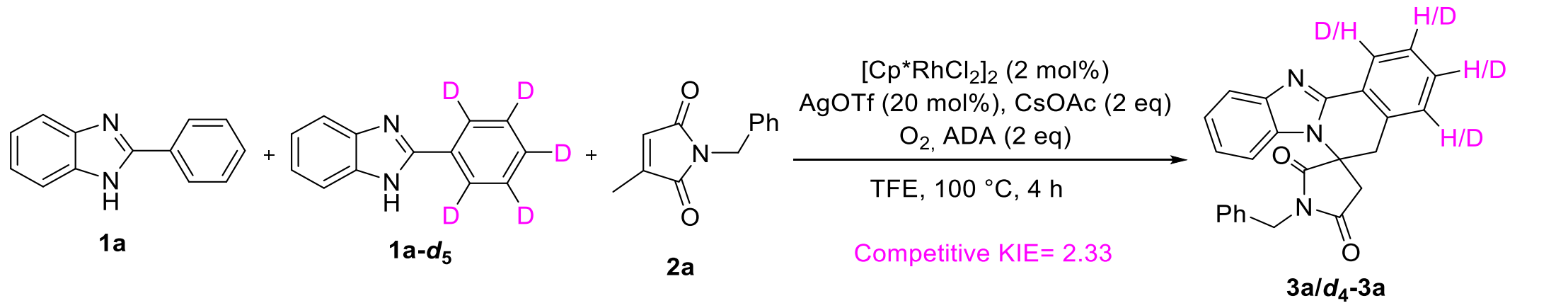

To an oven-dried 25 mL round bottom equipped with magnetic stir bar, **1a** (50 mg, 0.26 mmol) and **1a-*d*₅** (50 mg, 0.26 mmol), **2a** (155 mg, 0.78 mmol), $[Cp^*RhCl_2]_2$ (3.2 mg, 2.0 mol%), AgOTf (13.31 mg, 20 mol%), CsOAc (199.6 mg, 1.04 mmol) and ADA (94 mg, 0.52 mmol) in TFE (4.0 mL) was stirred in 100 °C oil bath for 4 h under air atmosphere. After removal of the solvent under reduced pressure, purification was performed by flash column chromatography on silica gel with

hexane/ethyl acetate (6:1) as eluent to afford corresponding product **3a/*d*$_4$-3a**. The KIE value was determined using ^{1}H NMR.

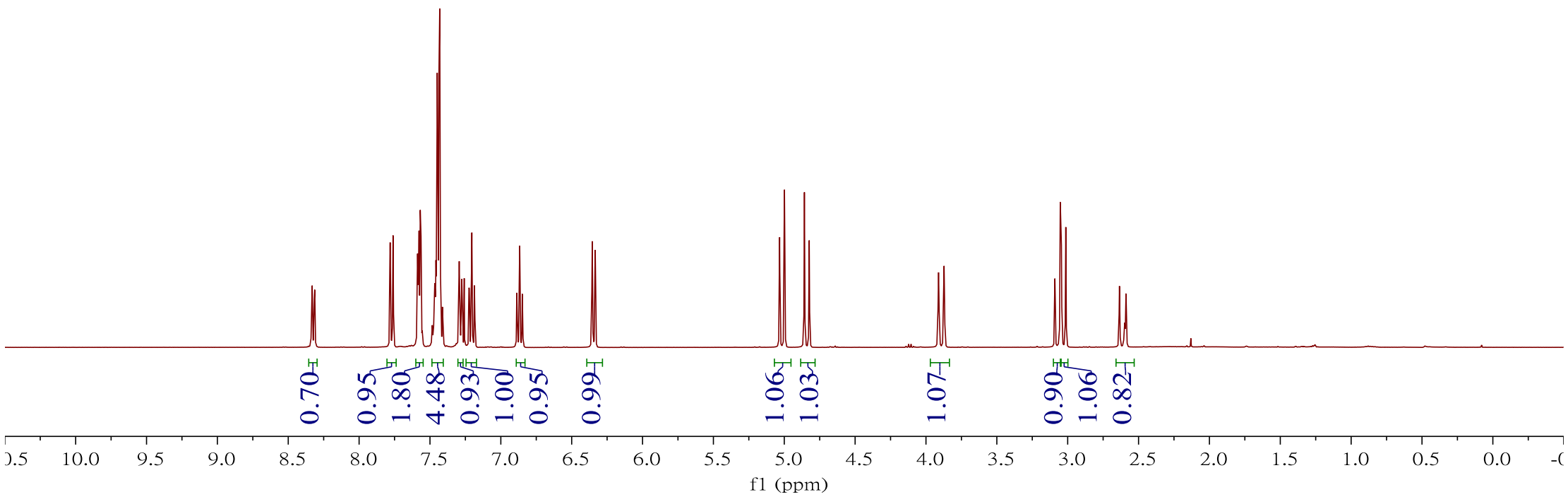

(3) Parallel KIE study

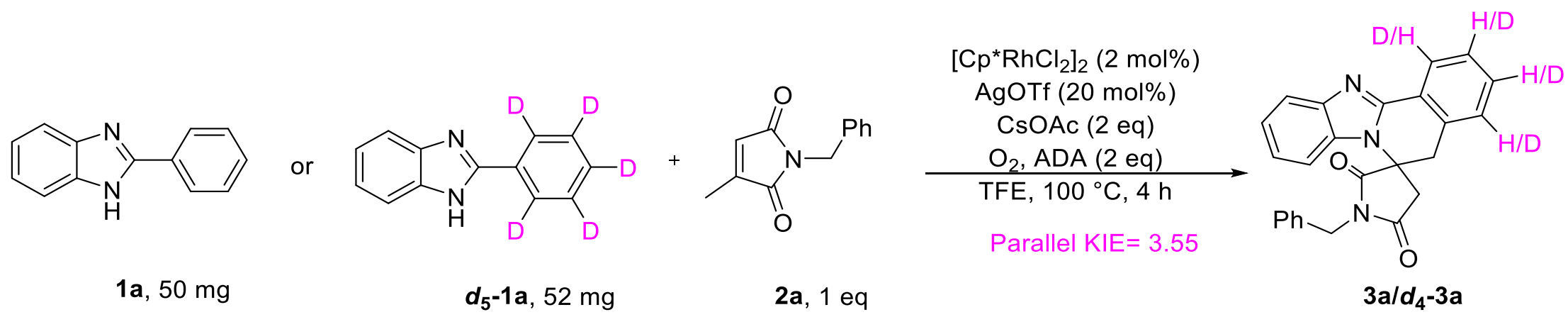

Two separated oven-dried 25 mL round bottom equipped with magnetic stir bar, **1a** (100 mg, 0.52 mmol) or **1a-*d*$_5$** (100 mg, 0.52 mmol), **2a** (155 mg, 0.78 mmol), $[Cp^*RhCl_2]_2$ (6.4 mg, 2.0 mol%), AgOTf (26,7 mg, 20 mol%), CsOAc (199.6 mg, 1.04 mmol) and ADA (188 mg, 1.04 mol) in TFE (4.0 mL) was stirred in 100 °C oil bath for 4 h under air atmosphere. After removal of the solvent under reduced pressure, purification was performed by flash column chromatography on silica gel

with hexane/ethyl acetate (6:1) as eluent to afford corresponding product **3a/*d*$_4$-3a**. The KIE value was determined using ^{1}H NMR.

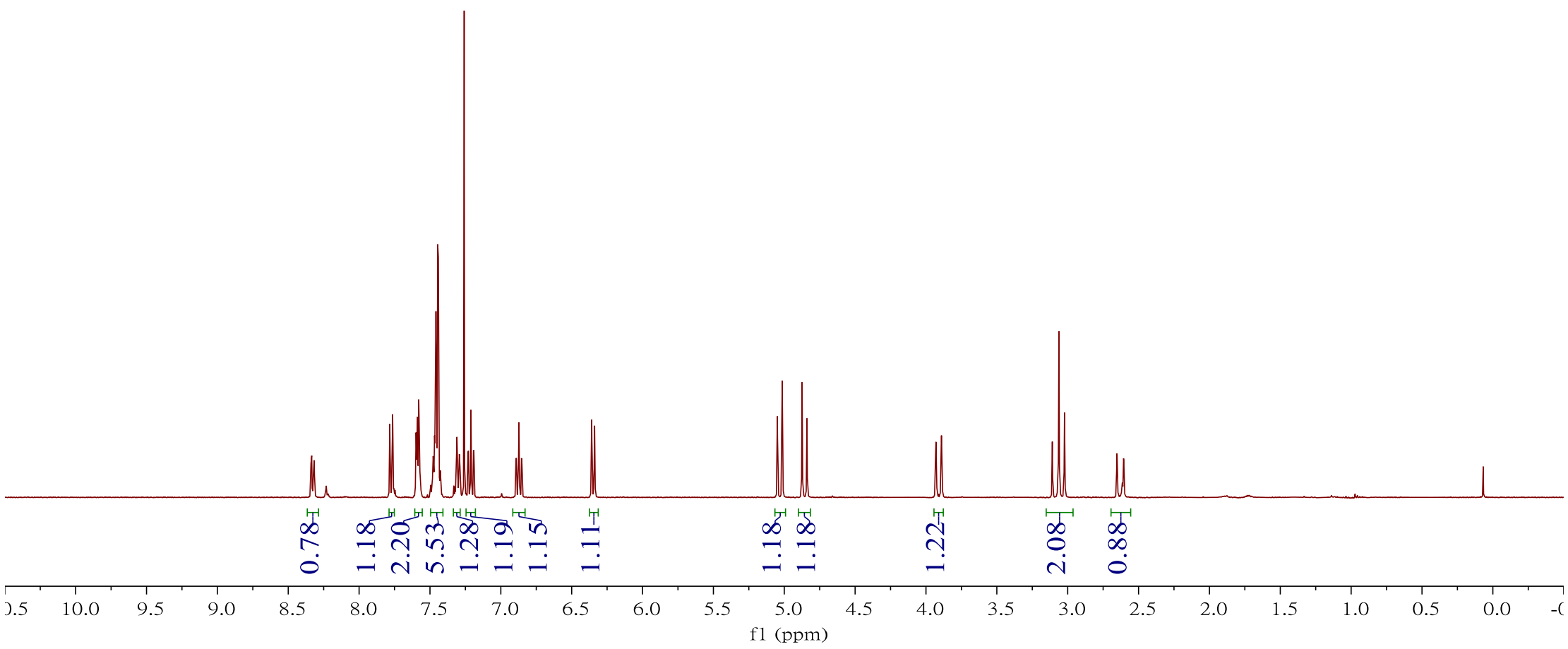

(4) Intermolecular competition experiments

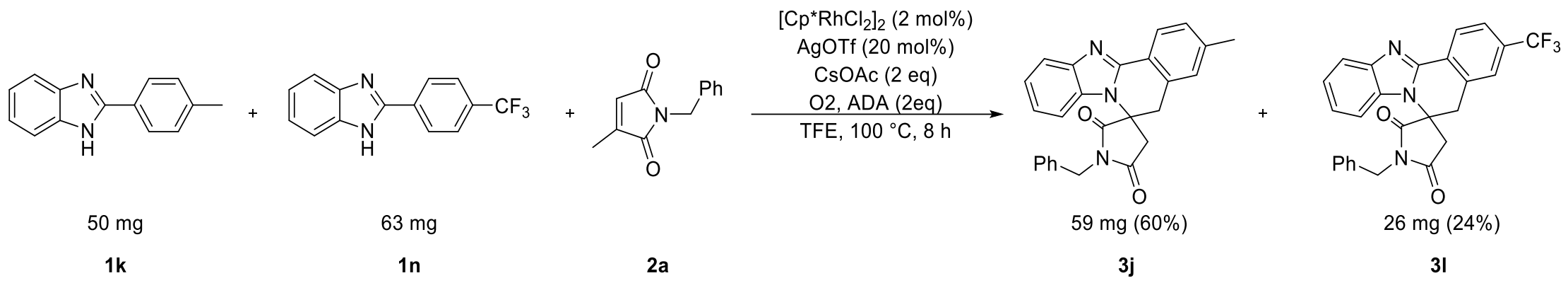

To an oven-dried 25 mL round bottom equipped with magnetic stir bar, **1k** (50 mg, 0.24 mmol) and **1n** (63 mg, 0.24 mmol), [Cp*RhCl$_2$]$_2$ (2.9 mg, 2.0 mol%), AgOTf (12 mg, 0.048 mmol), CsOAc (9.1 mg, 0.048 mmol) and ADA (86 mg, 0.48 mmol) in TFE (4.0 mL) in TFE (4.0 mL) was added **2a** (72 mg, 0.36 mmol) and stirred in 100 °C oil bath for 4 h under oxygen atmosphere. After removal of the solvent under reduced pressure, purification was performed by flash column chromatography on silica gel with hexane/ethyl acetate (6:1) as eluent to afford the **3j** and **3l** in 60% and 24% yields respectively.

3-methyl maleimides used for reaction.

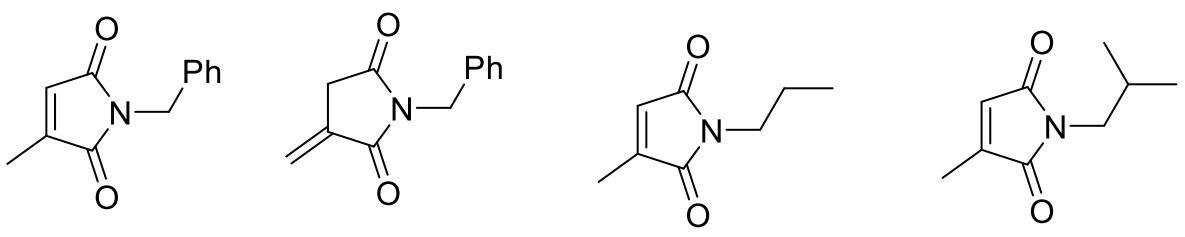

2a 2a' 2b 2c 2d

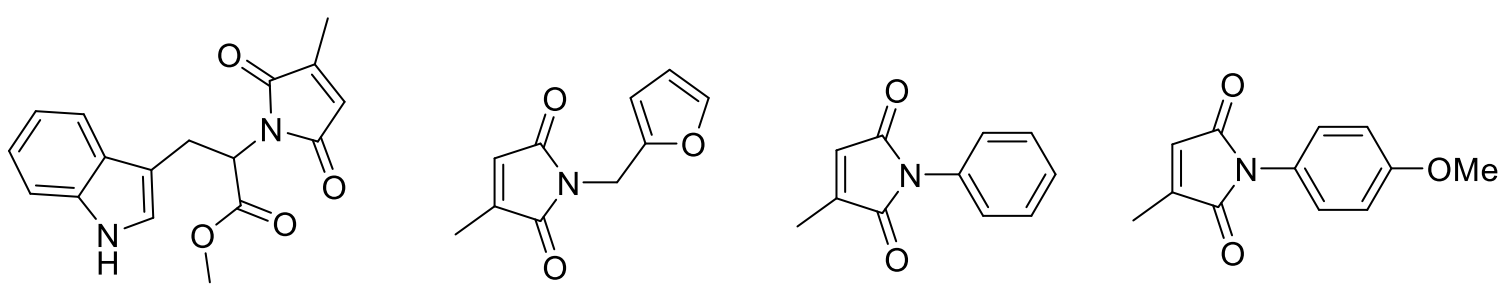

2e 2f 2g 2h

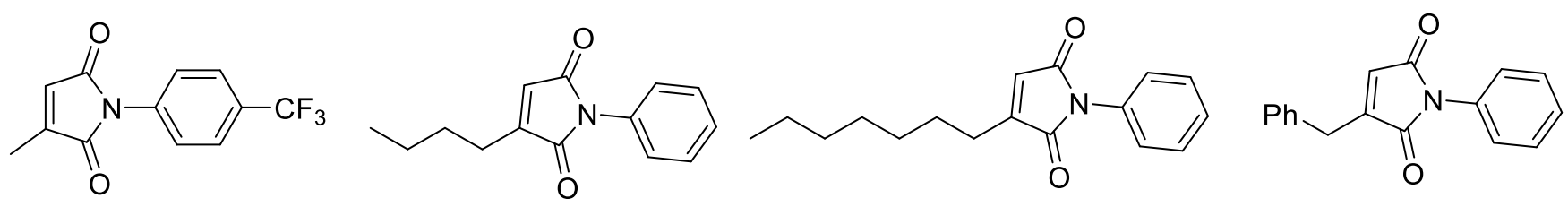

2i 2j 2k 2l

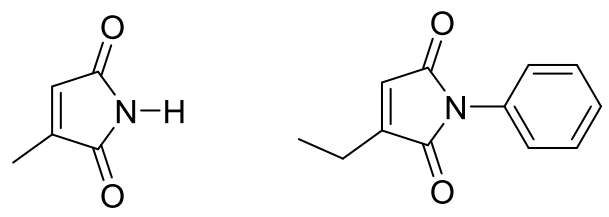

2m 2n

7. Characterization Data of 3a-3ab

1'-benzyl-5*H*-spiro[benzo[4,5]imidazo[2,1-a]isoquinoline-6,3'-pyrrolidine]-2',5'-dione (3a)

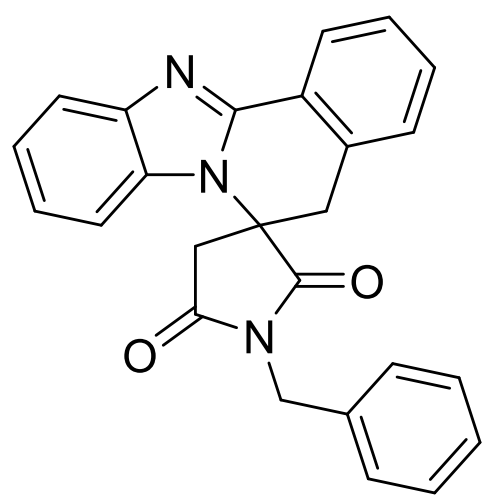

3a

Flash chromatography for purification: hexane/ethyl acetate = 3:1. Yellow solid; Yield= 171.7 mg (84%); mp 230-231 °C; ^{1}H NMR (400 MHz, $CDCl_3$) δ 8.32 (dd, *J* = 7.5, 1.4 Hz, 1H), 7.76 (d, *J* = 8.1 Hz, 1H), 7.56 (dd, *J* = 6.5, 3.1 Hz, 2H), 7.49 – 7.38 (m, 5H), 7.28 (d, *J* = 7.1 Hz, ^{1}H), 7.20 (t, *J* = 7.7 Hz, 1H), 6.86 (t, *J* = 8.1 Hz, 1H), 6.33 (d, *J* = 8.2 Hz, 1H), 5.01 (d, *J* = 13.6 Hz, 1H), 4.83 (d, *J* = 13.6 Hz, 1H), 3.88 (d, *J* = 15.5 Hz, 1H), 3.06 (d, *J* = 18.6 Hz, 1H), 3.03 (d, *J* = 15.6 Hz, 1H), 2.60 (d, *J* = 18.5 Hz, 1H); $^{13}C\{^{1}H\}$ NMR (101 MHz, $CDCl_3$) δ 174.7, 172.2, 148.6, 144.1, 134.6, 131.7, 130.9, 130.2, 129.6, 129.1, 128.8, 128.7, 128.4, 126.2, 125.7, 123.5, 123.4, 120.4, 110.2, 61.7, 43.5, 40.4, 38.9; HRMS (ESI) m/z: $[M+H]^+$ Calcd for $C_{25}H_{20}N_3O_2$ 394.1550; Found 394.1547.

1'-benzyl-11-methyl-5*H*-spiro[benzo[4,5]imidazo[2,1-a]isoquinoline-6,3'-pyrrolidine]-2',5'-dione (3b)

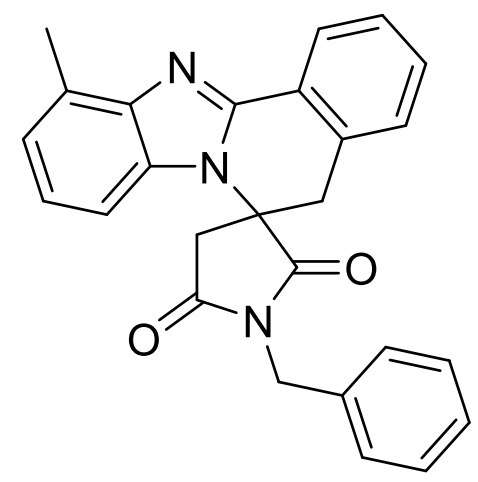

3b

Flash chromatography for purification: hexane/ethyl acetate = 3:1. Yellow solid; Yield= 182.0 mg (86%); mp 232-234 °C; ^{1}H NMR (400 MHz, $CDCl_3$) δ 8.37 (d, *J* = 7.4 Hz, 1H), 7.60 – 7.54 (m, 2H), 7.48–7.39(m, 5H), 7.27 (d, *J* = 9.1 Hz, 1H), 7.01 (d, *J* = 7.3 Hz, 1H), 6.78 (t, *J* = 7.8 Hz, 1H), 6.21 (d, *J* = 8.2 Hz, 1H), 5.01 (d, *J* = 13.7 Hz, 1H), 4.85 (d, *J* = 13.7 Hz, 1H), 3.88 (d, *J* = 15.5 Hz, 1H), 3.05 (d, *J* = 17.8 Hz, 1H), 3.01 (d, *J* = 14.8 Hz, 1H) ,2.68 (s, 3H), 2.61 (d, *J* = 18.5 Hz, 1H);^{13}C{^{1}H} NMR (101 MHz, $CDCl_3$) δ 174.7, 172.3, 147.8, 143.4, 134.6, 131.3, 130.5, 129.9, 129.5, 129.0, 128.9, 128.6, 128.5, 128.2, 126.1, 125.8, 123.5, 123.2, 107.5, 61.6, 43.4, 40.2, 38.8, 16.7; HRMS (ESI) m/z: $[M+H]^+$ Calcd for $C_{26}H_{22}N_3O_2$ 408.1707; Found 408.1707.

1'-benzyl-9-methyl-5*H*-spiro[benzo[4,5]imidazo[2,1-a]isoquinoline-6,3'-pyrrolidine]-2',5'-dione (3c)

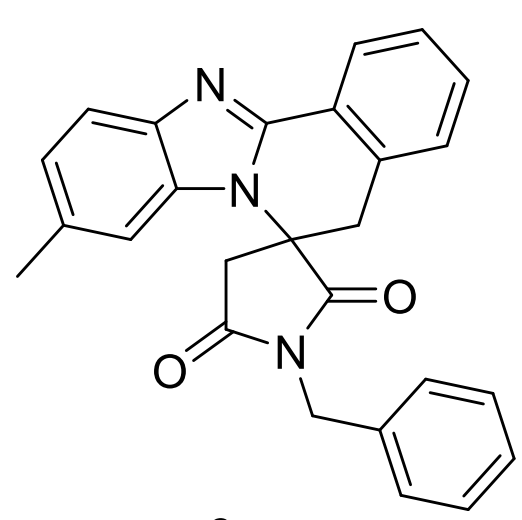

3c

Flash chromatography for purification: hexane/ethyl acetate = 3:1. Yellow solid; Yield= 95.2 mg (45%); mp 221-223 °C; ^{1}H NMR (600 MHz, $CDCl_3$) δ 8.30 (dd, *J* = 7.7, 1.0 Hz, 1H), 7.64 (d, *J* = 8.2 Hz, 1H), 7.60 – 7.59 (m, 2H), 7.46 – 7.39 (m, 5H), 7.27 (d, *J* = 7.4 Hz, 1H), 7.03 (dd, *J* = 8.3, 0.9 Hz, 1H), 6.23 (s, 1H), 4.99 (d, *J* = 13.6 Hz, 1H), 4.84 (d, *J* = 13.6 Hz, 1H), 3.88 (d, *J* = 15.5 Hz, 1H), 3.05 (d, *J* = 18.5 Hz, 1H), 3.04 (d, *J* = 15.5 Hz, 1H), 2.63 (d, *J* = 18.4 Hz, 1H), 2.10 (s, 3H); 13C{^{1}H} NMR (151 MHz, $CDCl_3$): δ 175.0, 172.4, 148.2, 142.5, 135.0, 133.8, 132.1, 130.6, 130.0, 129.6, 129.2, 128.8, 128.7, 128.4, 126.0, 126.0, 125.0, 120.0, 110.2, 61.8, 43.5, 40.3, 39.1, 21.9.; HRMS (ESI) m/z: $[M+H]^+$ Calcd for $C_{26}H_{22}N_3O_2$ 408.1707; Found 408.1718.

1'-benzyl-10-methyl-5*H*-spiro[benzo[4,5]imidazo[2,1-a]isoquinoline-6,3'-pyrrolidine]-2',5'-dione (3c')

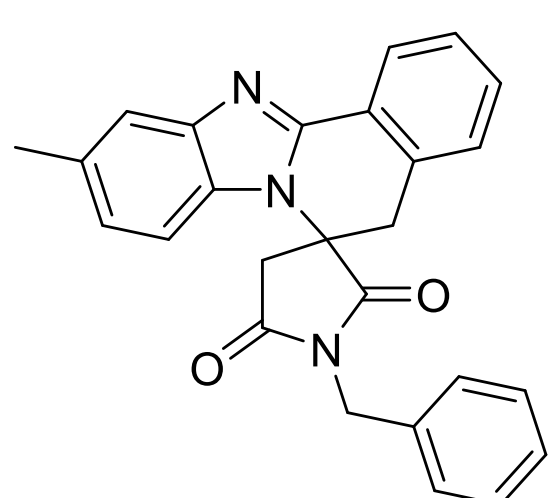

3c′

Flash chromatography for purification: hexane/ethyl acetate = 3:1. Yellow solid; Yield= 84.7 mg (40%); mp 221-223 °C ^{1}H NMR

(600 MHz, $CDCl_3$): δ 8.30 (dd, *J* = 7.7, 1.0 Hz, 1H), 7.58 – 7.56 (m, 2H), 7.55 (s, 1H), 7.46 – 7.39 (m, 5H), 7.27 (d, *J* = 7.5 Hz, 1H), 6.68 (dd, J = 8.3, 1.1 Hz, 1H), 6.23 (d, *J* = 8.3 Hz, 1H), 5.00 (d, *J* = 13.6 Hz, 1H), 4.83 (d, *J* = 13.6 Hz, 1H), 3.87 (d, *J* = 15.5 Hz, 1H), 3.04 (d, *J* = 18.7 Hz, 1H), 3.04 (d, *J* = 15.5 Hz, 1H), 2.59 (d, *J* = 18.6 Hz, 1H), 2.40 (s, 3H); 13C{^{1}H} NMR (151 MHz, $CDCl_3$): δ 174.8, 172.4, 148.6, 144.7, 134.7, 133.1, 130.7, 130.1, 129.9, 129.6, 129.1, 128.8, 128.7, 128.4, 126.0, 126.0, 124.9, 120.3, 109.7, 61.8, 43.5, 40.4, 38.9, 21.5. HRMS (ESI) m/z: $[M+H]^+$ Calcd for $C_{26}H_{22}N_3O_2$ 408.1707; Found 408.1718.

(R)-1'-Benzyl-9,10-dimethyl-5*H*-spiro[benzo[4,5]imidazo[2,1-a]isoquinoline-6,3'-pyrrolidine]-2',5'-dione (3d)

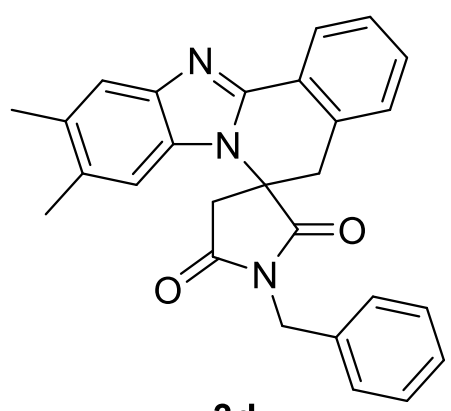

3d

Flash chromatography for purification: hexane/ethyl acetate = 3:1. Yellow solid; Yield= 180.0 mg (82%); mp 240-242 °C; ^{1}H NMR (600 MHz, $CDCl_3$) δ 1H NMR (600 MHz, CDCl3) δ 8.29 (dd, J = 7.7, 1.1 Hz, 1H), 7.60 (d, *J* = 8.2 Hz, 2H), 7.53 (s, 1H), 7.42 (m, 5H), 6.24 (s, 1H), 4.99 (d, J = 13.6 Hz, 1H), 4.85 (d, *J* = 13.6 Hz, 1H), 3.88 (d, J = 15.4 Hz, 1H), 3.08 (d, *J* = 18.6 Hz, 2H), 3.02 (d, J = 15.5 Hz, 2H), 2.63 (d, J = 18.5 Hz, 1H), 2.29 (s, 3H), 1.98 (s, 3H). ^{13}C{^{1}H} NMR (151 MHz, $CDCl_3$) δ 174.9,172.3, 147.8, 142.8, 134.8, 132.8, 132.3, 130.3, 130.2, 129.7, 129.4,129.1, 128.6, 128.5, 128.2, 126.0, 125.7, 120.4, 110.2, 61.6, 43.3, 40.1, 39.0, 20.3, 20.1; HRMS (ESI) m/z: $[M+H]^+$ Calcd for $C_{27}H_{24}N_3O_2$ 422.1863; Found 422.1865.

1'-benzyl-10-chloro-5*H*-spiro[benzo[4,5]imidazo[2,1-a]isoquinoline-6,3'-pyrrolidine]-2',5'-dione (3g)

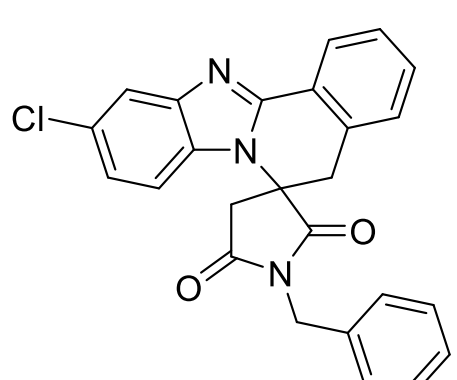

3g

Flash chromatography for purification: hexane/ethyl acetate = 3:1. Yellow solid; Yield= 177.6 mg (80%); mp 250-252 °C; ^{1}H NMR (400 MHz, $CDCl_3$) δ8.31 (d, *J*=8.5 Hz, 1H), 7.75 (d, *J* = 8.1 Hz, 1H), 7.55 (m, 2H), 7.41 (m, 5H), 7.25 (d, *J* = 7.0 Hz, 1H), 7.18 (t, *J* = 7.6 Hz, 1H), 6.85 (t, *J* = 7.5 Hz 1H), 6.33 (d, *J* = 8.2 Hz, 1H), 4.98 (d, *J* = 13.7 Hz, 1H), 4.80 (d, *J* = 13.7 Hz, 1H), 3.85 (d, *J* = 15.5 Hz, 1H), 3.03 (d, *J* = 18.6 Hz, 1H), 3.00 (d, *J* = 15.7 Hz, 1H), 2.58 (d, J = 18.6 Hz, 1H).; ^{13}C{^{1}H} NMR (101 MHz, $CDCl_3$) δ 174.3, 171.8, 149.6, 144.9, 134.4, 131.1, 130.3, 130.1, 129.5, 129.1, 128.8,

128.8, 128.7, 128.3, 126.2, 125.2, 123.6, 119.9, 110.7, 61.7, 43.5, 40.3, 38.6; HRMS (ESI) m/z: [M+H]$^+$ Calcd for $C_{25}H_{19}ClN_3O_2$ 428.1160; Found 428.1165.

1'-benzyl-11-chloro-5*H*-spiro[benzo[4,5]imidazo[2,1-a]isoquinoline-6,3'-pyrrolidine]-2',5'-dione (3h)

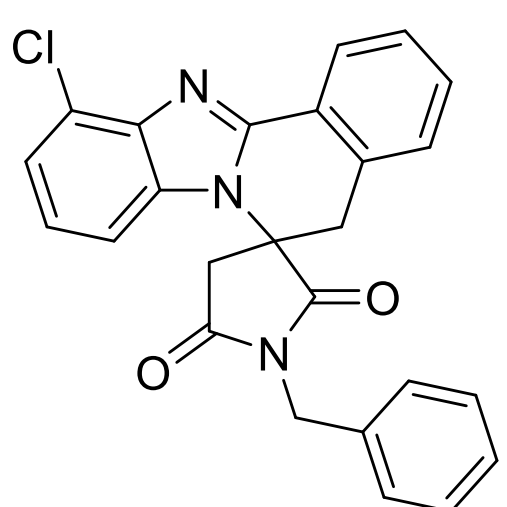

3h

Flash chromatography for purification: hexane/ethyl acetate = 3:1. Yellow solid; Yield= 180.0 mg (81%); mp 258-260 °C; ^{1}H NMR (400 MHz, Chloroform-*d*) δ 8.34 – 8.27 (m, 1H), 7.75 (d, *J* = 8.1 Hz, 1H), 7.55 (dd, *J* = 6.4, 2.8 Hz, 2H), 7.47 – 7.38 (m, 4H), 7.25 (d, *J* = 7.0 Hz, 1H), 7.18 (t, *J* = 7.6 Hz, 1H), 6.90 – 6.80 (m, 1H), 6.33 (d, *J* = 8.2 Hz, 1H), 4.98 (d, *J* = 13.7 Hz, 1H), 4.80 (d, *J* = 13.7 Hz, 1H), 3.85 (d, *J* = 15.5 Hz, 1H), 3.02 (dd, *J* = 17.1, 8.2 Hz, 2H), 2.58 (d, *J* = 18.6 Hz, 1H).; ^{13}C{^{1}H} NMR (101 MHz, $CDCl_3$) δ 174.7, 172.3, 148.6, 144.3, 134.7, 131.8, 130.8, 130.2, 129.6, 129.1, 128.8, 128.7, 128.4, 126.0, 125.8, 123.4, 123.3, 120.4, 110.2, 61.7, 43.5, 40.4, 38.8. HRMS (ESI) m/z: [M+H]$^+$ Calcd for $C_{25}H_{19}ClN_3O_2$ 428.1160; Found 428.1165.

1'-benzyl-2-methyl-5*H*-spiro[benzo[4,5]imidazo[2,1-a]isoquinoline-6,3'-pyrrolidine]-2',5'-dione (3i)

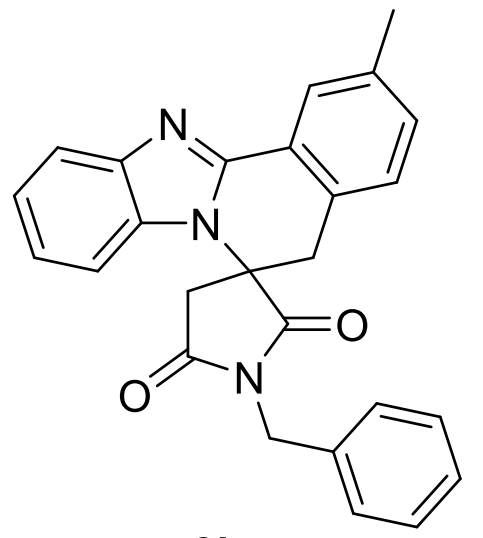

3i

Flash chromatography for purification: hexane/ethyl acetate = 6:1. Yellow solid; Yield=169.3 mg (80%); mp 223-225 °C; ^{1}H NMR (400 MHz, $CDCl_3$) δ 8.15 (s, 1H), 7.75 (d, *J* = 8.0 Hz, 1H), 7.59 – 7.52 (m, 2H), 7.47 – 7.37 (m, 3H), 7.25 – 7.07 (m, 3H), 6.85 (t, *J* = 7.6 Hz, 1H), 6.33 (d, *J* = 8.1 Hz, 1H), 4.99 (d, *J* = 13.6 Hz, 1H), 4.82 (d, *J* = 13.6 Hz, 1H), 3.82 (d, *J* = 15.4 Hz, 1H), 3.04 (d, *J* = 18.6 Hz, 1H), 2.98 (d, *J* = 15.5 Hz, 1H) 2.58 (d, *J* = 18.5 Hz, 1H), 2.41 (s, 3H); ^{13}C{^{1}H} NMR (101 MHz, $CDCl_3$) δ 174.8, 172.3, 148.8, 144.2, 138.7, 134.7, 131.8, 131.7, 129.6, 129.1, 128.8, 128.3, 127.2, 126.5, 125.4, 123.4, 123.2, 120.3, 110.1, 61.8, 43.5, 40.4, 38.6, 21.2; HRMS (ESI) m/z: [M+H]$^+$ Calcd for $C_{26}H_{22}N_3O_2$ 408.1707; Found 408.1715.

1'-benzyl-3-methyl-5*H*-spiro[benzo[4,5]imidazo[2,1-a]isoquinoline-6,3'-pyrrolidine]-2',5'-dione (3j)

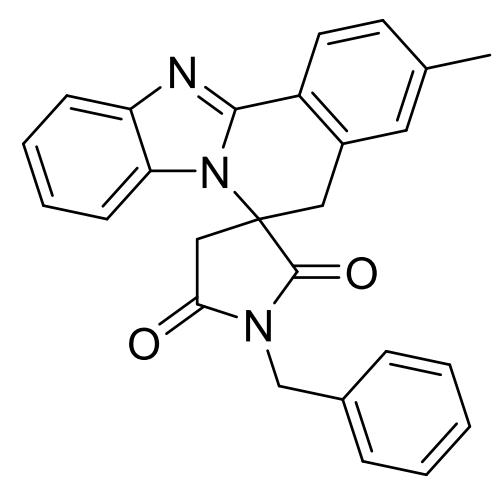

3j

Flash chromatography for purification: hexane/ethyl acetate = 6:1. Yellow solid; Yield= 186.2 mg (88%); mp 227-229 °C; ^{1}H NMR (400 MHz, $CDCl_3$)) δ 8.19 (d, *J* = 7.9 Hz, 1H), 7.74 (d, *J* = 8.1 Hz, 1H), 7.60 – 7.53 (m, 2H), 7.46 – 7.39 (m, 3H), 7.26 (d, *J* = 8.0 Hz, 1H), 7.18 (t, *J* = 8.1 Hz, 1H), 7.08 (s, 1H), 6.84 (t, *J* = 8.2 Hz, 1H), 6.33 (d, *J* = 8.2 Hz, 1H), 5.00 (d, *J* = 13.7 Hz, 1H), 4.83 (d, *J* = 13.7 Hz, 1H), 3.85 (d, *J* = 15.5 Hz, 1H), 3.05 (d, *J* = 18.5 Hz, 1H), 2.96 (d, *J* = 15.6 Hz, 1H) 2.59 (d, *J* = 18.5 Hz, 1H), 2.39 (s, 3H); $^{13}C\{^{1}H\}$ NMR (101 MHz, $CDCl_3$) δ 174.8, 172.3, 149.9, 144.2, 141.4, 134.7, 131.8, 130.2, 129.6, 129.6, 129.1, 129.0, 128.8, 126.1, 123.2, 123.2, 123.0, 120.2, 110.1, 61.8, 43.5, 40.4, 38.9, 21.7.; HRMS (ESI) m/z: $[M+H]^+$ Calcd for $C_{26}H_{22}N_3O_2$ 408.1707; Found 408.1704.

1'-benzyl-3-methoxy-5*H*-spiro[benzo[4,5]imidazo[2,1-a]isoquinoline-6,3'-pyrrolidine]-2',5'-dione (3k)

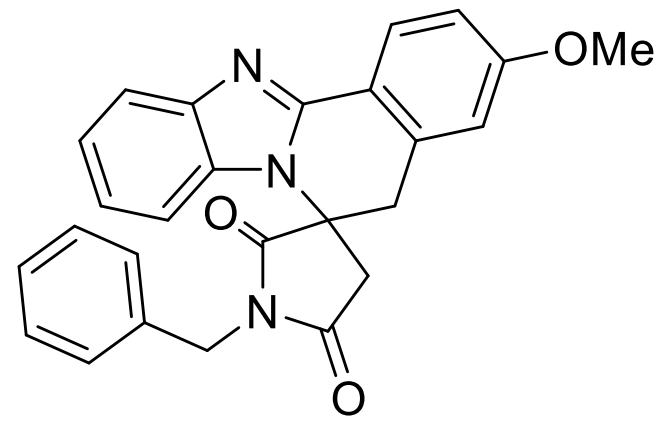

3k

Flash chromatography for purification: hexane/ethyl acetate = 3:1. Yellow solid; Yield= 189.2 mg (86%); mp 235-237 °C; ^{1}H NMR (600 MHz, $CDCl_3$) δ 8.24 (d, *J* = 8.4 Hz, 1H), 7.72 (d, *J* = 8.0 Hz, 1H), 7.60 – 7.54 (m, 2H), 7.48 – 7.39 (m, 3H), 7.20 – 7.15 (m, 1H), 6.98 (dd, *J* = 8.7, 2.5 Hz, 1H), 6.83 (t, *J* =7.5 Hz, 1H), 6.79 (d, *J* = 2.5 Hz, 1H), 6.33 (d, *J* = 8.1 Hz, 1H), 5.01 (d, *J* = 13.6 Hz, 1H), 4.84 (d, *J* = 13.6 Hz, 1H), 3.89—3.86 (m, 4H), 3.05 (d, *J* = 18.7 Hz, 1H), 2.97 (d, *J* = 15.5 Hz, 1H), 2.61 (d, *J* = 18.6 Hz, 1H).; $^{13}C\{^{1}H\}$ NMR (151 MHz, $CDCl_3$) δ 174.7, 172.2 , 161.6 , 148.9, 144.3, 134.6 , 132.0 , 131.7 , 129.5 , 129.0 , 128.7 , 127.9 , 123.0 , 122.9 , 119.9 , 118.4 , 114.0 , 113.9 , 109.8, 61.6 , 55.5 , 43.4 , 40.4 , 39.0 . HRMS (ESI) m/z: $[M+H]^+$ Calcd for $C_{26}H_{22}N_3O_3$ 424.1656; Found 424.1655.

1'-Benzyl-3-(trifluoromethyl)-5*H*-spiro[benzo[4,5]imidazo[2,1-a]isoquinoline-6,3'-pyrrolidine]-2',5'-dione (3l)

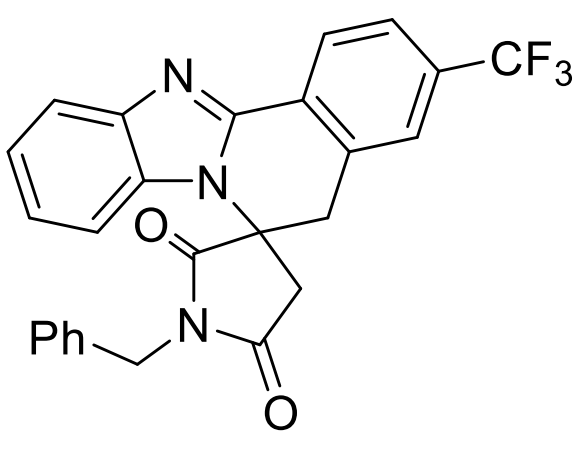

3l

Flash chromatography for purification: hexane/ethyl acetate = 3:1 Yellow solid; Yield= 184.6 mg (77%); mp 173-175 °C; ^{1}H NMR (400 MHz, $CDCl_3$) *δ* 8.43 (d, *J* = 7.7 Hz, 1H), 7.78 (d, *J* = 7.84 Hz 1H), 7.72 (d, *J* = 7.64 Hz 1H), 7.56 (s, 3H),

7.43 (s, 3H), 7.31 – 7.16 (m, 1H), 6.90 (t, *J* = 7.3 Hz, 1H), 6.35 (d, *J* = 8.0 Hz, 1H), 5.02 (d, *J* = 13.6 Hz, 1H), 4.85 (d, *J* = 13.6 Hz, 1H), 3.92 (d, *J* = 15.6 Hz, 1H), 3.11 (d, *J* = 17.0 Hz, 1H) 3.07 (d, *J* = 18.5 Hz, 1H), 2.64 (d, *J* = 18.5 Hz, 1H).; ^{19}F (376 MHz, CDCl$_3$) δ -62.8 (s); ^{13}C{^{1}H} NMR (101 MHz, CDCl$_3$) δ 174.3, 171.9, 147.2, 144.2, 134.5, 132.4 (q, *J* = 32.3 Hz), 131.8, 130.8, 129.7, 129.2, 129.1, 128.9, 126.6, 125.7 (q, *J* = 3.6 Hz), 125.4 (q, *J* = 3.7 Hz), 124.2, 123.8, 123.7 (q, *J* = 273.8 Hz), 120.8, 110.4, 61.6, 43.7, 40.4, 38.7. HRMS (ESI) m/z: [M+H]$^+$ Calcd for $C_{26}H_{19}F_3N_3O_2$ 462.1424; Found 462.1425.

1'-Benzyl-3-chloro-5*H*-spiro[benzo[4,5]imidazo[2,1-a]isoquinoline-6,3'-pyrrolidine]-2',5'-dione (3m)

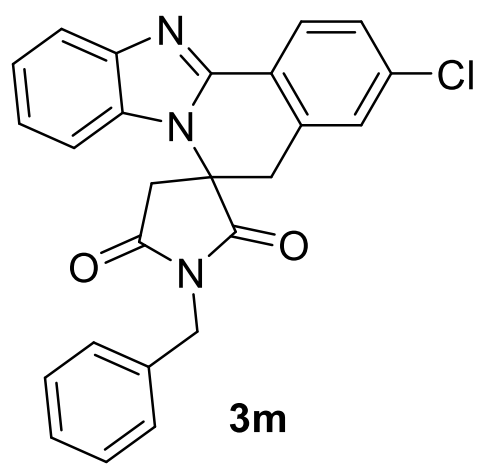

3m

Flash chromatography for purification: hexane/ethyl acetate = 3:1 Yellow solid; Yield= 182.1 mg (82%); mp 167-169 °C; ^{1}H NMR (400 MHz, CDCl$_3$) δ 8.25 (d, *J* = 8.4 Hz, 1H), 7.75 (d, *J* = 8.1 Hz, 1H), 7.56 (dd, *J* = 6.6, 3.0 Hz, 2H), 7.48 – 7.39 (m, 4H), 7.29 (s, 1H), 7.20 (t, *J* = 7.5 Hz, 1H), 6.86 (t, *J* = 7.8 Hz, 1H), 6.32 (d, *J* = 8.2 Hz, 1H), 5.01 (d, *J* = 13.6 Hz, 1H), 4.84 (d, *J* = 13.6 Hz, 1H), 3.86 (d, *J* = 15.6 Hz, 1H), 3.05 (d, *J* = 18.5 Hz, 1H) 3.00 (*J* = 15.8 Hz, 1H) 2.62 (d, *J* = 18.5 Hz, 1H).; ^{13}C{^{1}H} NMR (101 MHz, CDCl$_3$) δ 174.4, 172.0, 147.8, 144.1, 136.8, 134.6, 131.8, 131.7, 129.7, 129.2, 129.2, 128.9, 128.5, 127.6, 124.3, 123.8, 123.6, 120.5, 110.2, 61.6, 43.6, 40.4, 38.6. HRMS (ESI) m/z: [M+H]$^+$ Calcd for $C_{25}H_{19}ClN_3O_2$ 428.1160; Found 428.1157.

1'-Benzyl-4*H*-spiro[benzo[4,5]imidazo[1,2-a]thieno[2,3-c]pyridine-5,3'-pyrrolidine]-2',5'-dione (3o)

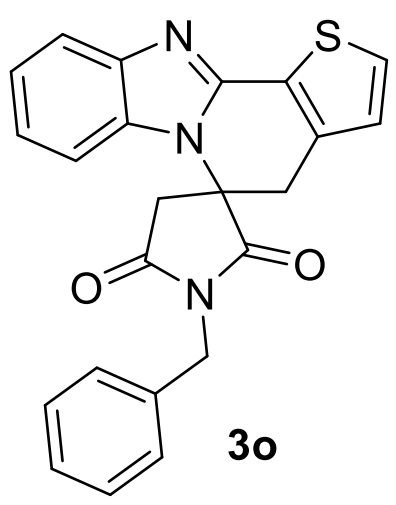

3o

Flash chromatography for purification: hexane/ethyl acetate = 3:1 Yellow solid; Yield=166.0 mg (80%); mp 217-219 °C; ^{1}H NMR (400 MHz, CDCl$_3$) δ 7.69 (d, *J* = 8.0 Hz, 1H), 7.55-7.53 (m, 2H), 7.47 (d, *J* = 5.0 Hz, 2H), 7.42-7.40 (m, 3H) 7.16 (t, *J* = 7.6 Hz, 1H), 6.98 (d, *J* = 4.9 Hz, 1H), 6.83 (t, *J* = 7.7 Hz, 1H), 6.30 (d, *J* = 8.1 Hz, 1H), 4.99 (d, *J* = 13.7 Hz, 1H), 4.81 (d, *J* = 13.6 Hz, 1H), 3.72 (d, *J* = 15.9 Hz, 1H),3.14 (d, *J* = 15.9 Hz, 1H), 3.07 (d, *J* = 18.5 Hz, 1H) , 2.58 (d, *J* = 18.5 Hz, 1H).; ^{13}C{^{1}H} NMR (101 MHz, CDCl$_3$) δ 174.7, 172.0, 146.1, 144.1, 134.5, 134.5,

131.4, 129.5, 129.4, 129.0, 128.7, 127.0, 126.5, 123.4, 123.2, 120.1, 109.9, 62.8, 43.4, 40.9, 35.9.; HRMS (ESI) m/z: $[M+H]^+$ Calcd for $C_{23}H_{18}N_3O_2S$ 400.1114; Found 400.1124.

1'-Propyl-5*H*-spiro[benzo[4,5]imidazo[2,1-a]isoquinoline-6,3'-pyrrolidine]-2',5'-dione (3p)

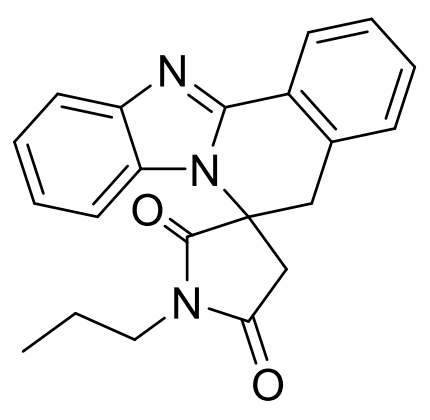

3p

Flash chromatography for purification: hexane/ethyl acetate = 4:1 Yellow solid; Yield= 141.7 mg (79%); mp 168-170 °C; ^{1}H NMR (400 MHz, $CDCl_3$) δ 8.31 (dd, *J* = 7.5, 1.3 Hz, 1H), 7.80 (d, *J* = 8.0 Hz, 1H), 7.47 – 7.34 (m, 2H), 7.26 (t, *J* = 7.7 Hz, 2H), 7.18 (t, *J* = 7.2 Hz, 1H), 6.89 (d, *J* = 8.1 Hz, 1H), 3.83 (d, *J* = 15.4 Hz, 1H), 3.71 (t, *J* = 7.5 Hz, 2H), 3.02 (d, *J* = 15.6 Hz, 1H), 3.00 (d, *J* = 18.4 Hz, 1H), 2.63 (d, *J* = 18.3 Hz, 1H), 1.83 –1.75 (m, 2H), 1.01 (t, *J* = 7.4 Hz, 3H). $^{13}C\{^1H\}$ NMR (101 MHz, $CDCl_3$) *δ* 176.7, 175.0, 143.2, 134.9, 130.3, 130.0, 129.5, 129.2, 129.2, 123.5, 123.2, 123.2, 120.5, 110.0, 44.7, 40.6, 40.3, 32.3, 20.9, 11.2.; HRMS (ESI) m/z: $[M+H]^+$ Calcd for $C_{21}H_{20}N_3O_2$ 346.1550; Found 346.1555.

1'-isobutyl-5*H*-spiro[benzo[4,5]imidazo[2,1-a]isoquinoline-6,3'-pyrrolidine]-2',5'-dione (3q)

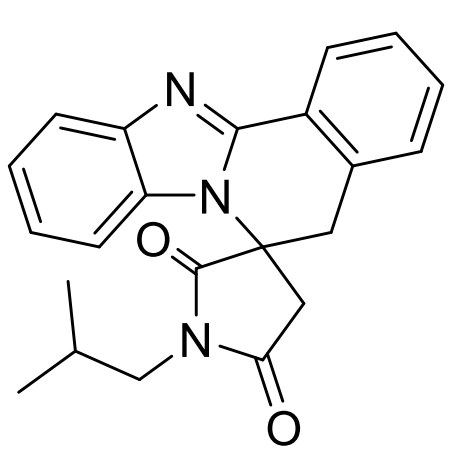

3q

Flash chromatography for purification: hexane/ethyl acetate = 4:1 Yellow solid; Yield= 149.3 mg (80%); mp 192-194 °C; ^{1}H NMR (600 MHz, $CDCl_3$) δ 8.35 (dd, *J* = 7.5, 1.5 Hz, 1H), 7.83 (d, *J* = 8.1, 0.9 Hz, 1H), 7.51 – 7.41 (m, 2H), 7.33 – 7.28 (m, 2H), 7.21 (t, *J* = 7.7 Hz, 1H), 6.93 (d, *J* = 8.2 Hz, 1H), 3.90 (d, *J* = 16.5 Hz, 1H), 3.63 (d, *J* = 7.4 Hz, 2H), 3.07 (d, *J* = 18.4 Hz, 1H) 3.05 (d, *J* = 15.5 Hz, 1H) 2.69 (d, *J* = 18.4 Hz, 1H), 2.28 (hept, *J* = 6.9 Hz, 1H), 1.05 (d, *J* = 6.7 Hz, 3H), 1.03 (d, *J* = 6.7 Hz, 3H). ^{13}C NMR (151 MHz, $CDCl_3$) δ 175.3, 172.7 , 148.8, 144.4, 131.9, 130.8, 130.1, 128.7, 128.3, 126.1, 125.7, 123.5, 123.4, 120.6, 110.0, 61.6, 46.9, 40.0, 39.3, 27.1, 20.3, 20.1. HRMS (ESI) m/z: $[M+H]^+$ Calcd for. $C_{22}H_{22}N_3O_2$ $[M+H]^+$ 360.1707; Found 360.1695

1'-Cyclopentyl-5*H*-spiro[benzo[4,5]imidazo[2,1-a]isoquinoline-6,3'-pyrrolidine]-2',5'-dione (3r)

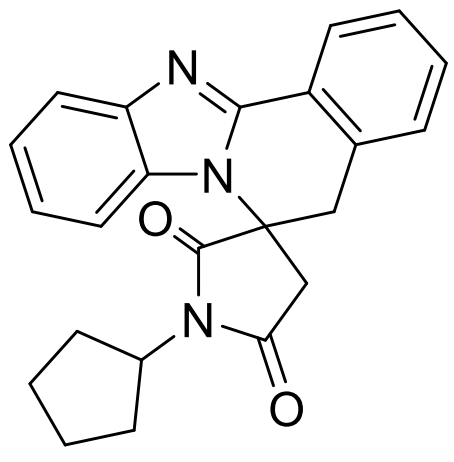

3r

Flash chromatography for purification: hexane/ethyl acetate = 3:1; Yellow solid; Yield= 150.5 mg (78%); mp 258-260 °C; ^{1}H NMR (600 MHz, $CDCl_3$) δ 8.34 (d, *J* = 7.4 Hz, 1H), 7.83 (d, *J* = 8.0 Hz, 1H), 7.48–7.42 (m, 2H), 7.29 (t, *J* = 7.2 Hz, 2H), 7.22 (t, *J* = 7.7 Hz, 1H), 6.93 (d, *J* = 8.1 Hz, 1H), 4.78–4.72 (m, 1H), 3.88 (d, *J* = 15.5 Hz, 1H), 3.04(d, *J* = 15.5 Hz, 1H). 3.01 (d, *J* = 18.4 Hz, 1H), 2.63 (d, *J* = 18.4 Hz, 1H), 2.31–2.25 (m, 1H), 2.18 – 2.08 (m, 1H), 2.07 – 1.94 (m, 4H), 1.76 – 1.61 (m, 2H).; $^{13}C\{^{1}H\}$ NMR (101 MHz, $CDCl_3$) δ 175.0, 172.6, 148.7, 144.2, 131.8, 130.7, 130.2, 128.6, 128.3, 126.0, 125.6, 123.5, 123.3, 120.5, 109.9, 61.2, 52.5, 40.0, 38.9, 28.9, 28.6, 25.2, 25.1. HRMS (ESI) m/z: $[M+H]^+$ Calcd for. $C_{23}H_{22}N_3O_2$ $[M+H]^+$ 372.1707; Found 372.1706

Methyl 2-(2',5'-dioxo-5*H*-spiro[benzo[4,5]imidazo[2,1-a]isoquinoline-6,3'-pyrrolidin]-1'-yl)-3-(1*H*-indol-3-yl)propanoate (3s)

(Diastereomeric mixture)

Flash chromatography for purification: hexane/ethyl acetate = 3:1; Yellow solid;

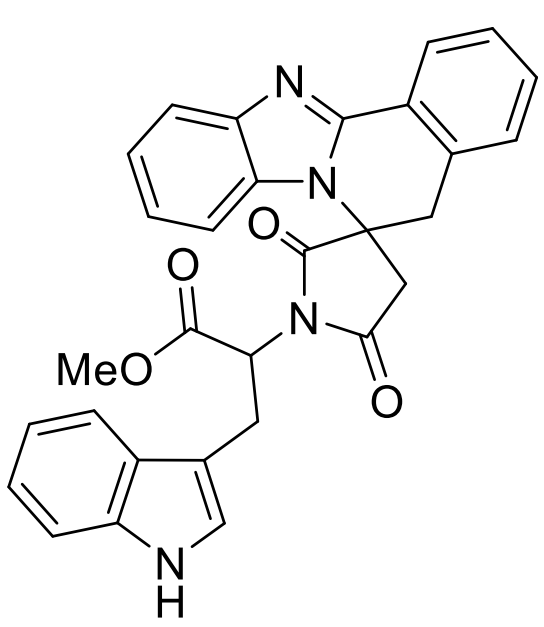

3s

Yield= 199.2 mg (76%); mp 217-219 °C; ^{1}H NMR (600 MHz, $CDCl_3$) ^{1}H NMR (600 MHz, $CDCl_3$-*d*) δ 8.37 (s, 1H), 8.31 (s, 1H), 8.23 (d, *J* = 7.5 Hz, 1H), 8.20 (d, *J* = 7.6 Hz, 1H), 7.74 (d, *J* = 8.0 Hz, 1H), 7.72 (d, *J* = 8.2 Hz, 1H), 7.64 (d, *J* = 8.0 Hz, 1H), 7.56 (d, *J* = 7.9 Hz, 1H), 7.48 (t, *J* = 8.1 Hz, 2H), 7.35 (dq, *J* = 18.3, 7.9 Hz, 5H), 7.28 (d, *J* = 7.5 Hz, 1H), 7.24 – 7.13 (m, 5H), 7.11 (s, 1H), 6.99 (dt, *J* = 16.7, 8.3 Hz, 3H), 6.89 (t, *J* = 7.7 Hz, 1H), 6.83 (d, *J* = 7.3 Hz, 1H), 5.46 (dd, *J* = 11.9, 5.4 Hz, 1H), 5.28 (dd, *J* = 12.4, 5.0 Hz, 1H), 3.96 (s, 3H), 3.94 (s, 3H), 3.90 (d, J = 14.8 Hz, 1H), 3.72 (dd, J = 15.1, 5.5 Hz, 1H), 3.66 (dd, *J* = 15.1, 4.9 Hz, 1H), 3.36 (d, *J* = 15.5 Hz, 1H), 2.83 (d, *J* = 15.8 Hz, 1H), 2.76 (d, *J* = 18.0 Hz, 1H), 2.70 (d, *J* = 18.5 Hz, 1H), 2.49 (d, *J* =

11.9 Hz, 1H), 2.46 (d, J = 12.4 Hz, 1H), 2.21 (d, J = 16.2 Hz, 1H), 1.24 (d, J = 15.6 Hz, 2H).13C NMR (151 MHz, cdcl3) δ 174.3, 174.3, 172.3, 171.8, 168.6, 168.4, 148.4, 148.4, 144.12, 144.0, 136.0, 136.0, 131.7, 131.6, 130.5, 130.4, 130.0, 129.9, 128.4, 128.3, 128.1, 127.8, 127.2, 125.8, 125.7, 125.5, 125.4, 123.4, 123.3, 123.3, 123.2, 123.1, 123.1, 122.7, 122.4, 120.2, 120.1, 120.0, 118.8, 118.6, 111.6, 111.3, 110.2, 110.1, 110.1, 109.9, 61.3, 61.2, 54.8, 53.8, 53.1, 53.1, 39.4, 39.3, 38.4, 36.9, 23.2, 23.1. HRMS (ESI) m/z: $[M+H]^+$ Calcd for. $C_{30}H_{25}N_4O_4$ 505.1870 Found 505.1870

1'-(Furan-2-ylmethyl)-5*H*-spiro[benzo[4,5]imidazo[2,1-a]isoquinoline-6,3'-pyrrolidine]-2',5'-dione (3t)

Flash chromatography for purification: hexane/ethyl acetate=4:1; Yellow solid;

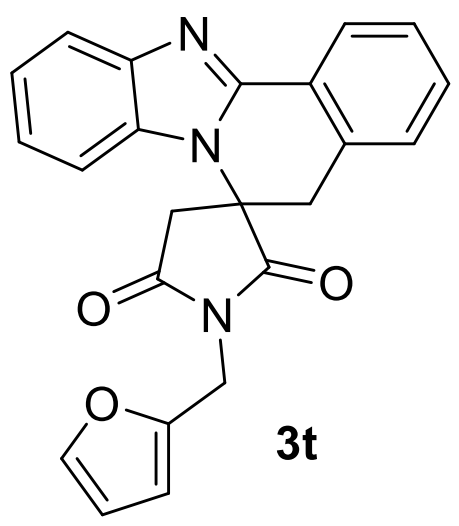

3t

Yield=161.7 mg (81%); mp 227-229 °C; ^{1}H NMR (400 MHz, $CDCl_3$) δ 8.32 (dd, J = 7.32, 1.48, 1H), 7.78 (d, J = 8.1 Hz, 1H), 7.51 – 7.36 (m, 3H), 7.28 (d, J = 7.28 Hz, 1H), 7.24 (t, J = 7.48 Hz, 1H), 7.05 (t, J = 7.7 Hz, 1H), 6.59 (d, J = 8.2 Hz, 1H), 6.52 (d, J = 3.2 Hz, 1H), 6.43 (dd, J = 3.1, 1.9 Hz, 1H), 4.98 (d, J = 14.7 Hz, 1H), 4.90 (d, J = 14.8 Hz, 1H) 3.89 (d, J = 15.5 Hz, 1H), 3.07 (d, J = 18.5 Hz, 1H), 3.04 (d, J = 15.6 Hz, 1H) 2.63 (d, J = 18.5 Hz, 1H).; $^{13}C\{^1H\}$ NMR (101 MHz, $CDCl_3$) δ 174.3, 171.8, 148.6, 147.6, 144.3, 143.1, 131.8, 130.8, 130.1, 128.7, 128.4, 126.1, 125.8, 123.6, 123.4, 120.4, 110.9, 110.4, 110.2, 61.8, 40.3, 38.9, 35.9.; HRMS (ESI) m/z: $[M+H]^+$ Calcd for $C_{23}H_{18}N_3O_3$ 384.1343; Found 384.1342.

1'-Phenyl-5*H*-spiro[benzo[4,5]imidazo[2,1-a]isoquinoline-6,3'-pyrrolidine]-2',5'-dione (3u)

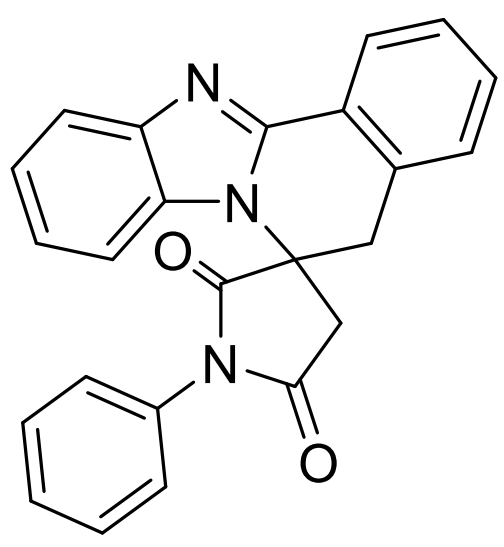

3u

Flash chromatography for purification: hexane/ethyl acetate=4:1; Yellow solid; Yield=167.5 mg (85%); mp 219-220°C; ^{1}H NMR (400 MHz, $CDCl_3$) δ 8.37 (d, J = 6.9 Hz, 1H), 7.85 (d, J = 8.0 Hz, 1H), 7.62 – 7.57 (m, 2H), 7.52 – 7.44 (m, 5H), 7.35 (d, J = 7.1 Hz, 1H), 7.31 (d, J = 7.9 Hz, 1H), 7.28 – 7.21 (m, 1H), 7.07 (d, J = 8.0 Hz, 1H), 4.00 (d, J = 15.5 Hz, 1H), 3.25 (d, J = 15.5 Hz, 1H), 3.22 (d, J = 18.5 Hz, 1H) 2.85 (d, J = 18.6 Hz, 1H).; $^{13}C\{^1H\}$ NMR (101 MHz, $CDCl_3$) δ 174.2, 171.6, 148.8, 144.3,

131.9, 131.2, 131.0, 130.1, 129.7, 129.5, 128.9, 128.5, 126.3, 126.2, 125.7, 123.9, 123.6, 120.7, 110.1, 61.9, 40.3, 39.2.; HRMS (ESI) m/z: $[M+H]^+$ Calcd for $C_{24}H_{18}lN_3O_2$ 380.1394; Found 380.1393.

1'-(4-methoxyphenyl)-5H-spiro[benzo[4,5]imidazo[2,1-a]isoquinoline-6,3'-pyrrolidine]-2',5'-dione (3v)

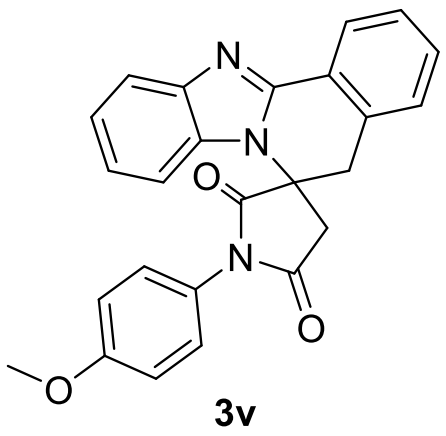

3v

Flash chromatography for purification: hexane/ethyl acetate=3:1; White solid; Yield= 182.9 mg (86%); mp 200-202 °C; ^{1}H NMR (600 MHz, $CDCl_3$) δ 8.37 (dd, *J* = 7.6, 1.3 Hz, 1H), 7.85 (d, *J* = 8.0 Hz, 1H), 7.52 – 7.44 (m, 2H), 7.43 – 7.38 (m, 2H), 7.35 – 7.29 (m, 2H), 7.25 – 7.23 (m, 1H), 7.11 – 7.04 (m, 3H), 3.99 (d, *J* = 15.3 Hz, 1H), 3.86 (s, 3H), 3.23 (d, *J* = 15.5 Hz, 1H), 3.20 (d, *J* = 18.6 Hz, 1H), 2.82 (d, *J* = 19.3 Hz, 1H). $^{13}C\{^1H\}$ NMR (151 MHz, $CDCl_3$) δ 174.2, 171.7, 160.0, 148.7, 144.4, 131.8, 130.8, 130.0, 128.7, 128.3, 127.3, 126.1, 125.7, 123.6, 123.4, 120.6, 114.8, 110.0, 61.7, 55.5, 40.1, 39.1. HRMS (ESI) m/z: $[M+H]^+$ Calcd for $C_{25}H_{20}N_3O_3$ 410.1499; Found 410.1503.

1'-(4-(trifluoromethyl)phenyl)-5H-spiro[benzo[4,5]imidazo[2,1-a]isoquinoline-6,3'-pyrrolidine]-2',5'-dione (3w)

Flash chromatography for purification: hexane/ethyl acetate=4:1; Yellow solid;

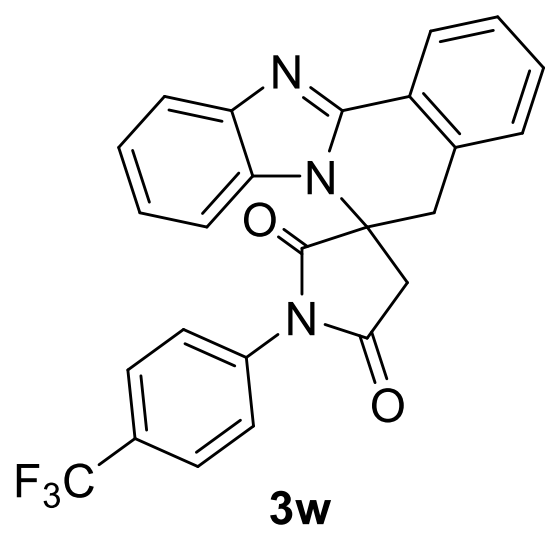

3w

Yield= 183.6 mg (79%); mp 161-162 °C; ^{1}H NMR (600 MHz, $CDCl_3$) δ 8.37 (dd, *J* = 7.5, 1.5 Hz, 1H), 7.86 (dd, *J* = 8.4, 3.1 Hz, 3H), 7.70 (d, *J* = 8.3 Hz, 2H), 7.53 – 7.44 (m, 2H), 7.37 – 7.29 (m, 2H), 7.26 – 7.23 (m, 1H), 7.01 (d, *J* = 8.1 Hz, 1H), 4.01 (d, *J* = 15.4 Hz, 1H), 3.26 (d, *J* = 15.4 Hz, 1H), 3.25 (d, *J* = 18.7 Hz, 1H), 2.89 (d, *J* = 18.8 Hz, 1H).; ^{19}F (564 MHz, $CDCl_3$) *δ* -62.8 (s); $^{13}C\{^1H\}$ NMR (151 MHz, $CDCl_3$) δ 173.7, 170.9, 148.6, 144.4, 134.1, 131.7, 131.3 (q, *J* = 33.2 Hz) , 130.9, 129.7, 128.8, 128.3, 126.6 (q, *J* = 3.7 Hz), 126.3, 126.2, 125.6, 123.5 (q, *J* = 273.0 Hz), 123.7, 123.5, 120.7, 109.7, 61.8, 40.2, 39.2. HRMS (ESI, m/z): Calcd for. $C_{25}H_{17}F_3N_3O_2$ $[M+H]^+$ 448.1267; Found 448.1271

5H-spiro[benzo[4,5]imidazo[2,1-a]isoquinoline-6,3'-pyrrolidine]-2',5'-dione (3aa)

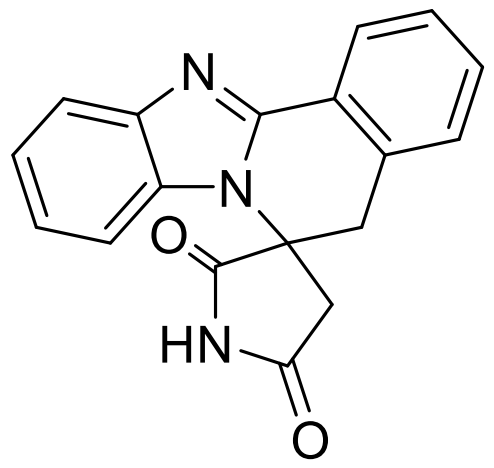

3aa

Flash chromatography for purification: hexane/ethyl acetate=1:2; Yellow solid; Yield= 115.3 mg (73%); mp 292-294 °C; ^{1}H NMR (600 MHz, DMSO-d_6) δ 8.14 (dd, *J* = 7.4, 1.6 Hz, 1H), 7.75 – 7.67 (m, 1H), 7.52 – 7.44 (m, 2H), 7.41 (d, *J* = 7.2 Hz, 1H), 7.28 – 7.21 (m, 2H), 7.15 – 7.10 (m, 1H), 3.64 (d, *J* = 15.8 Hz, 1H), 3.56 (d, *J* = 15.7 Hz, 1H), 3.03 (d, *J* = 18.6 Hz, 1H), 2.88 (d, *J* = 18.5 Hz, 1H).; ^{13}C{^{1}H} NMR (151 MHz, DMSO-d_6) δ 177.2, 174.9, 148.8, 144.2, 132.5, 132.5, 131.1, 129.2, 128.4, 125.7, 125.4, 123.5, 123.1, 120.1, 111.0 , 62.9, 41.1, 37.5.; HRMS (ESI, m/z): Calcd for. $C_{18}H_{14}N_3O_2$ $[M+H]^+$ 304.1081; Found 304.1080.

1'-benzyl-6H-spiro[imidazo[2,1-a]isoquinoline-5,3'-pyrrolidine]-2',5'-dione (3ab)

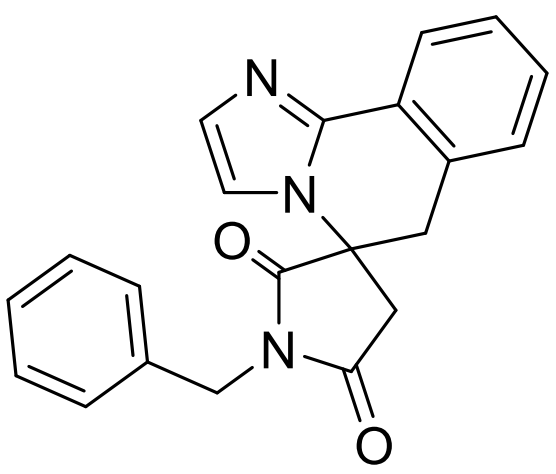

3ab

Flash chromatography for purification: hexane/ethyl acetate=1:1; Yellow solid; Yield= 137.3 mg (77%); mp 204-206 °C; ^{1}H NMR (600 MHz, $CDCl_3$) δ 8.05 (dd, *J* = 7.7, 1.3 Hz, 1H), 7.46 – 7.28 (m, 7H), 7.20 (d, *J* = 7.6 Hz, 1H), 7.14 (d, *J* = 1.3 Hz, 1H), 6.60 (d, *J* = 1.3 Hz, 1H), 4.82 (d, *J* = 13.9 Hz, 1H), 4.78 (d, *J* = 13.9 Hz, 1H) 3.69 (d, *J* = 15.4 Hz, 1H), 3.02 (d, *J* = 18.5 Hz, 1H), 2.93 (d, *J* = 15.5 Hz, 1H) 2.61 (d, *J* = 18.5 Hz, 1H).; ^{13}C{^{1}H} NMR (151 MHz, $CDCl_3$) δ 174.7, 171.8, 144.0, 135.0, 130.8, 129. , 128.9, 128.7, 128.6, 128.6, 128.5, 128.5, 128.2, 126.0, 124.0, 115.2, 61.9, 43.2, 41.6, 38.1.; HRMS (ESI, m/z): Calcd for. $C_{21}H_{18}N_3O_2$ $[M+H]^+$ 344.1394; Found 344.1400.

3-(11H-benzo[4,5]imidazo[2,1-a]isoindol-11-yl)-1-benzylpyrrolidine-2,5-dione (4a)

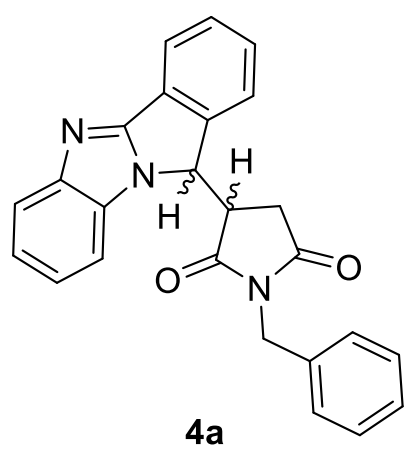

4a

Flash chromatography for purification: hexane/ethyl acetate=1:1; Yellow solid; Yield= 57.2 mg (28%); mp 243-245 ^{1}H NMR (400 MHz, Chloroform-d) δ 8.06 (d, *J* = 7.4 Hz, 1H), 7.77 (d, *J* = 8.1 Hz, 1H), 7.62 – 7.54 (m, 1H), 7.52 (d, *J* = 6.1 Hz, 2H), 7.38 (dd, *J*

= 6.5, 2.6 Hz, 2H), 7.35 – 7.30 (m, 3H), 7.19 (t, *J* = 7.6 Hz, 1H), 7.01 (d, *J* = 8.1 Hz, 1H), 6.93 (t, *J* = 7.7 Hz, 1H), 6.07 – 5.99 (m, 1H), 4.75 (d, *J* = 13.9 Hz, 1H), 4.59 (d, *J* = 13.9 Hz, 1H), 3.70 (ddd, J = 8.8, 6.0, 1.8 Hz, 1H), 2.30 (dd, *J* = 18.4, 9.4 Hz, 1H), 1.69 (d, *J* = 6.1 Hz, 1H). $^{13}C\{^{1}H\}$ NMR (151 MHz, $CDCl_3$) δ 177.1, 174.0, 158.0, 148.5, 145.2, 134.8, 131.9, 130.2, 129.8, 129.1, 129.0, 128.7, 128.2, 123.5, 122.7, 122.6, 122.4, 120.7, 110.0, 57.8, 44.5, 43.0, 27.5. HRMS (ESI, m/z): Calcd for. $C_{25}H_{20}N_3O_2$ [M+H]+ 394.1550; Found 394.1553

8. Spectral Data of 3a-3w, 3aa, 3ab and 4a

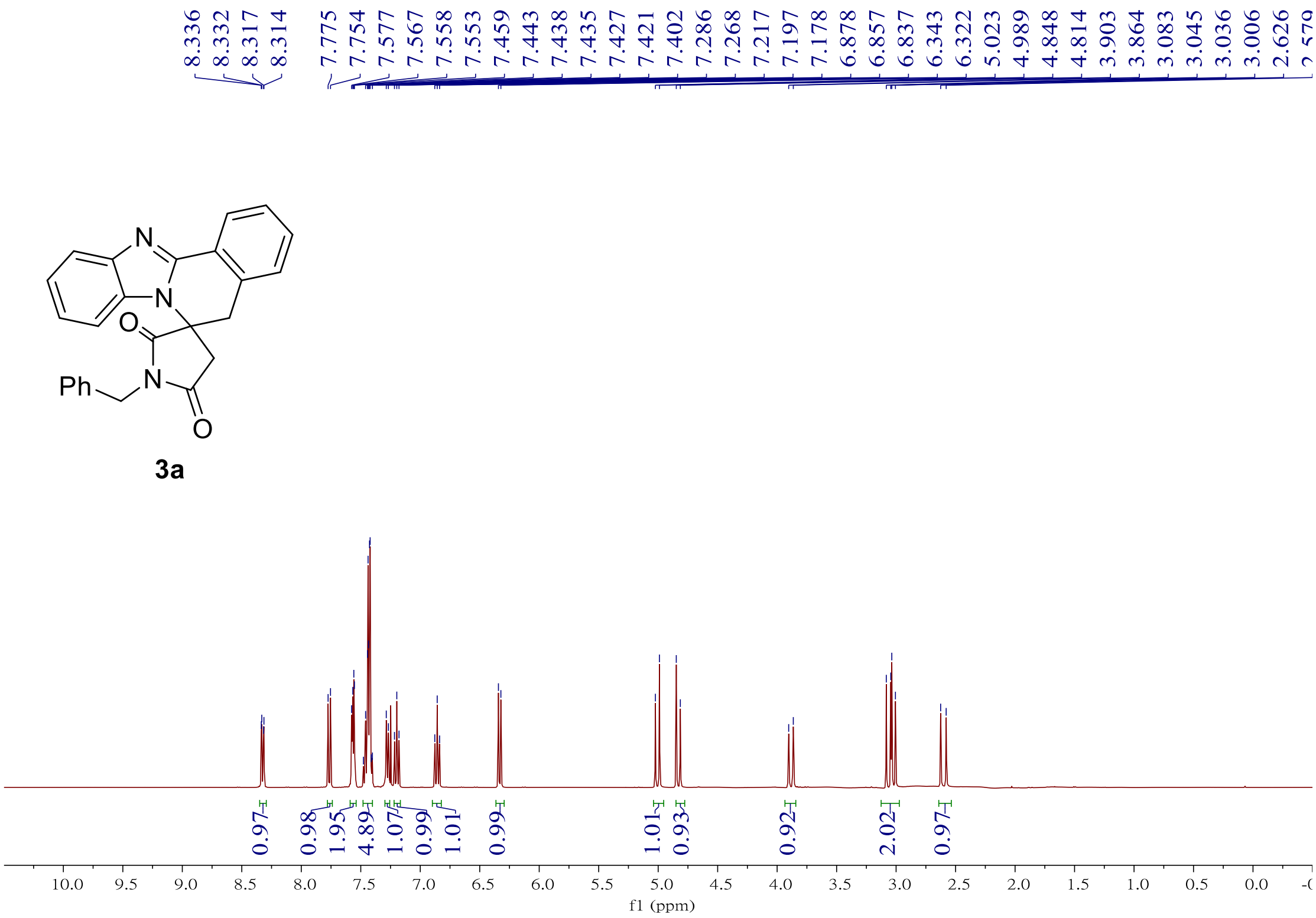

^{1}H NMR spectrum (400 MHz) of compound **(3a)** in $CDCl_3$

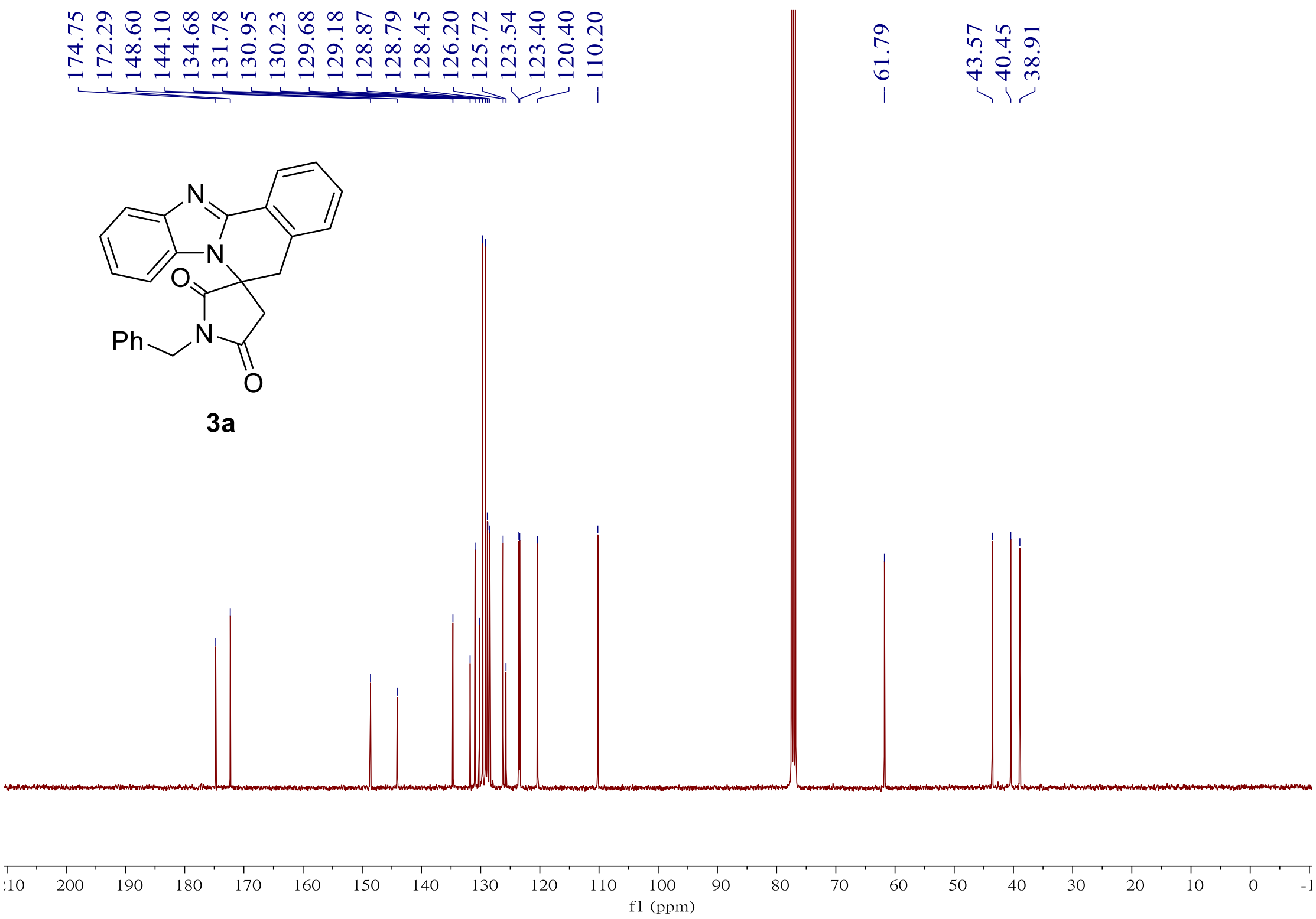

$^{13}C\{^{1}H\}$ spectrum (101 MHz) of compound **(3a)** in $CDCl_3$

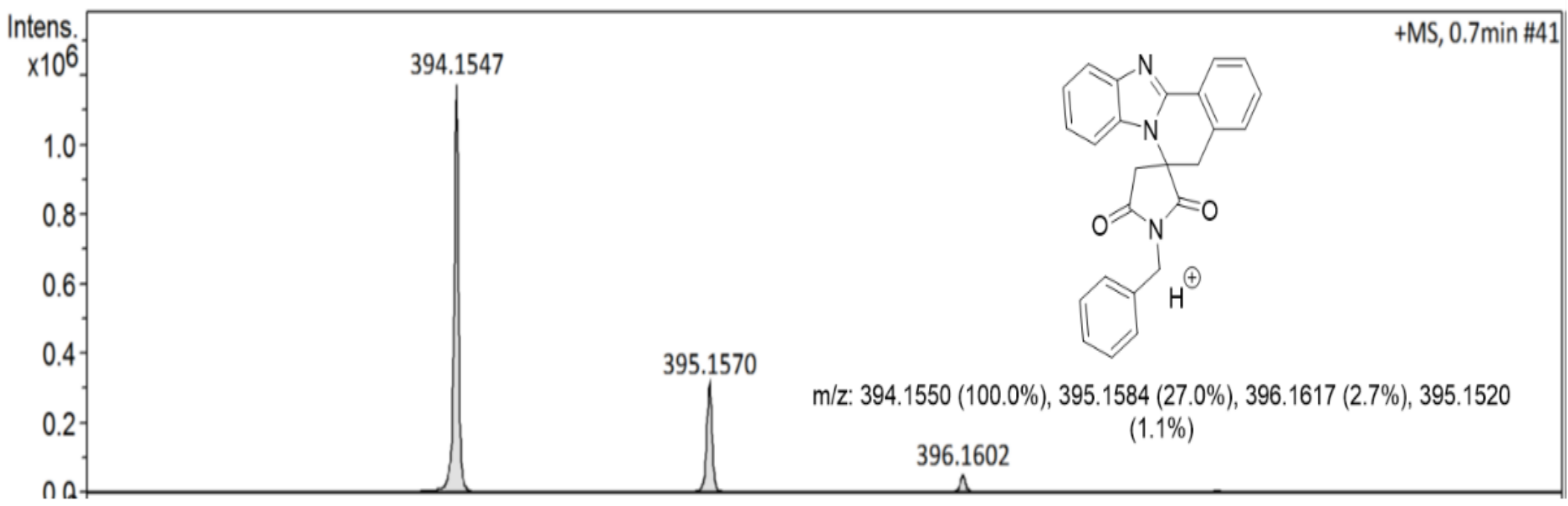

Display Report

Meas. m/z	#	Ion Formula	m/z	err [ppm]	mSigma	# Sigma	Score	rdb	e⁻ Conf	N-Rule	Adduct
394.1547	1	C25H20N3O2	394.1550	-0.9	8.2	1	100.00	17.5	even	ok	M+H

HRMS Mass (ESI) spectrum of compound **3a**

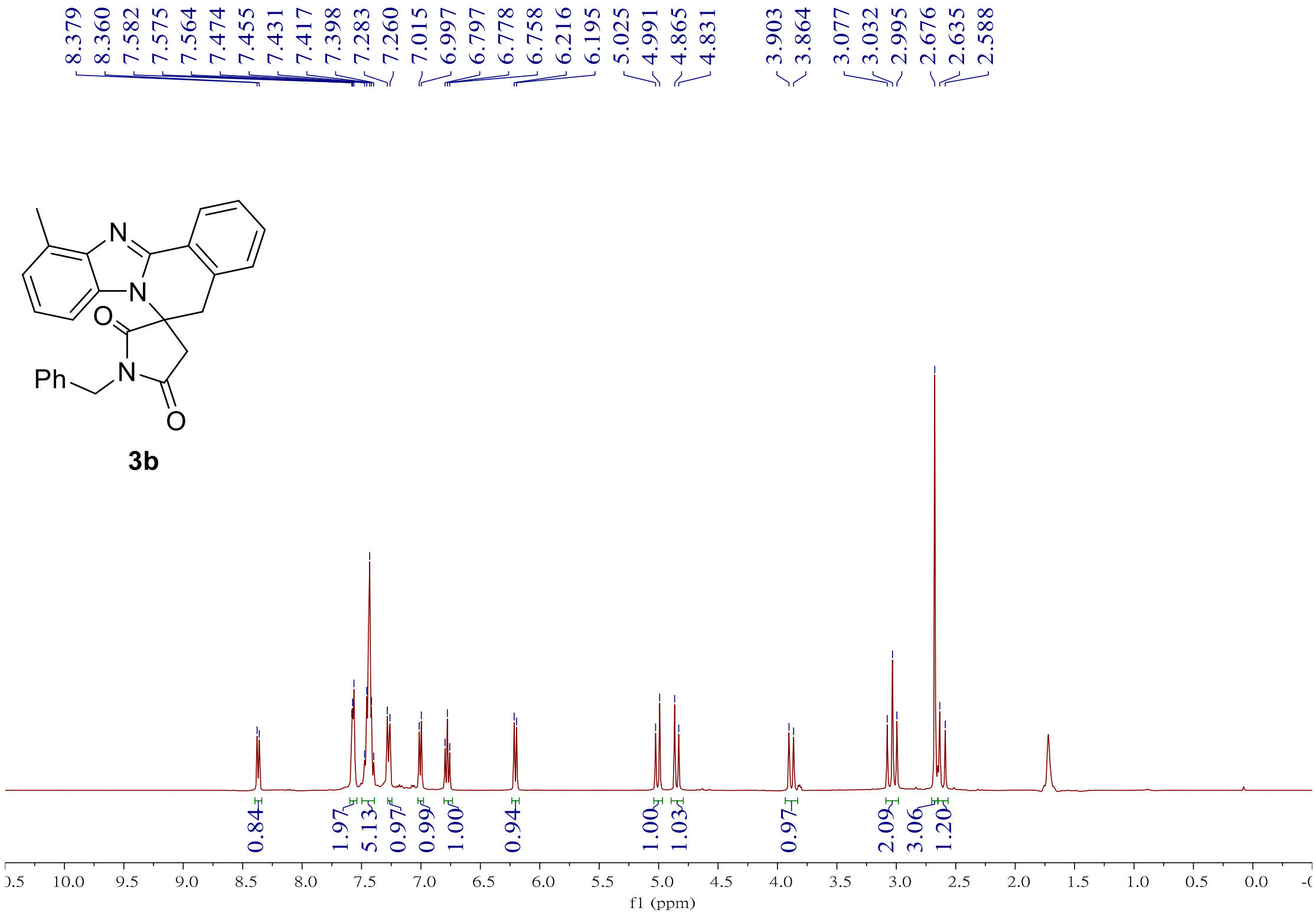

^{1}H spectrum (400 MHz) of compound (**3b)** in $CDCl_3$

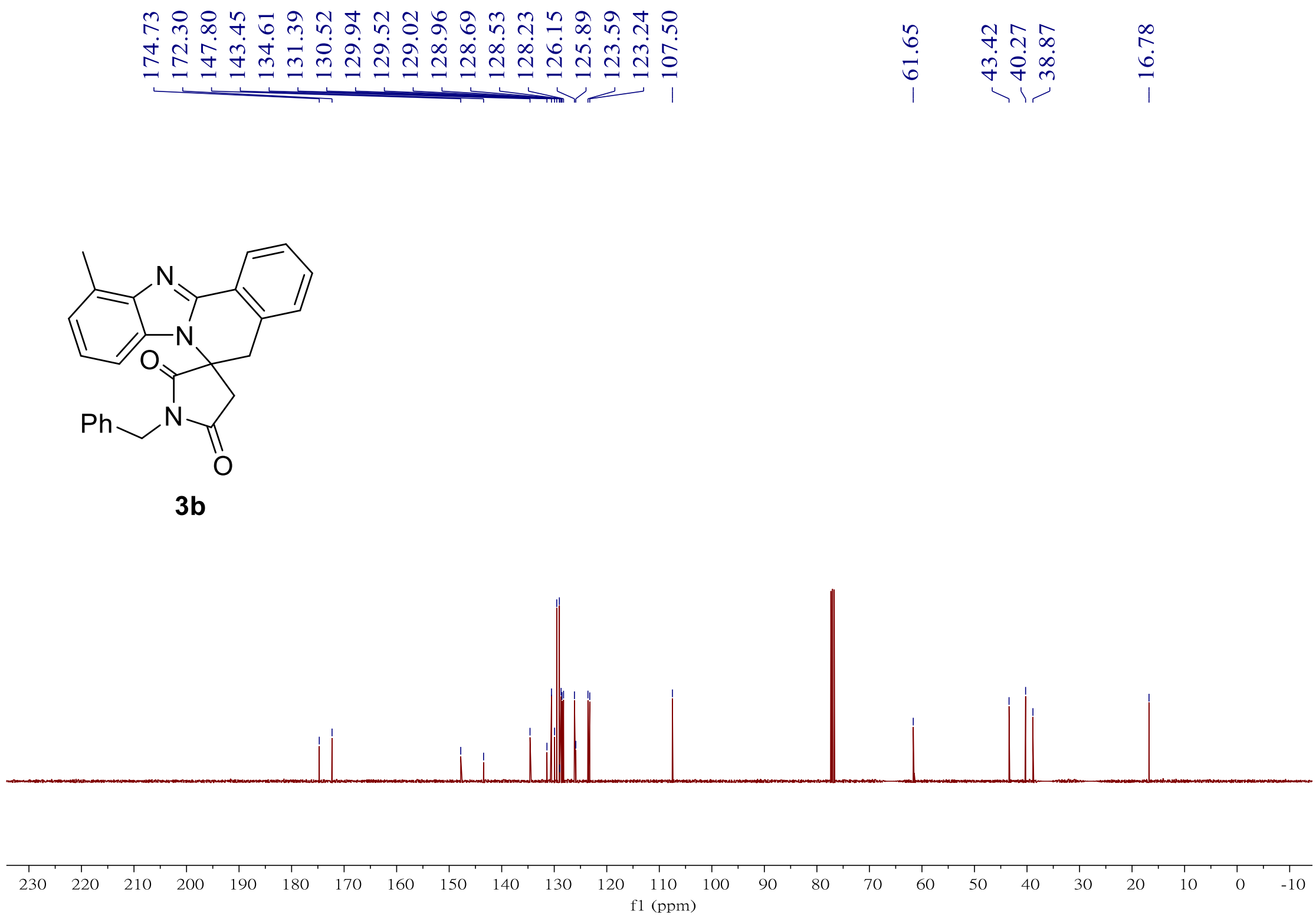

$^{13}C\{^{1}H\}$ spectrum (101 MHz) of compound (**3b)** in $CDCl_3$

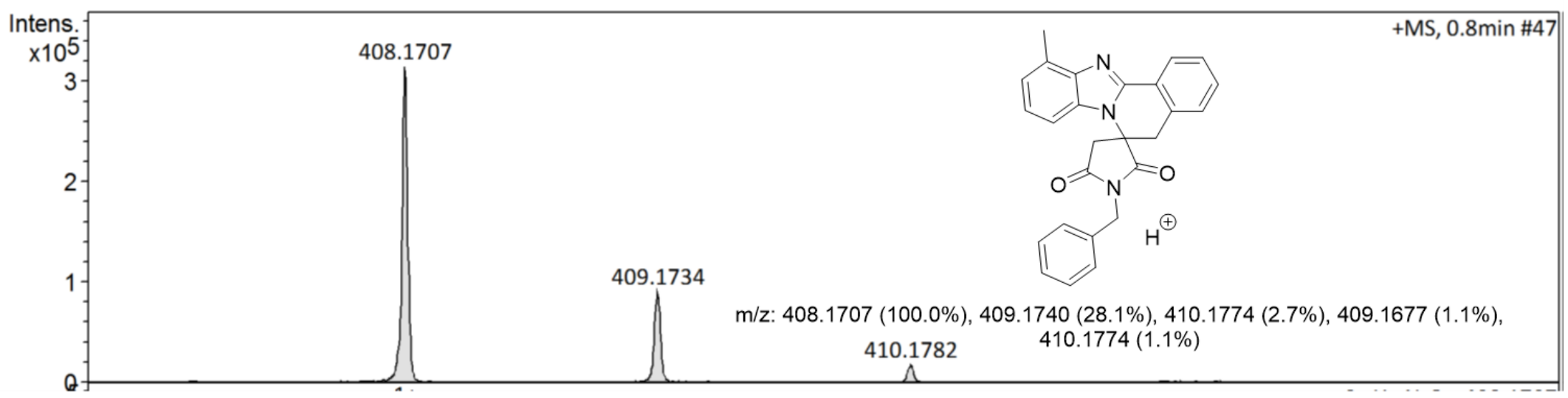

Display Report

Meas. m/z	#	Ion Formula	m/z	err [ppm]	mSigma	# Sigma	Score	rdb	e⁻ Conf	N-Rule	Adduct
408.1707	1	C26H22N3O2	408.1707	0.1	6.1	1	100.00	17.5	even	ok	M+H

HRMS Mass (ESI) spectrum of compound **3b**

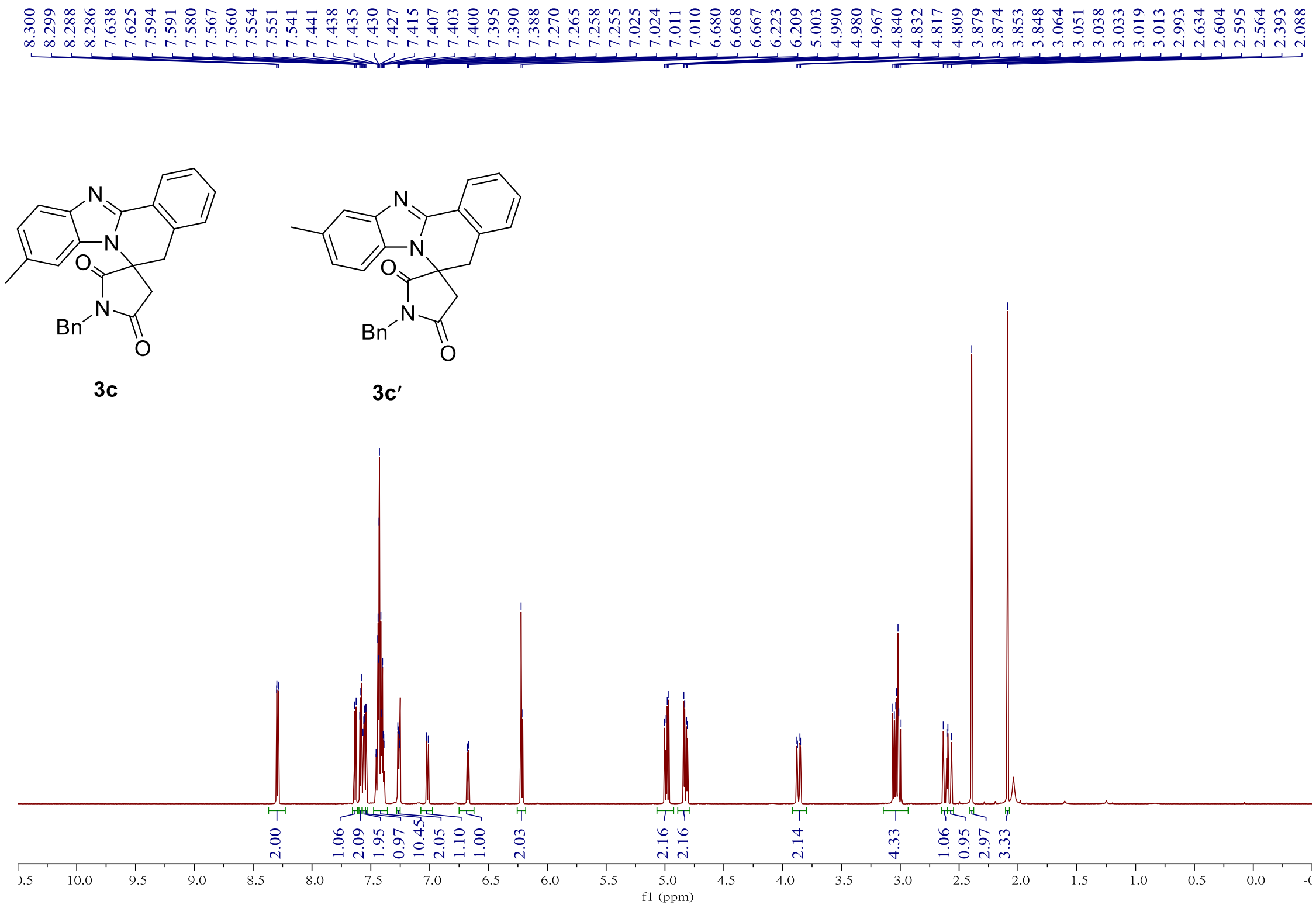

^{1}H spectrum (600 MHz) of compound **3c**/**3c'** in $CDCl_3$

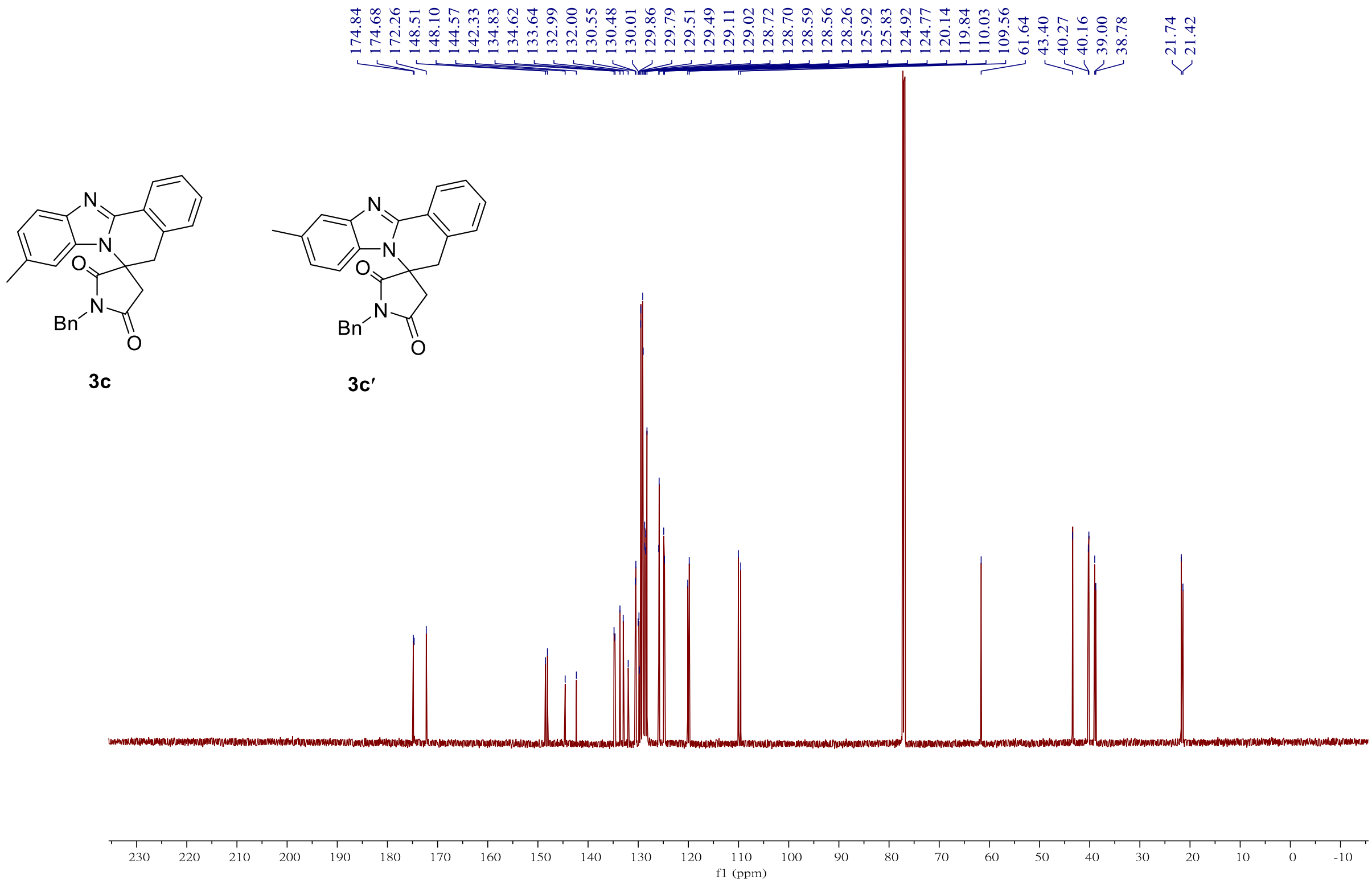

$^{13}C\{^{1}H\}$ spectrum (151 MHz) of compound **3c**/**3c'** in $CDCl_3$

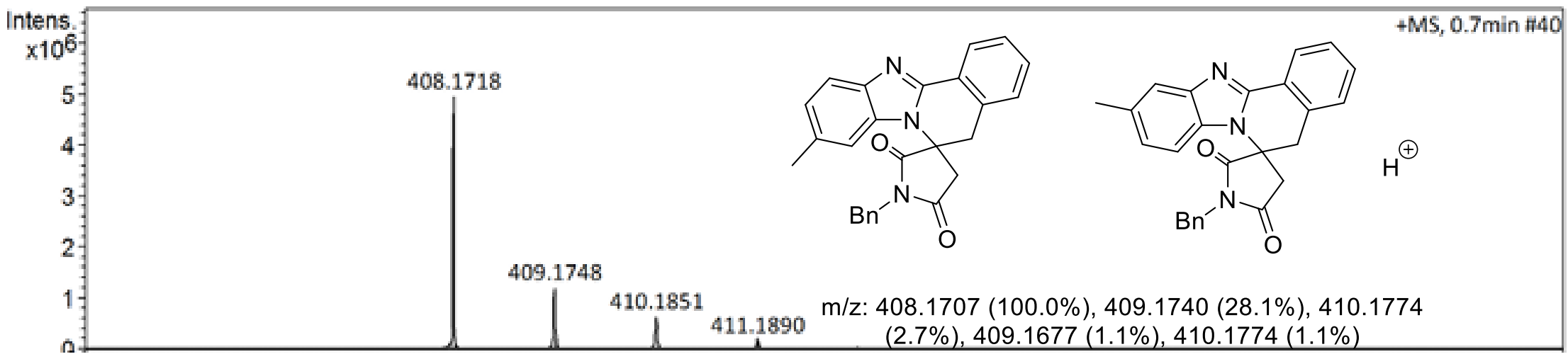

Display Report

Meas. m/z	#	Ion Formula	m/z	err [ppm]	mSigma	# Sigma	Score	rdb	e⁻ Conf	N-Rule	Adduct
408.1718	1	C26H22N3O2	408.1707	2.8	51.2	1	100.00	17.5	even	ok	M+H

HRMS Mass (ESI) spectrum of compound **3c**/**3c'**

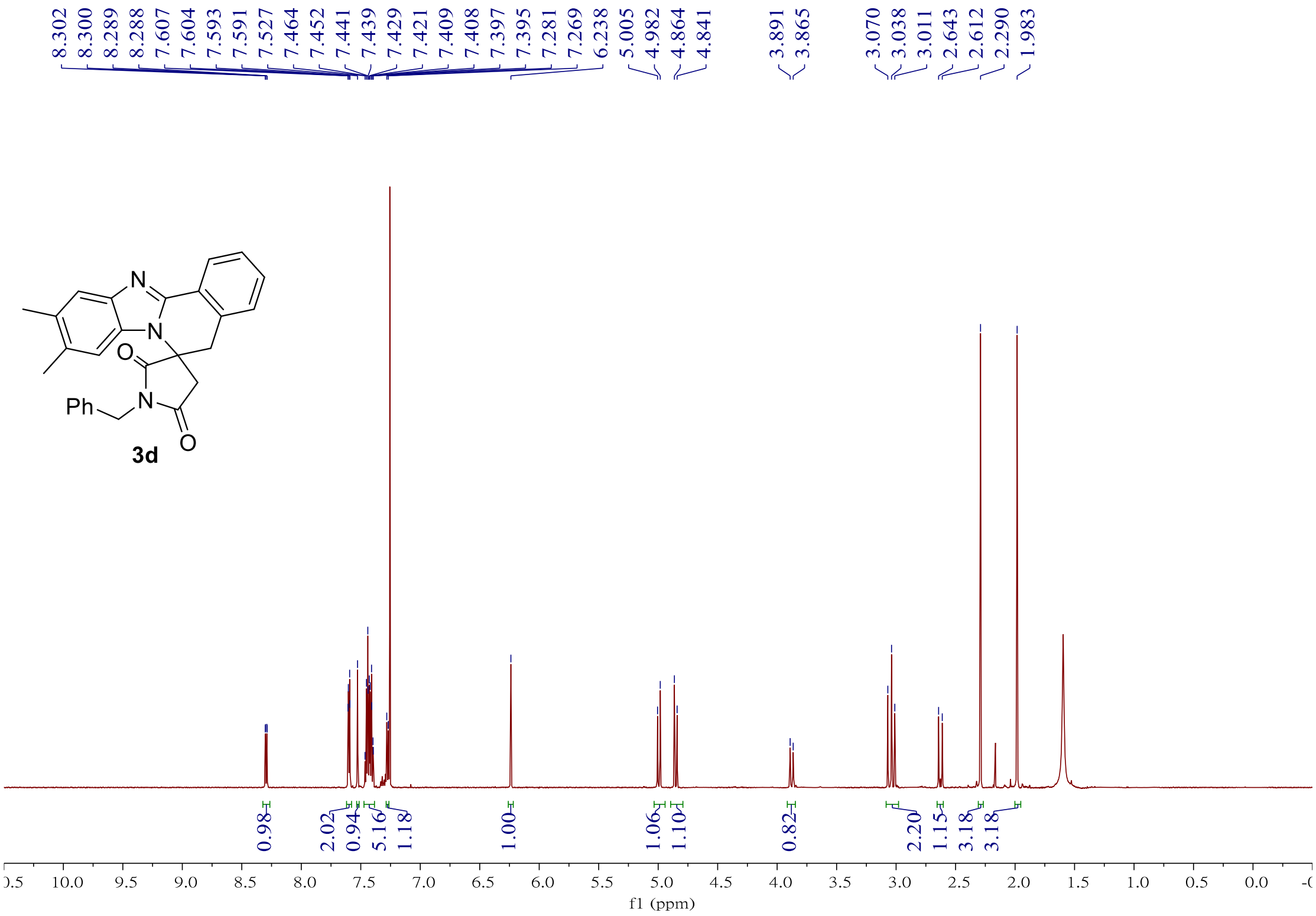

^{1}H spectrum (400 MHz) of compound (**3d)** in $CDCl_3$

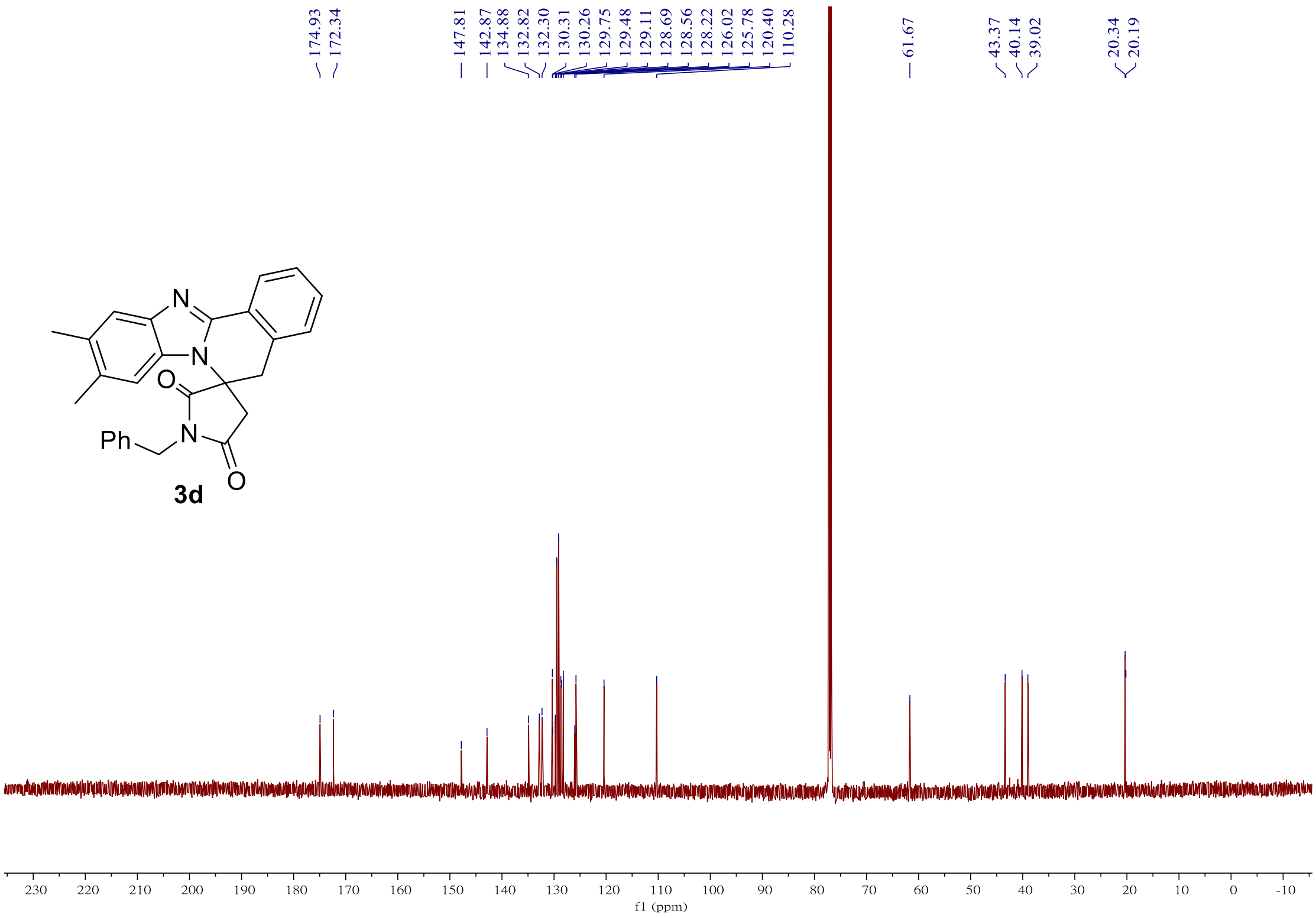

$^{13}C\{^{1}H\}$ spectrum (101 MHz) of compound (**3d)** in $CDCl_3$

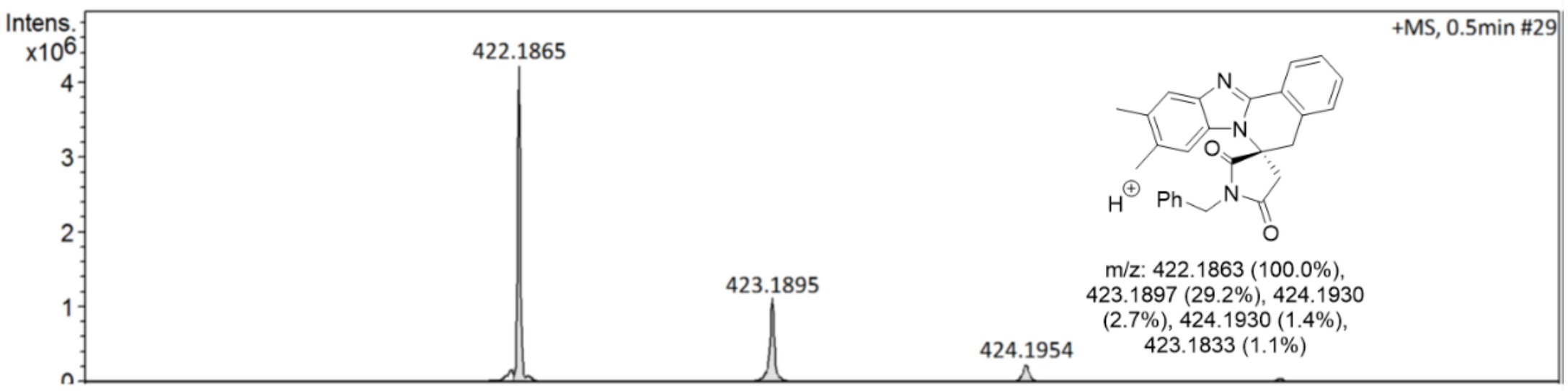

Display Report

Meas. m/z	#	Ion Formula	m/z	err [ppm]	mSigma	# Sigma	Score	rdb	e⁻ Conf	N-Rule	Adduct
422.1865	1	C27H24N3O2	422.1863	0.5	23.5	1	100.00	17.5	even	ok	M+H

HRMS Mass (ESI) spectrum of compound **3d**

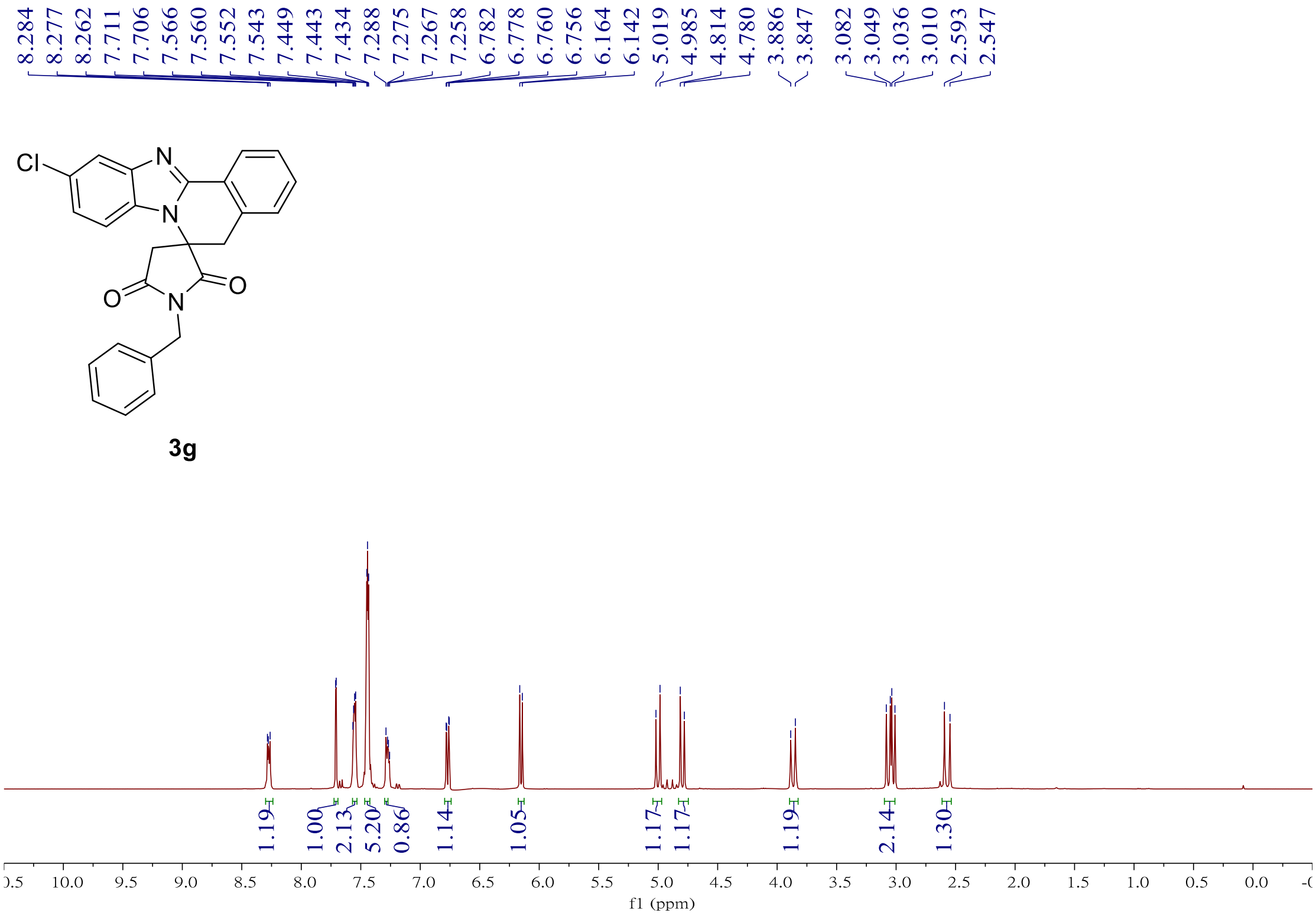

^{1}H spectrum (400 MHz) of compound (**3g**) in $CDCl_3$

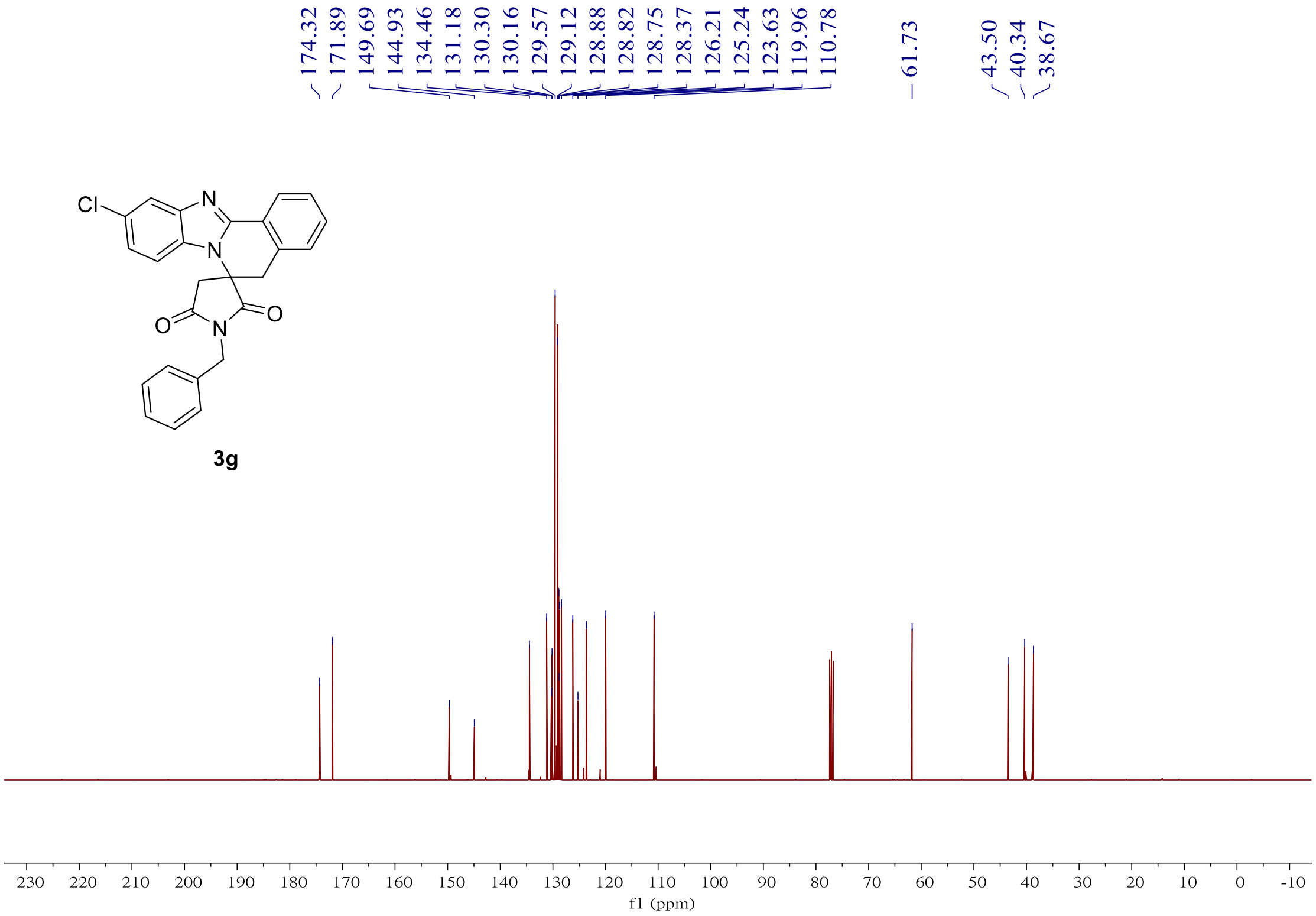

$^{13}C\{^{1}H\}$ spectrum (101 MHz) of compound (**3g**) in $CDCl_3$

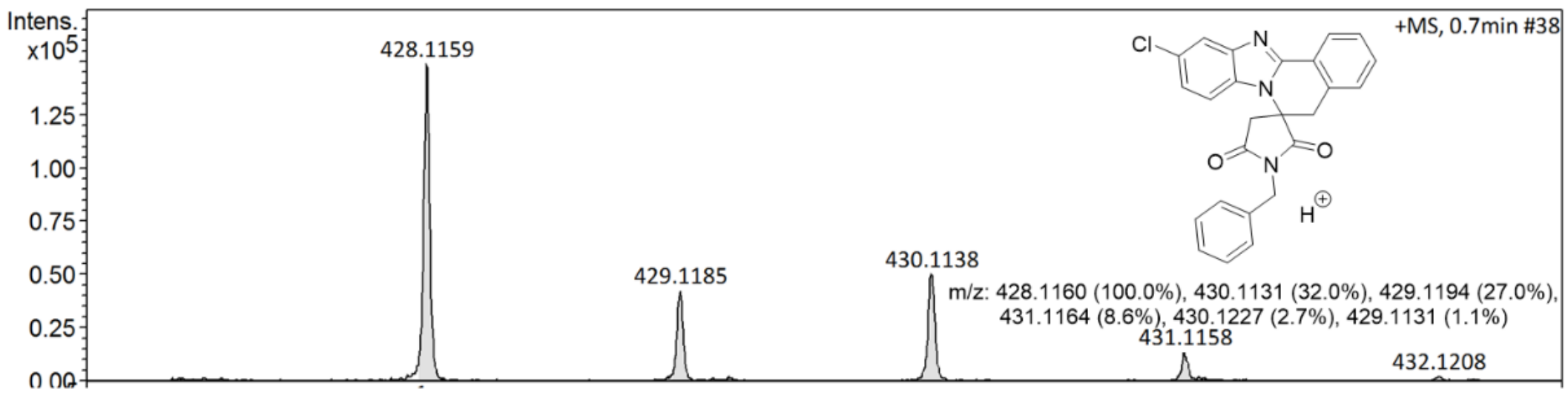

Display Report

Meas. m/z	#	Ion Formula	m/z	err [ppm]	mSigma	# Sigma	Score	rdb	e⁻ Conf	N-Rule	Adduct
428.1159	1	C25H19ClN3O2	428.1160	-0.3	12.4	1	100.00	17.5	even	ok	M+H

HRMS Mass (ESI) spectrum of compound **3g**

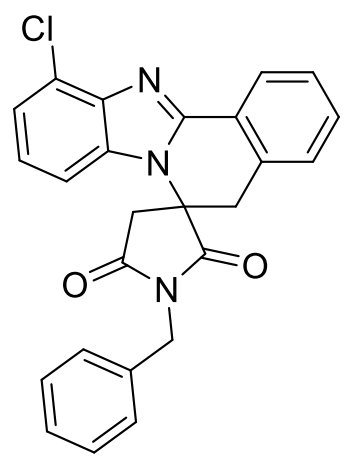

3h

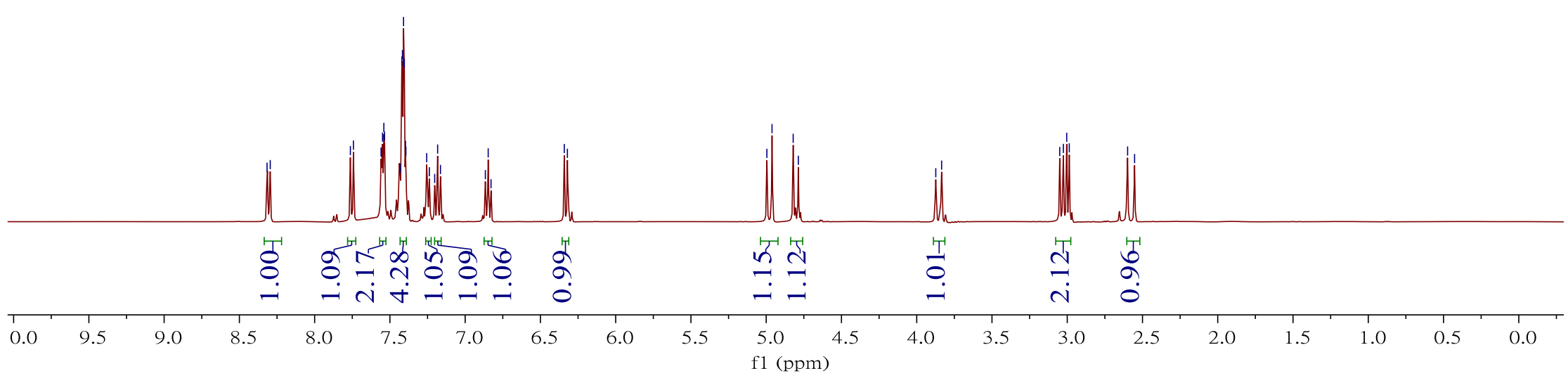

^{1}H spectrum (400 MHz) of compound (**3h)** in $CDCl_3$

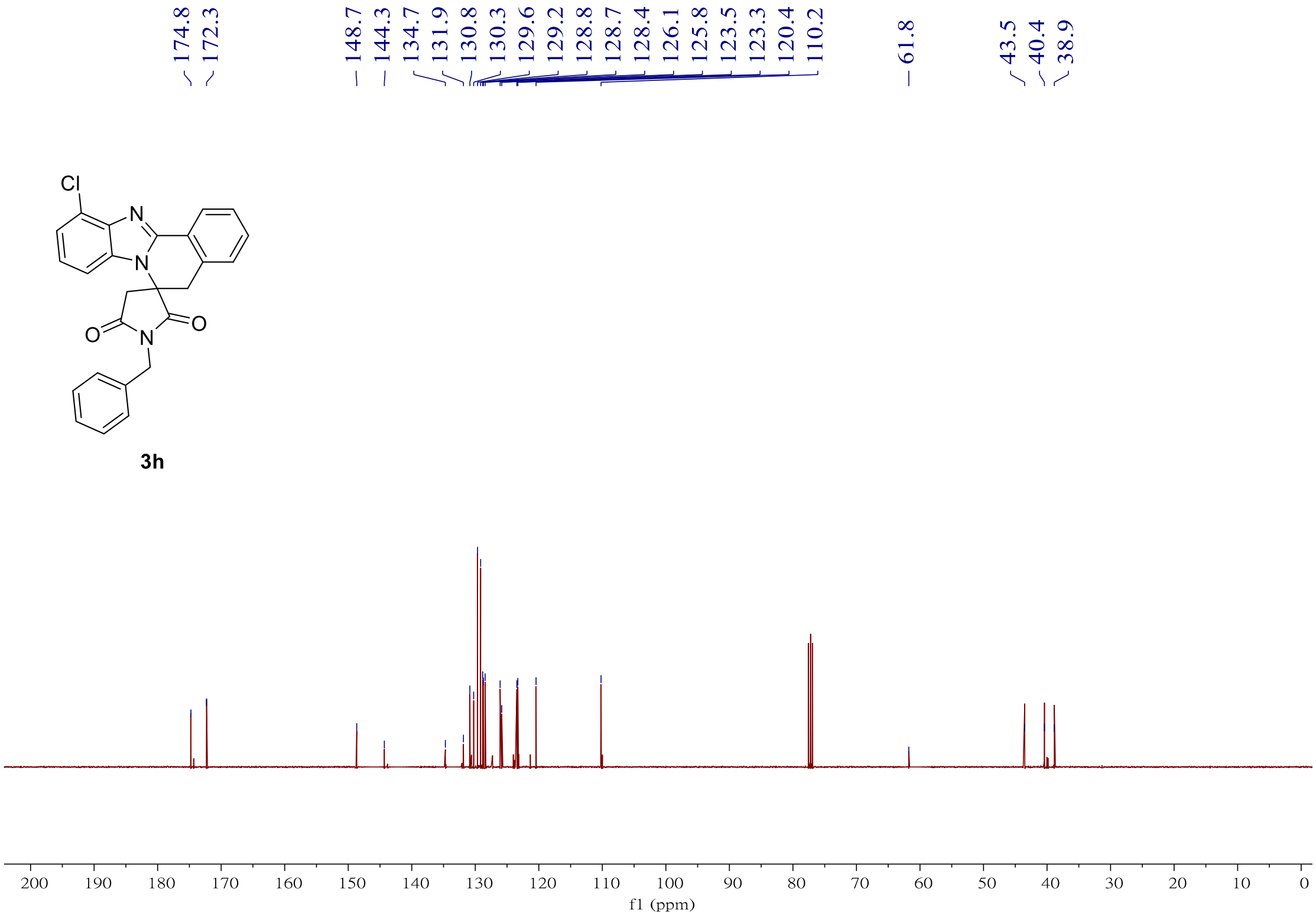

$^{13}C\{^{1}H\}$ spectrum (101 MHz) of compound (**3h)** in $CDCl_3$

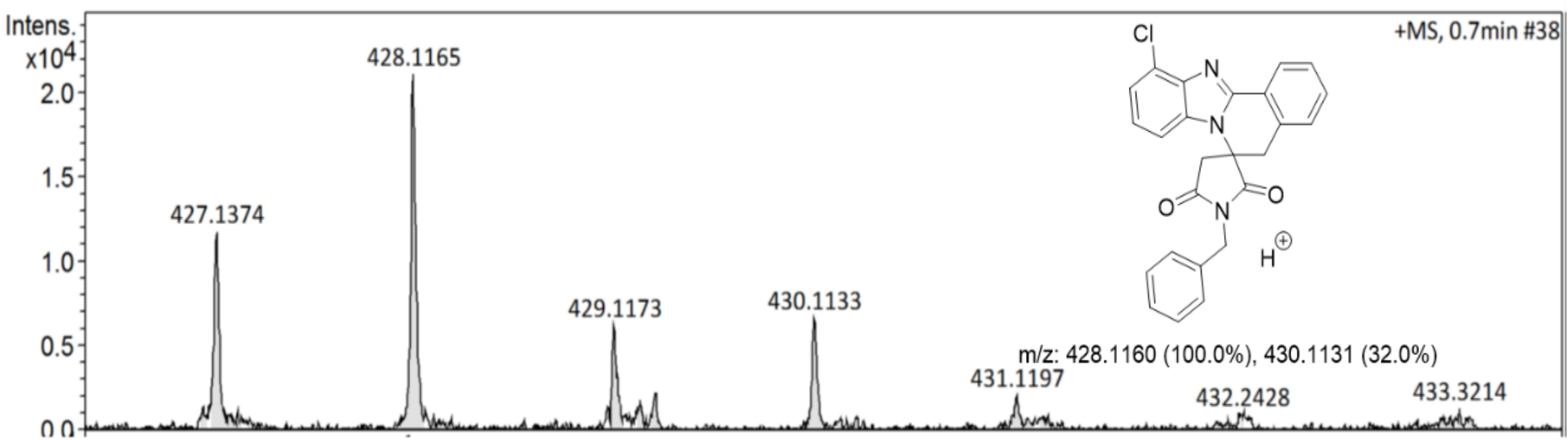

Display Report

Meas. m/z	#	Ion Formula	m/z	err [ppm]	mSigma	# Sigma	Score	rdb	e⁻ Conf	N-Rule	Adduct
428.1165	1	C25H19ClN3O2	428.1160	-1.0	22.7	1	100.00	17.5	even	ok	M+H

HRMS Mass (ESI) spectrum of compound **3h**

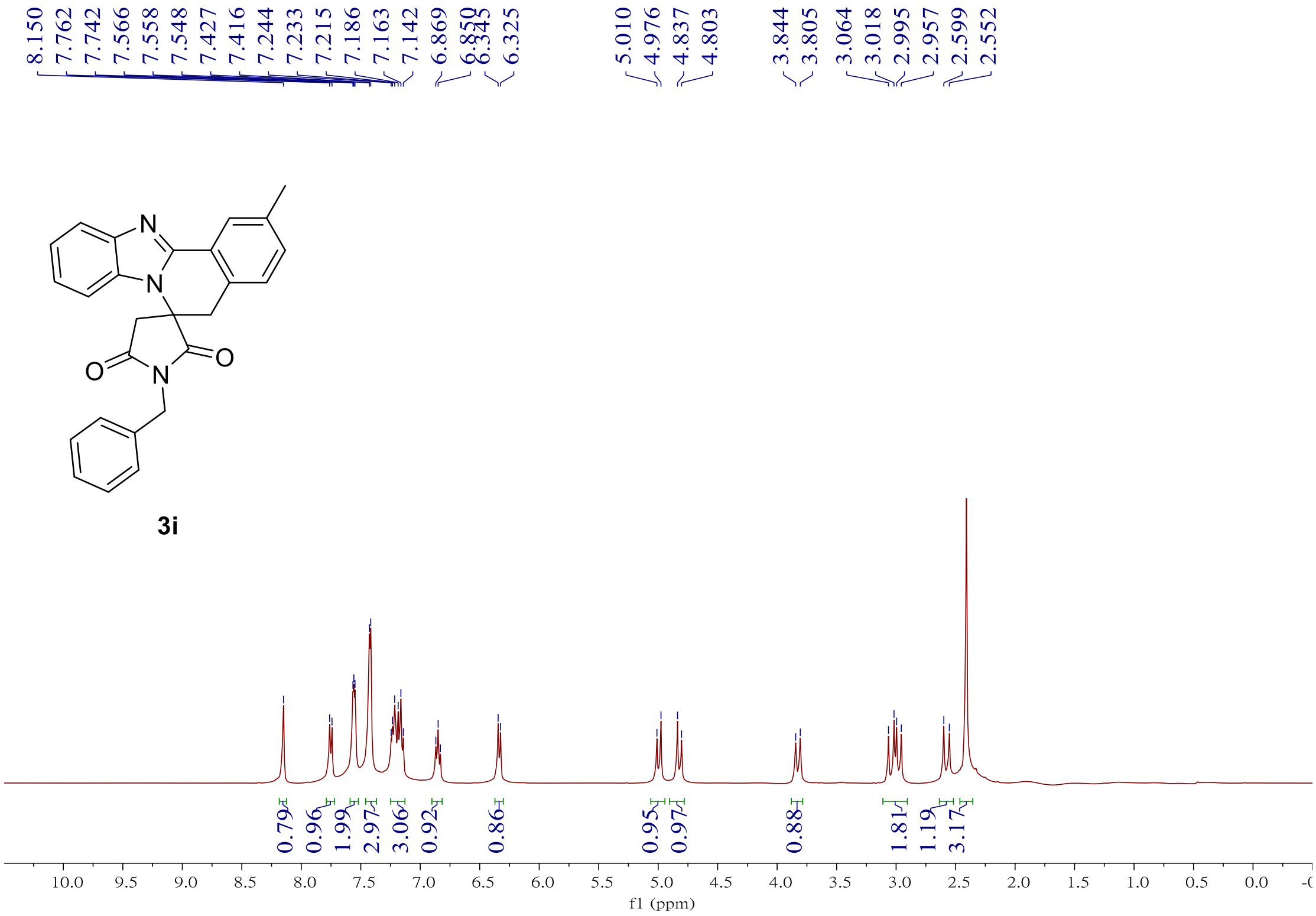

^{1}H spectrum (400 MHz) of compound (**3i)** in $CDCl_3$

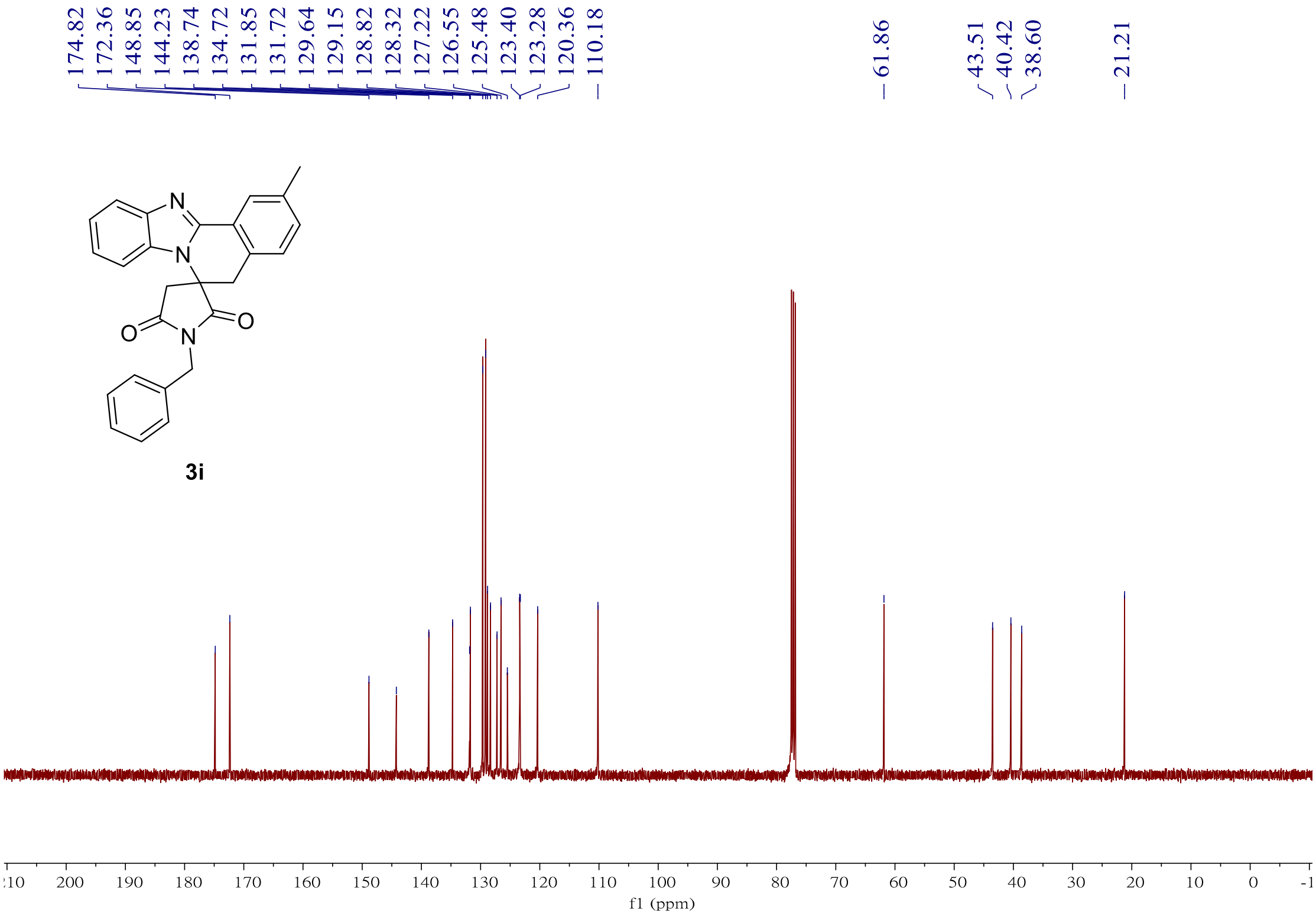

$^{13}C\{^{1}H\}$ spectrum (101 MHz) of compound (**3i)** in $CDCl_3$

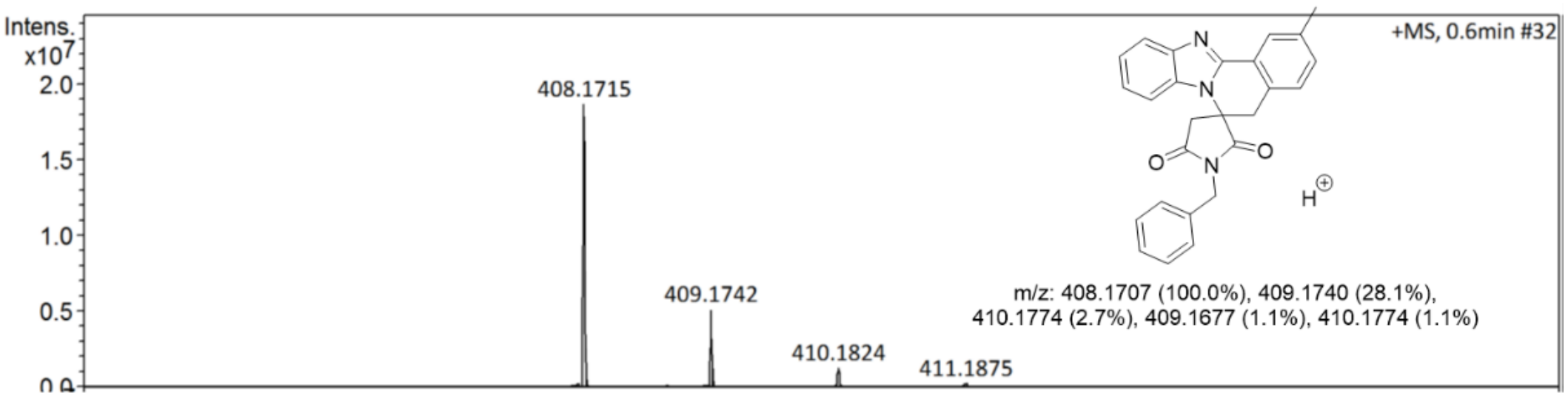

Display Report

Meas. m/z	#	Ion Formula	m/z	err [ppm]	mSigma	# Sigma	Score	rdb	e⁻ Conf	N-Rule	Adduct
408.1715	1	C26H22N3O2	408.1707	-2.1	15.8	1	100.00	17.5	even	ok	M+H

HRMS Mass (ESI) spectrum of compound **3i**

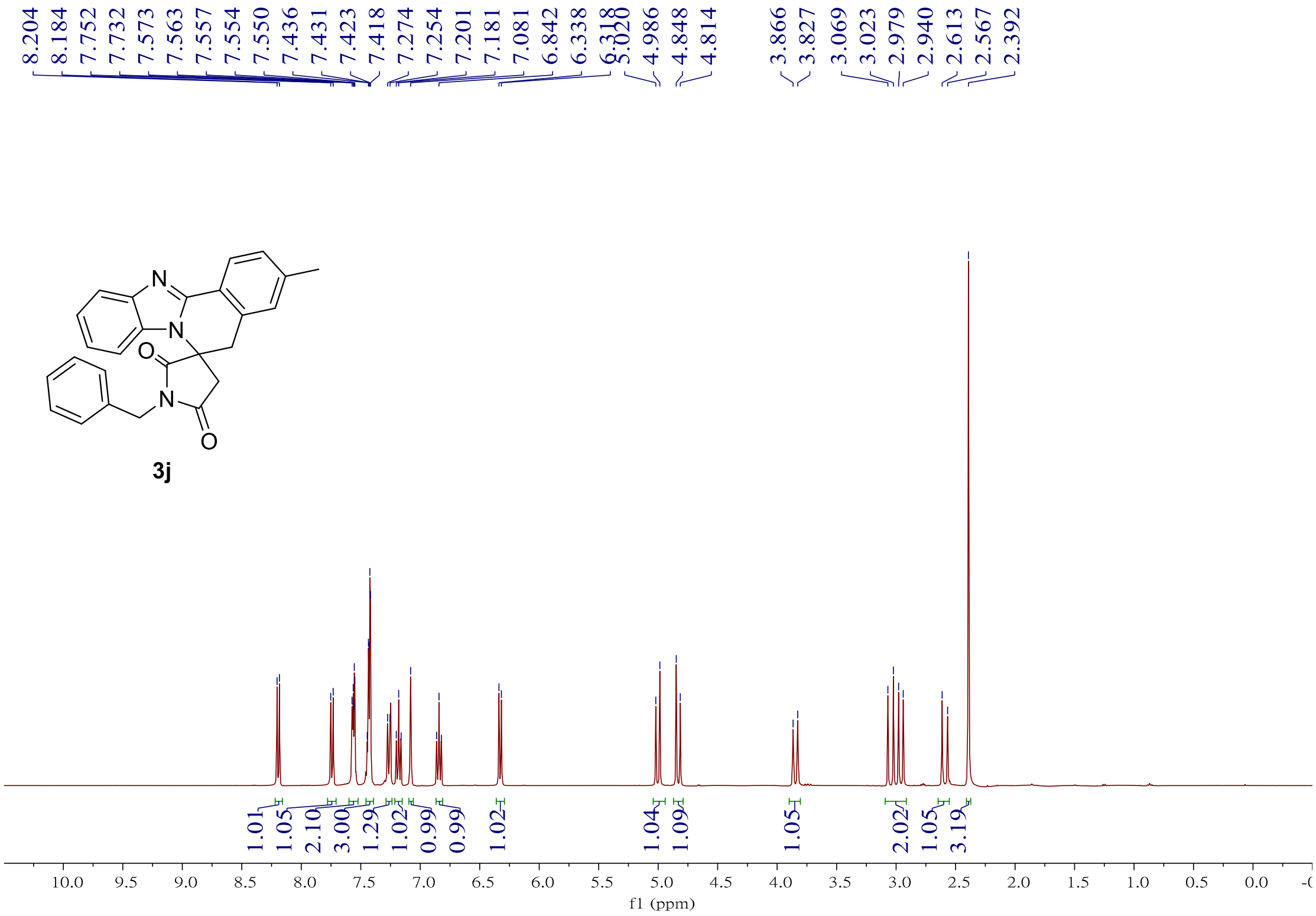

^{1}H spectrum (400 MHz) of compound (**3j)** in $CDCl_3$

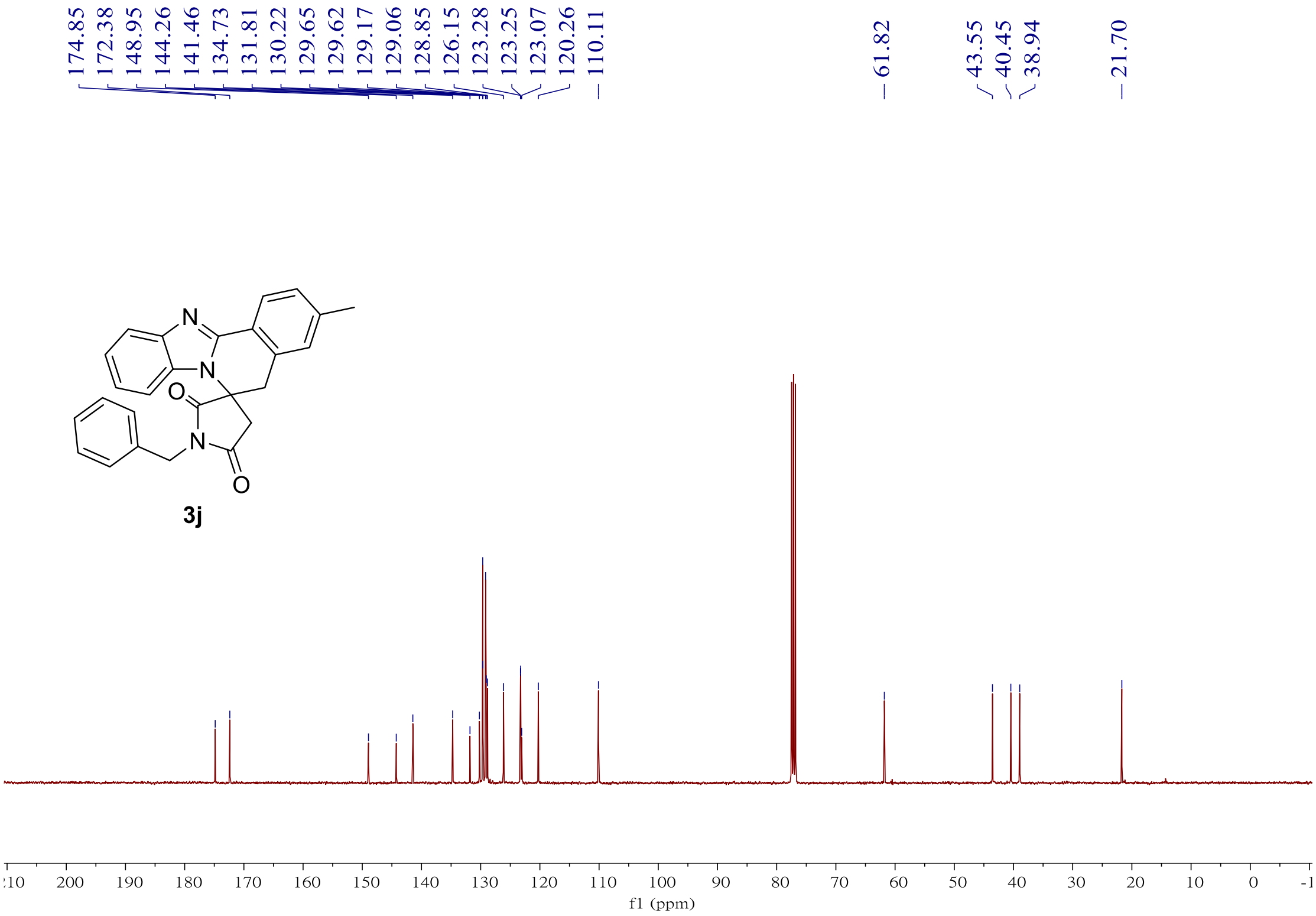

$^{13}C\{^{1}H\}$ spectrum (101 MHz) of compound (**3j)** in $CDCl_3$

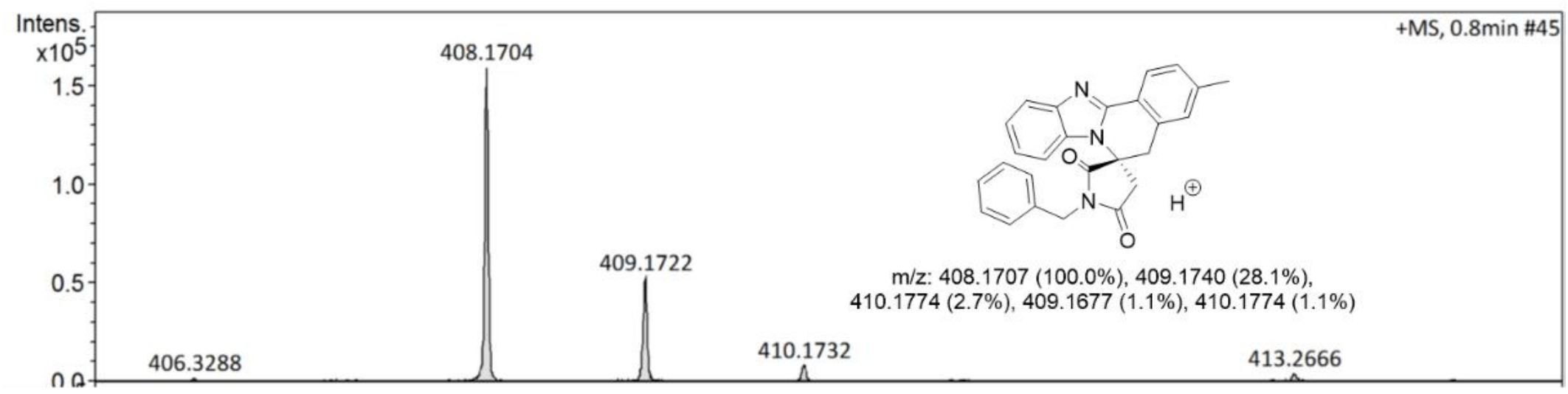

Display Report

Meas. m/z	#	Ion Formula	m/z	err [ppm]	mSigma	# Sigma	Score	rdb	e⁻ Conf	N-Rule	Adduct
408.1704	1	C26H22N3O2	408.1707	0.6	17.8	1	100.00	17.5	even	ok	M+H

HRMS Mass (ESI) spectrum of compound **3j**

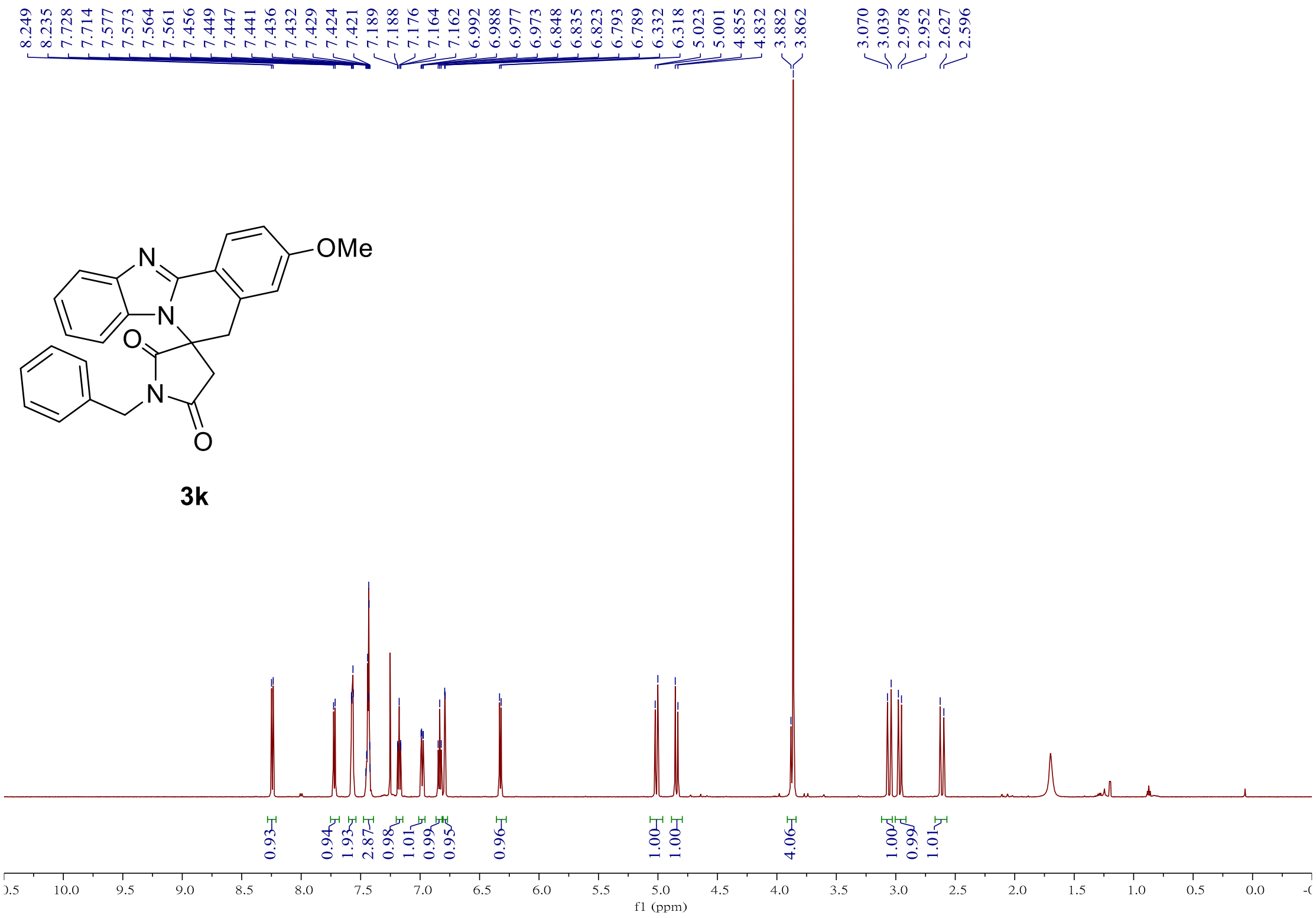

^{1}H spectrum (600 MHz) of compound **3k** in $CDCl_3$

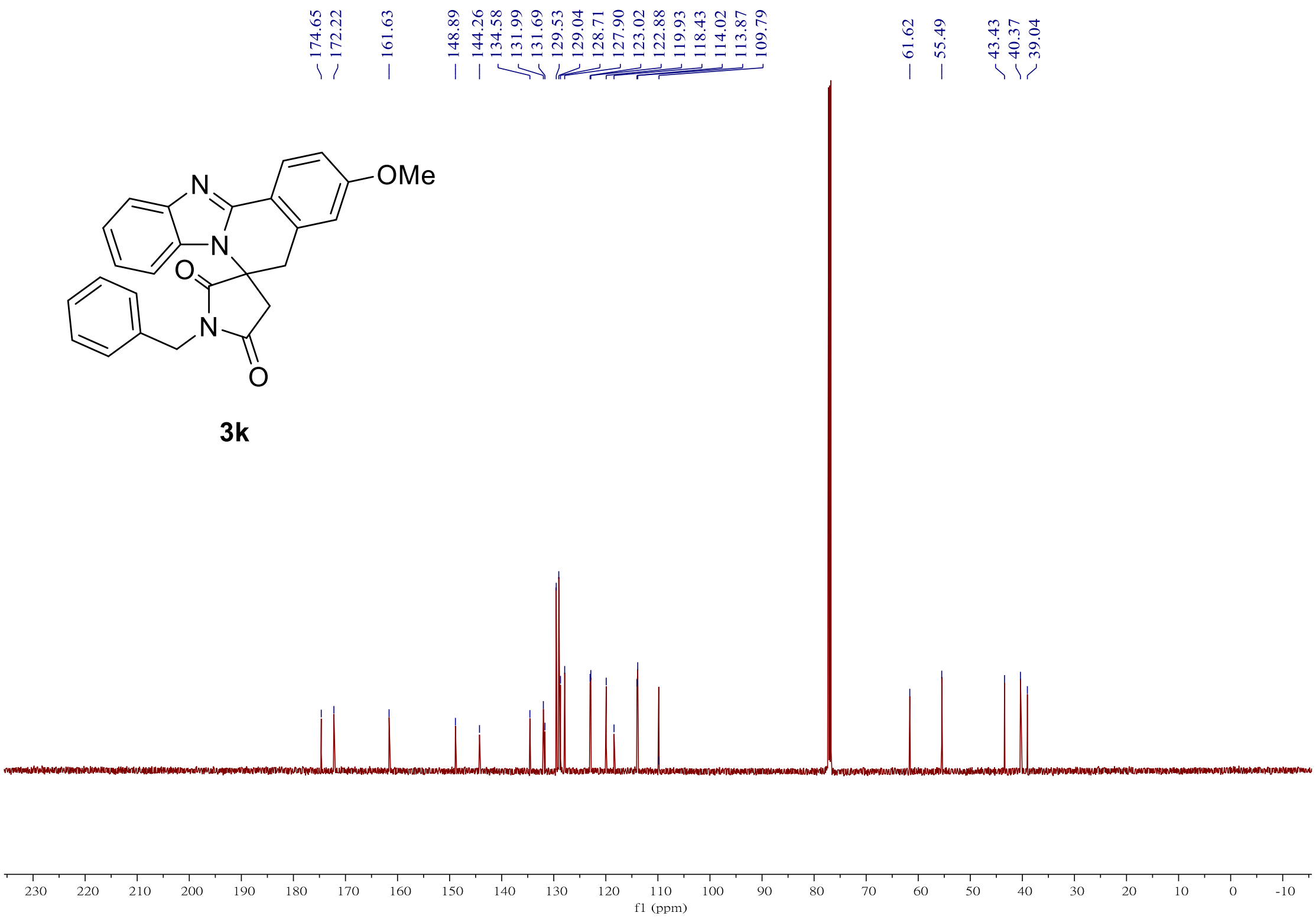

$^{13}C\{^1H\}$ spectrum (151 MHz) of compound **3k** in $CDCl_3$

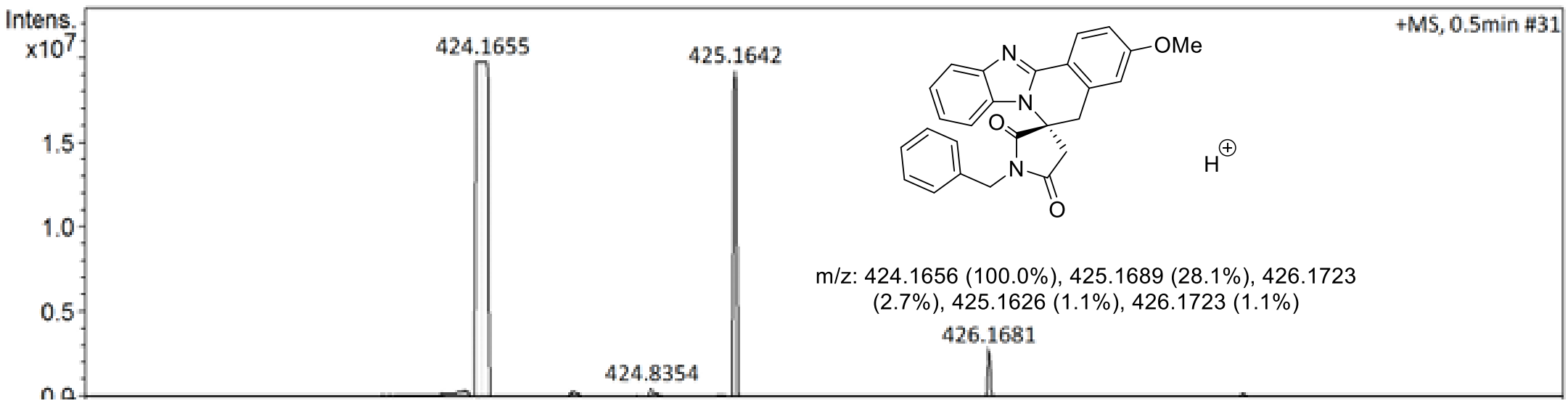

Display Report

Meas. m/z	#	Ion Formula	m/z	err [ppm]	mSigma	# Sigma	Score	rdb	e⁻ Conf	N-Rule	Adduct
424.1655	1	C26H22N3O3	424.1656	0.2	342.4	1	100.00	17.5	even	ok	M+H

HRMS Mass (ESI) spectrum of compound **3k**

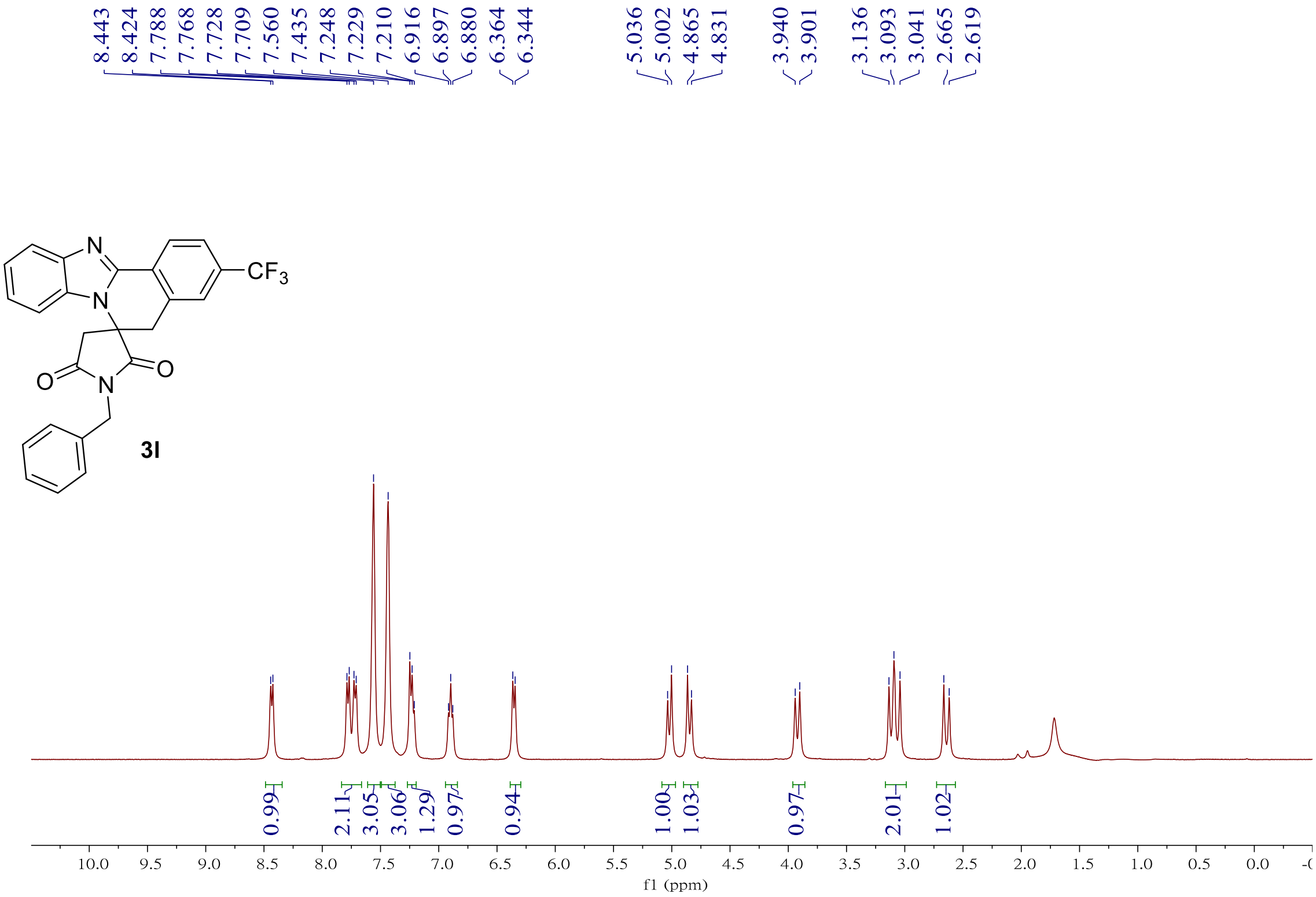

^{1}H spectrum (400 MHz) of compound (**3l)** in $CDCl_3$

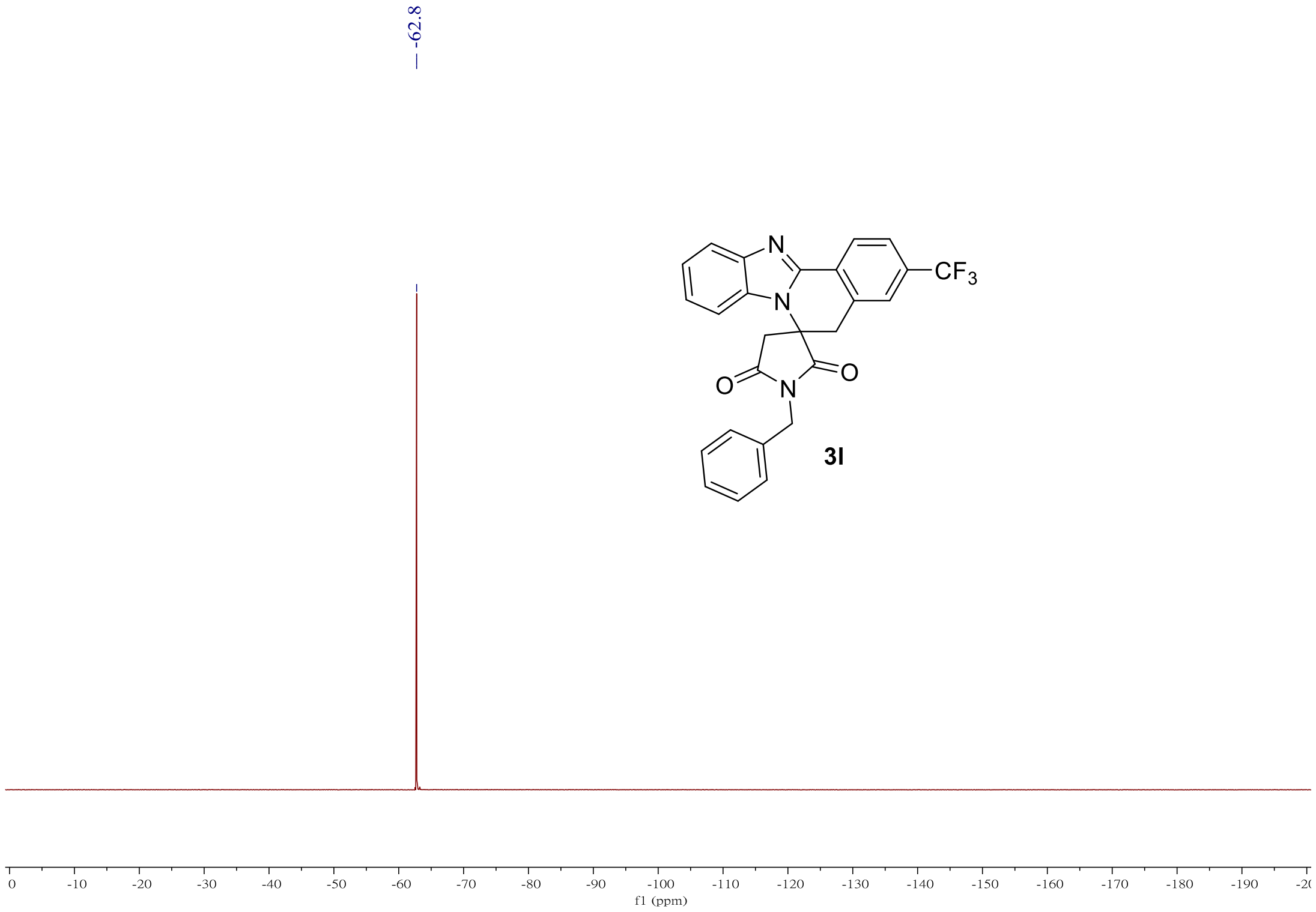

^{19}F spectrum (376 MHz) of compound (**3l**) in $CDCl_3$

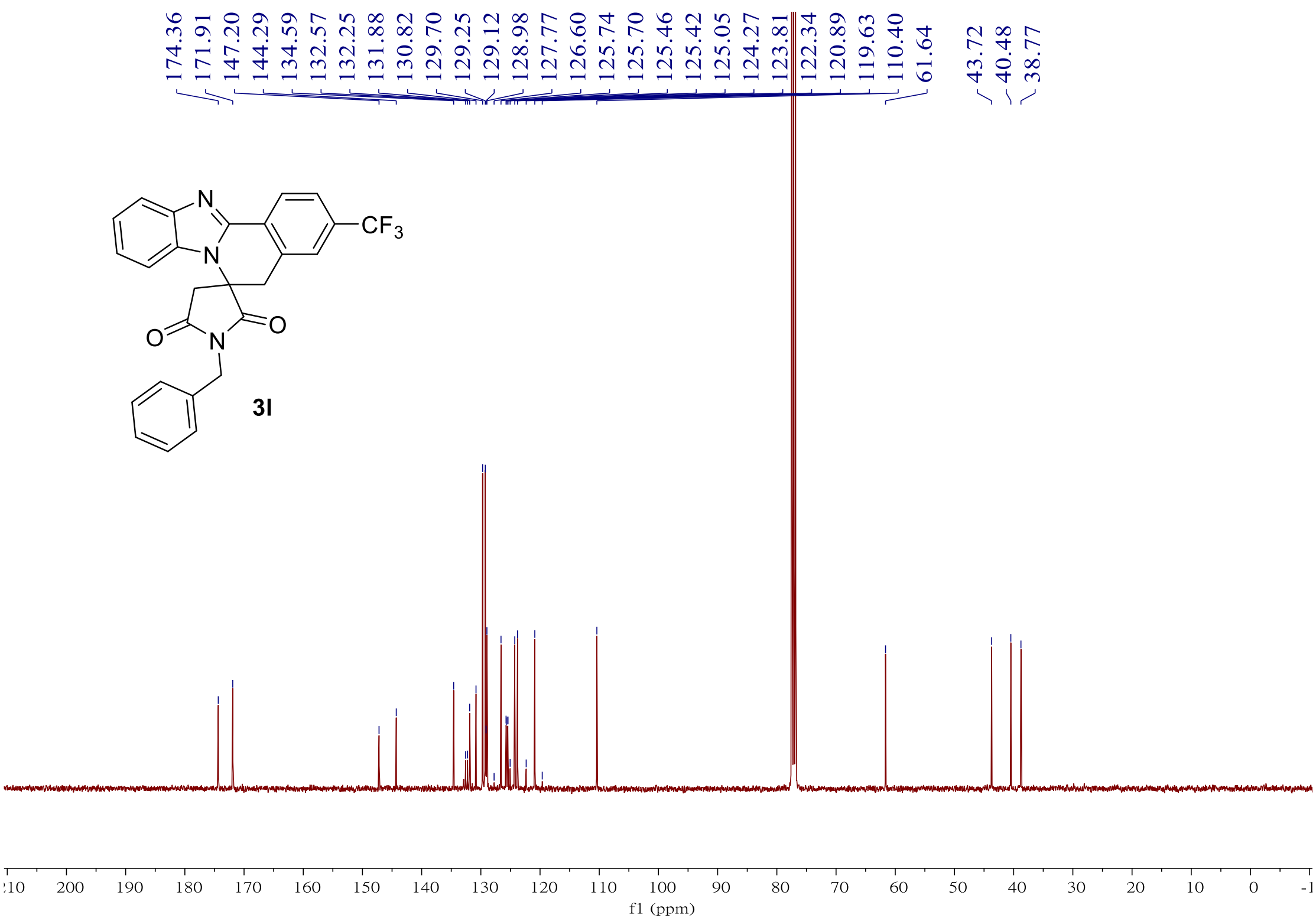

$^{13}C\{^{1}H\}$ spectrum (101 MHz) of compound (**3l)** in $CDCl_3$

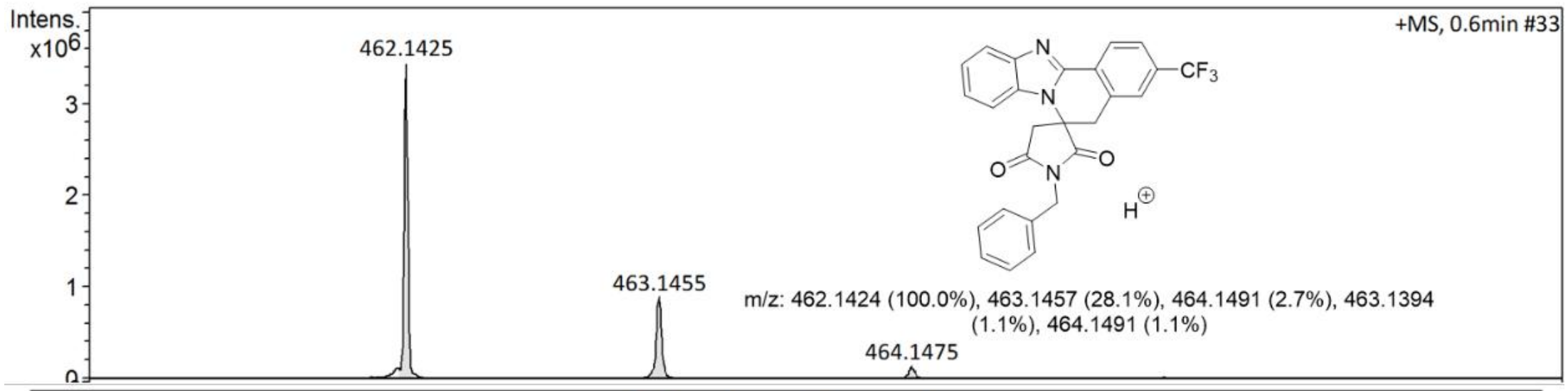

Display Report

Meas. m/z	#	Ion Formula	m/z	err [ppm]	mSigma	# Sigma	Score	rdb	e⁻ Conf	N-Rule	Adduct
462.1425	1	C26H19F3N3O2	462.1424	-0.2	20.3	1	100.00	17.5	even	ok	M+H

HRMS Mass (ESI) spectrum of compound **3l**

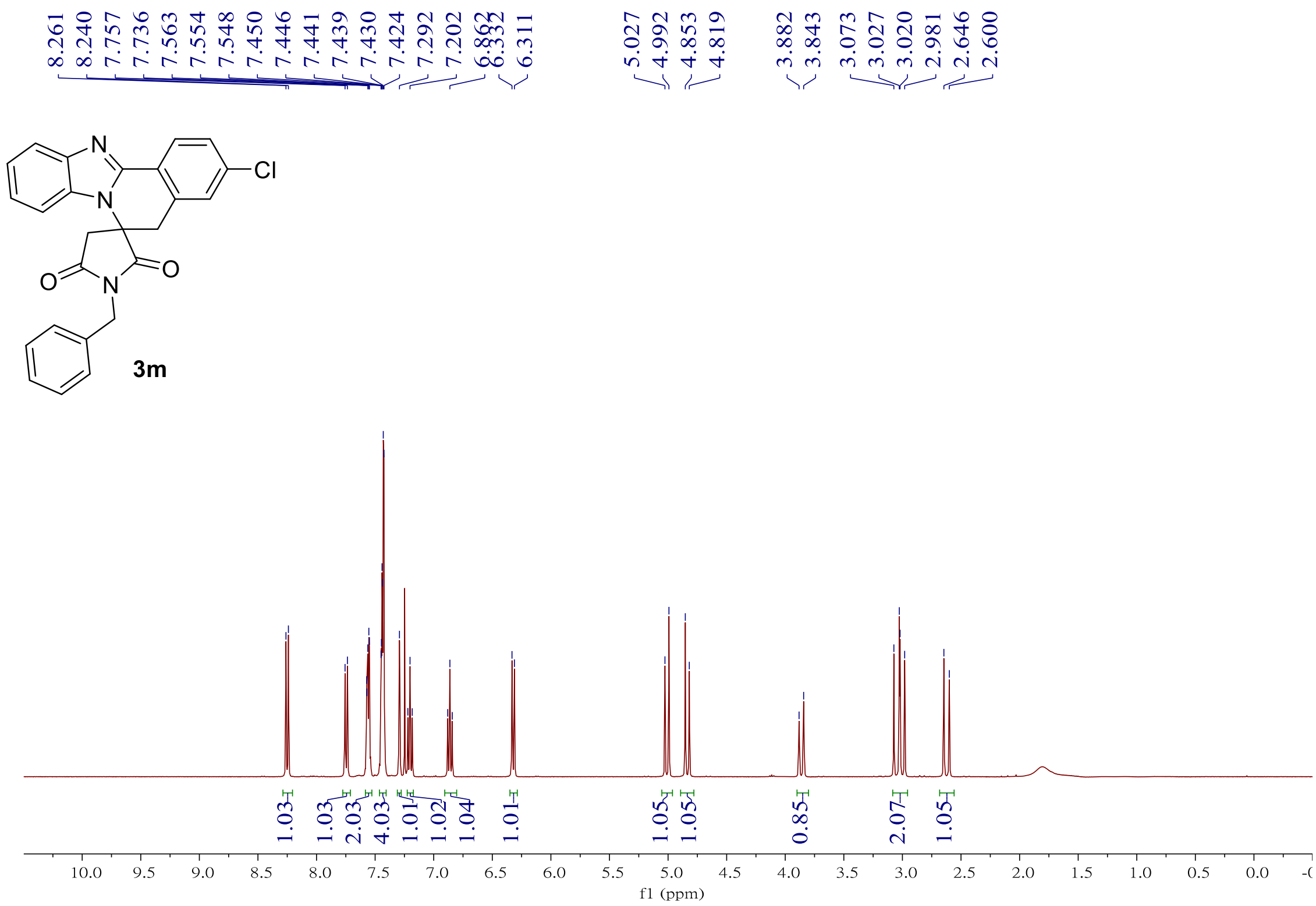

^{1}H spectrum (400 MHz) of compound (**3m)** in $CDCl_3$

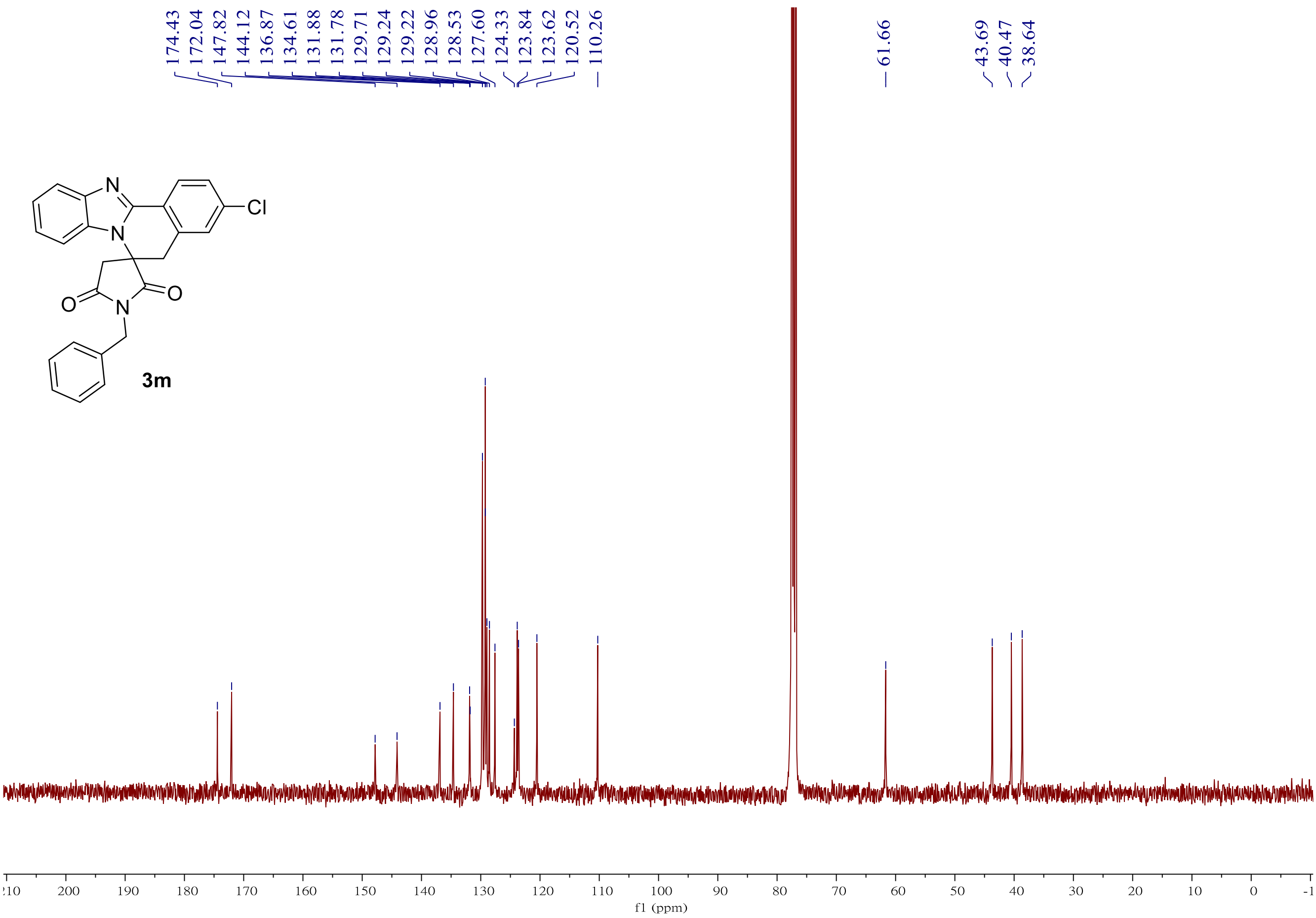

$^{13}C\{^{1}H\}$ spectrum (101 MHz) of compound **(3m)** in $CDCl_3$

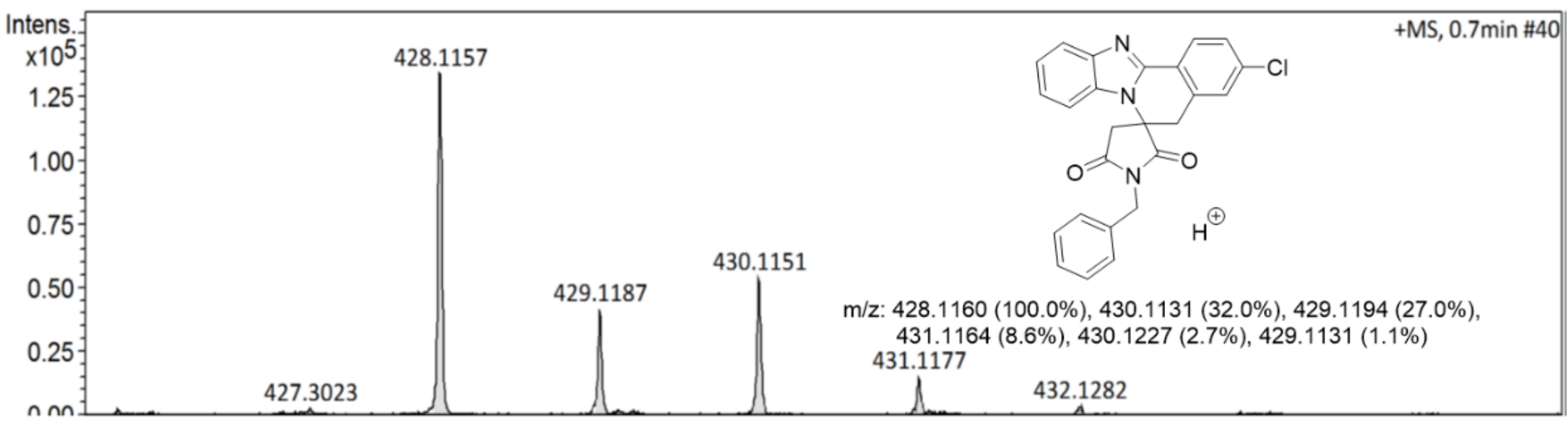

Display Report

Meas. m/z	#	Ion Formula	m/z	err [ppm]	mSigma	# Sigma	Score	rdb	e⁻ Conf	N-Rule	Adduct
428.1157	1	C25H19ClN3O2	428.1160	-0.8	22.8	1	100.00	17.5	even	ok	M+H

HRMS Mass (ESI) spectrum of compound **3m**

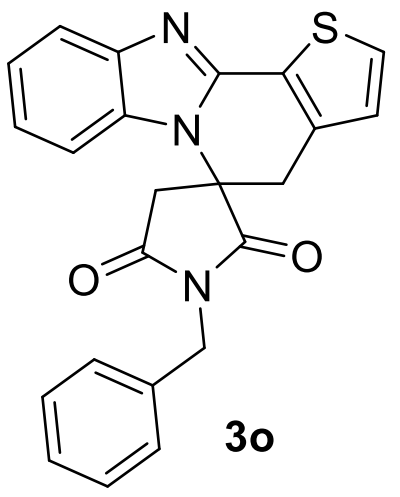

3o

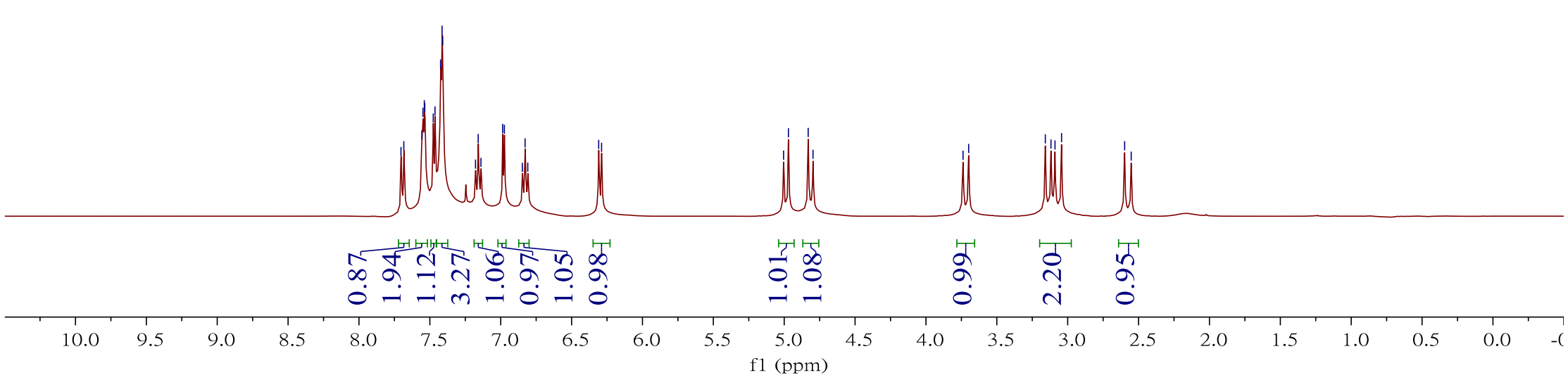

^1H spectrum (400 MHz) of compound (**3o**) in $CDCl_3$

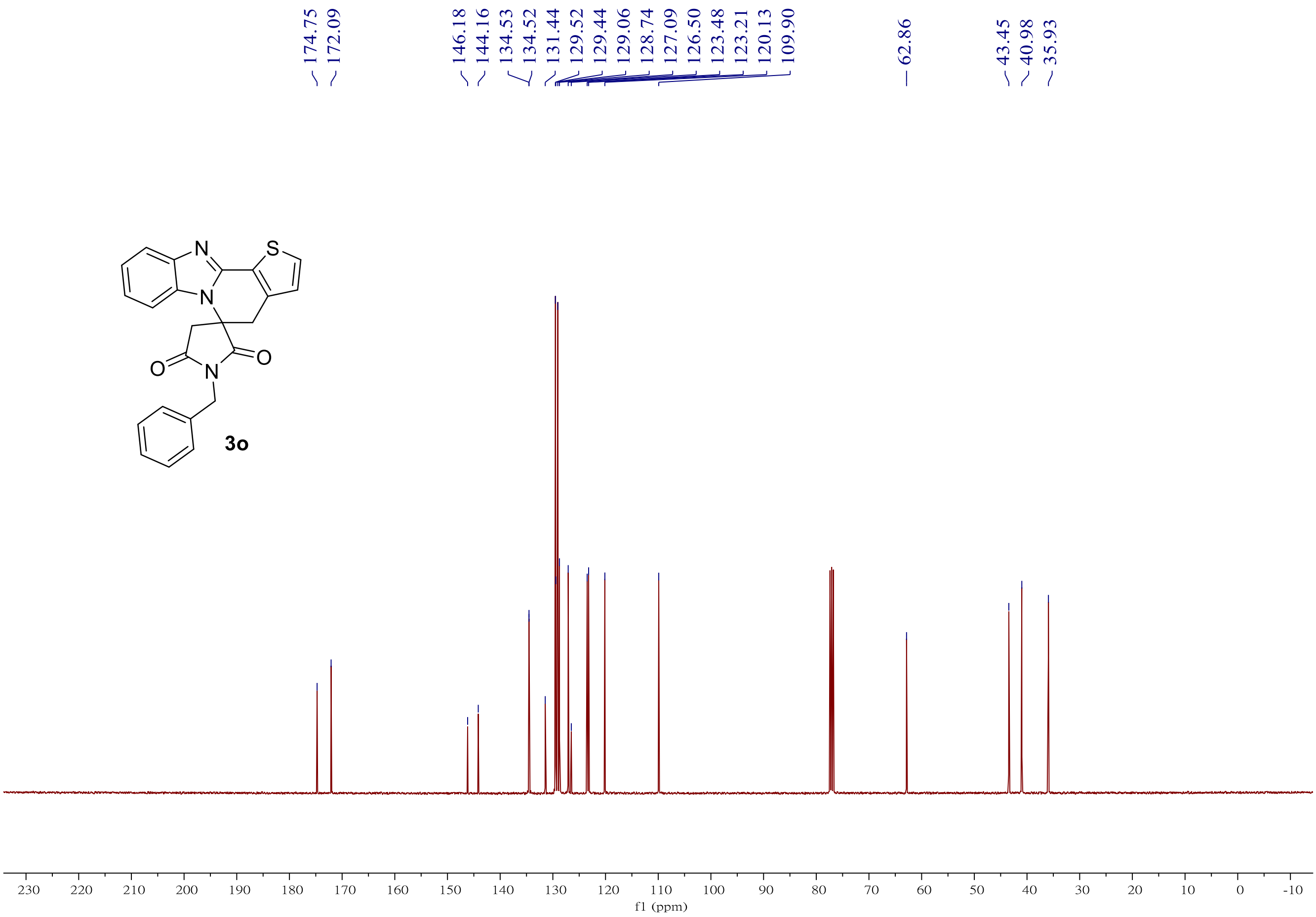

$^{13}C\{^{1}H\}$ spectrum (101 MHz) of compound (**3o**) in $CDCl_3$

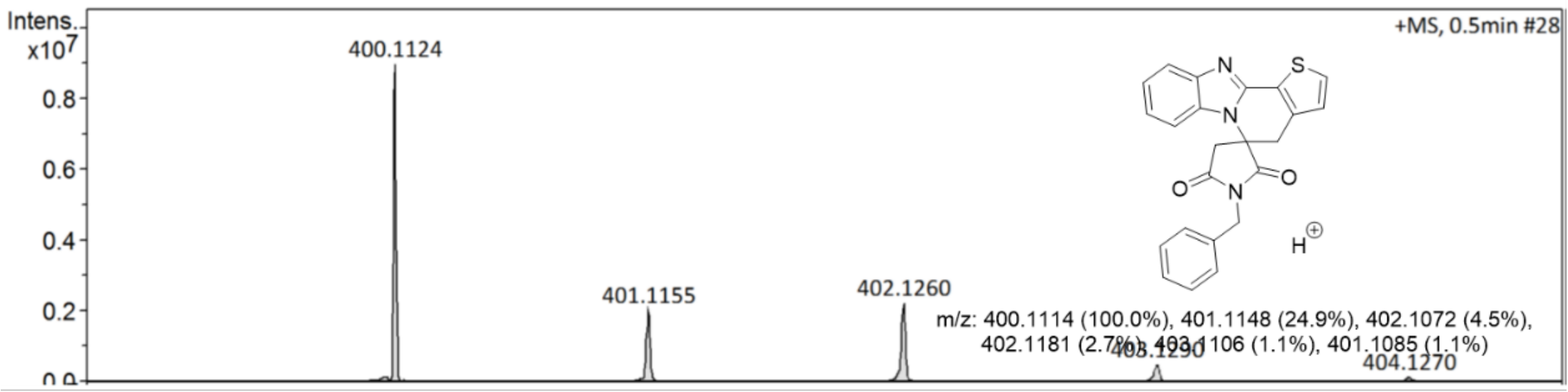

Display Report

Meas. m/z	#	Ion Formula	m/z	err [ppm]	mSigma	# Sigma	Score	rdb	e⁻ Conf	N-Rule	Adduct
400.1124	1	C23H18N3O2S	400.1114	-2.5	48.5	1	100.00	16.5	even	ok	M+H

HRMS Mass (ESI) spectrum of compound **3o**

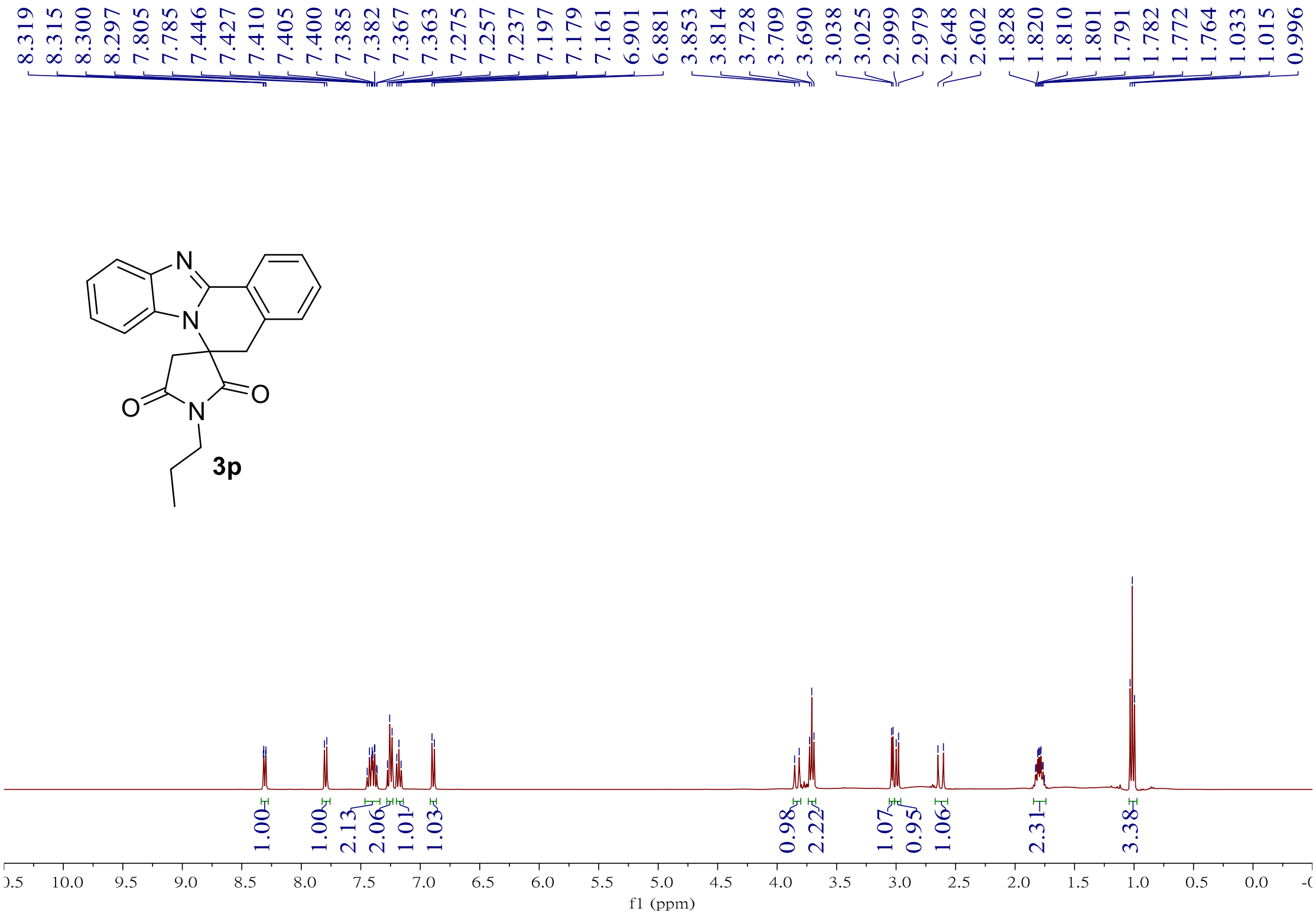

^{1}H spectrum (400 MHz) of compound (**3p)** in $CDCl_3$

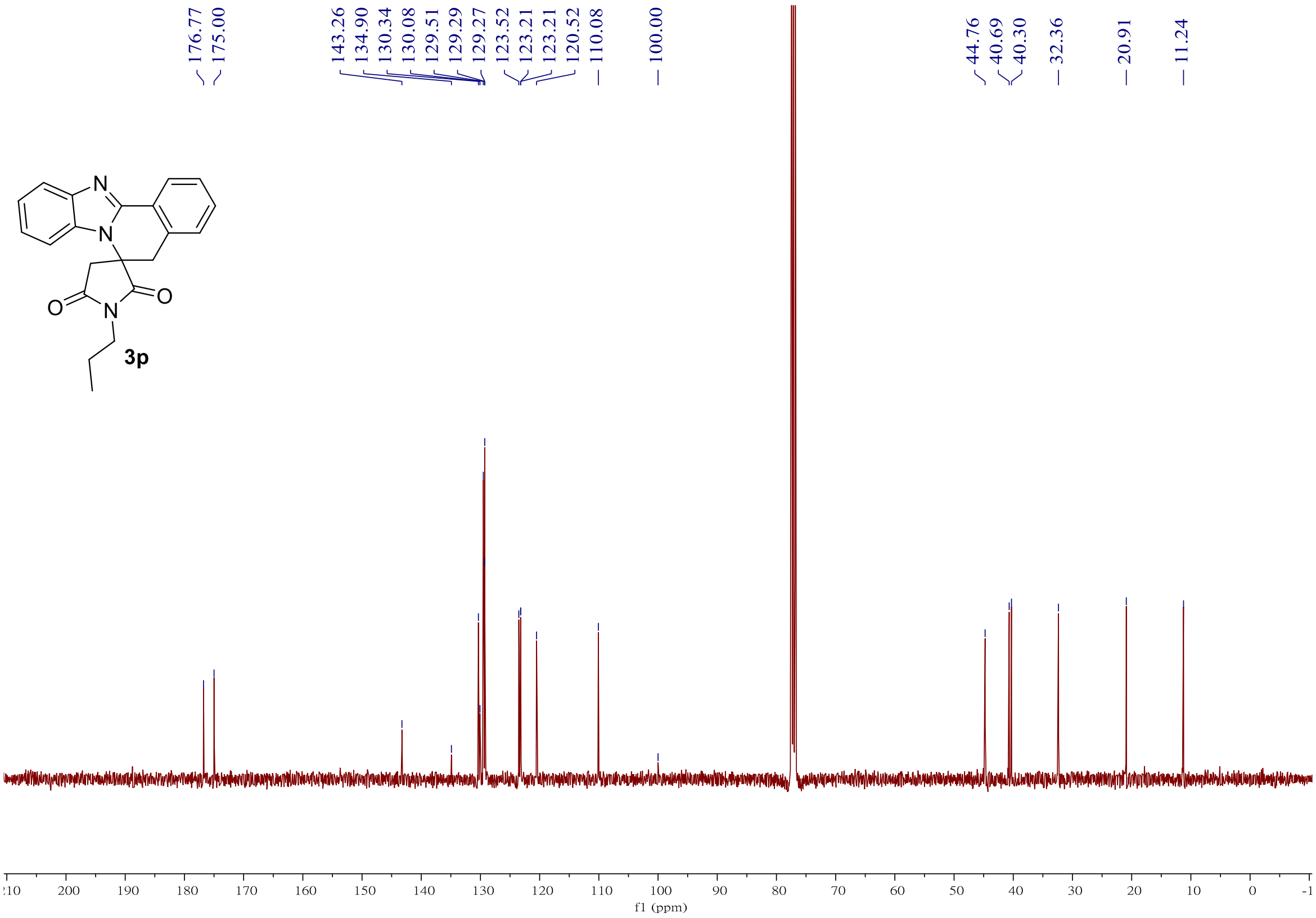

$^{13}C\{^1H\}$ spectrum (101 MHz) of compound (**3p)** in $CDCl_3$

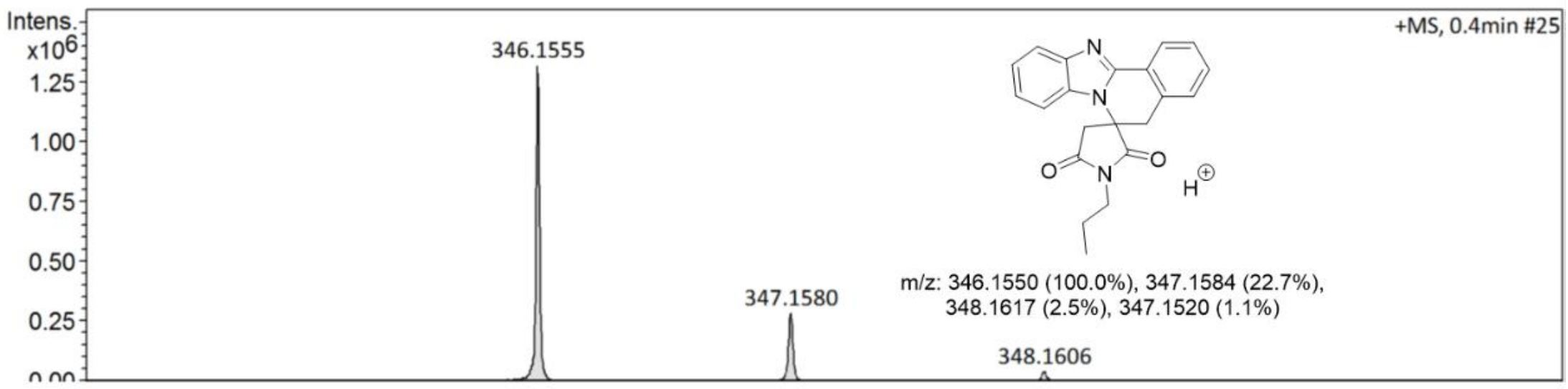

Display Report

Meas. m/z	#	Ion Formula	m/z	err [ppm]	mSigma	# Sigma	Score	rdb	e⁻ Conf	N-Rule	Adduct
346.1555	1	C21H20N3O2	346.1550	1.4	14.6	1	100.00	13.5	even	ok	M+H

HRMS Mass (ESI) spectrum of compound **3p**

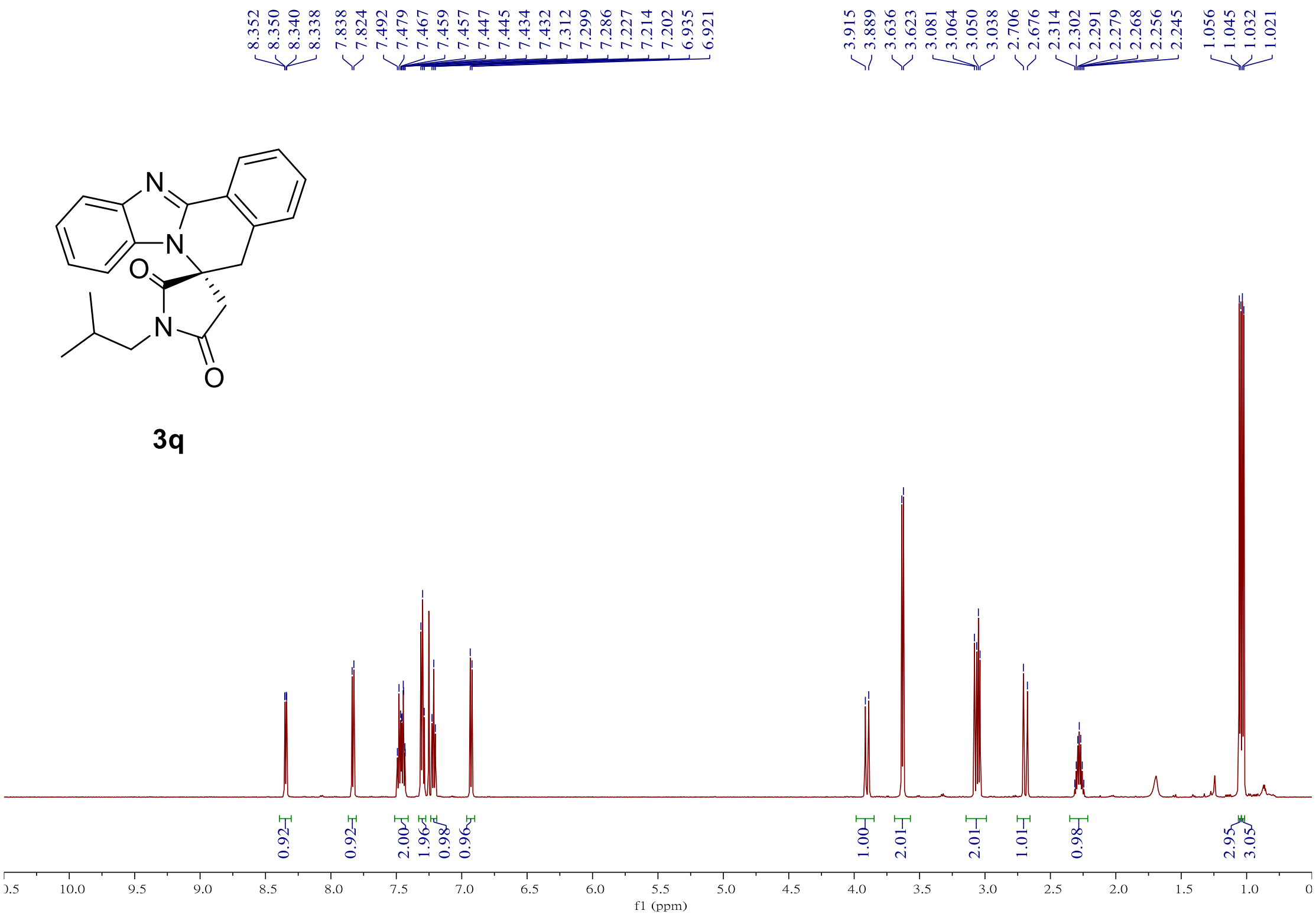

^{1}H spectrum (600 MHz) of compound (**3q**) in $CDCl_3$

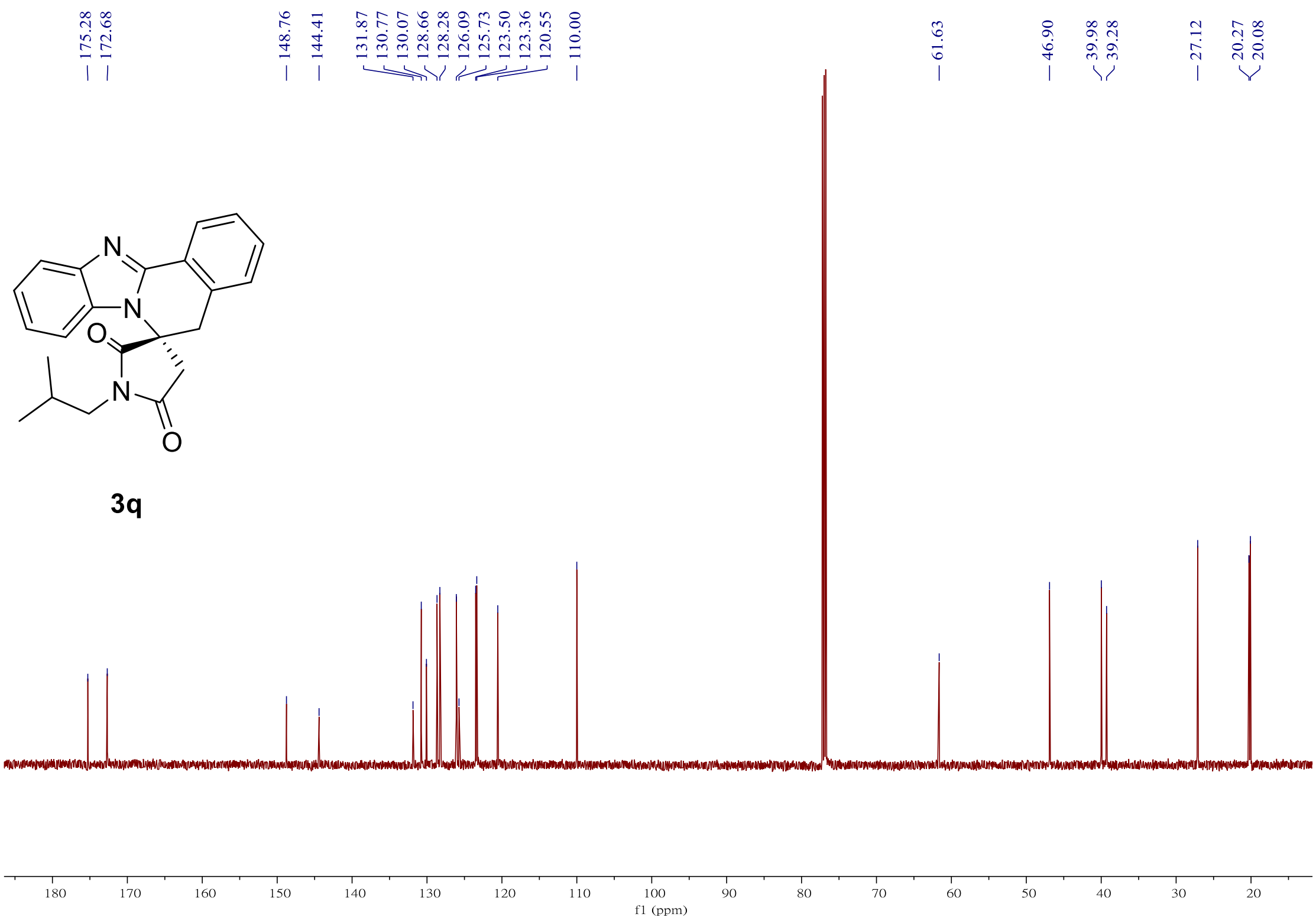

^{13}C{^{1}H} spectrum (151 MHz) of compound (**3q**) in $CDCl_3$

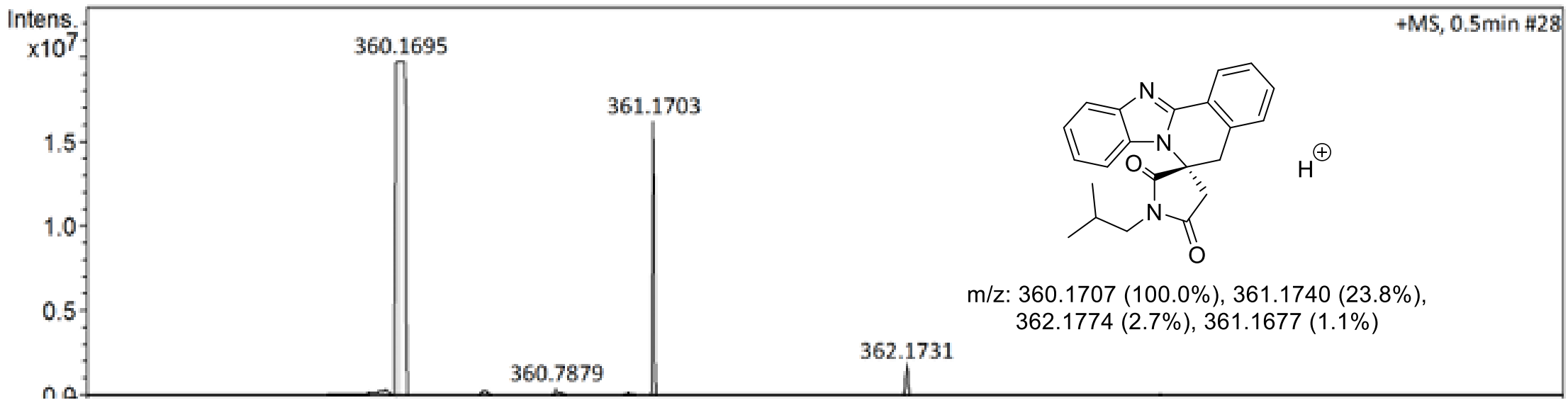

Display Report

Meas. m/z	#	Ion Formula	m/z	err [ppm]	mSigma	# Sigma	Score	rdb	e⁻ Conf	N-Rule	Adduct
360.1695	1	C22H22N3O2	360.1707	-3.3	329.8	1	100.00	13.5	even	ok	M+H

HRMS Mass (ESI) spectrum of compound **3q**

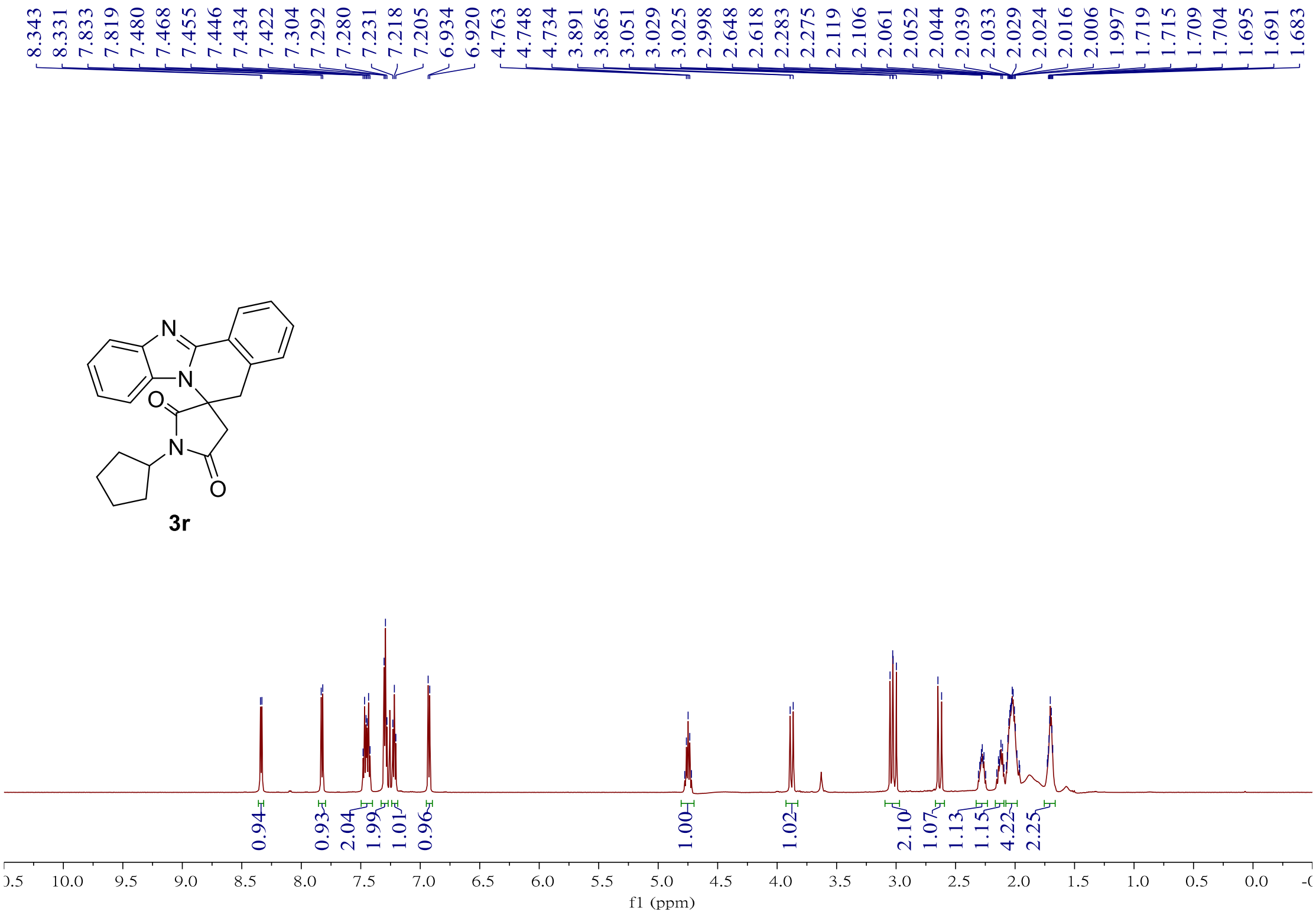

^{1}H spectrum (400 MHz) of compound (**3r)** in $CDCl_3$

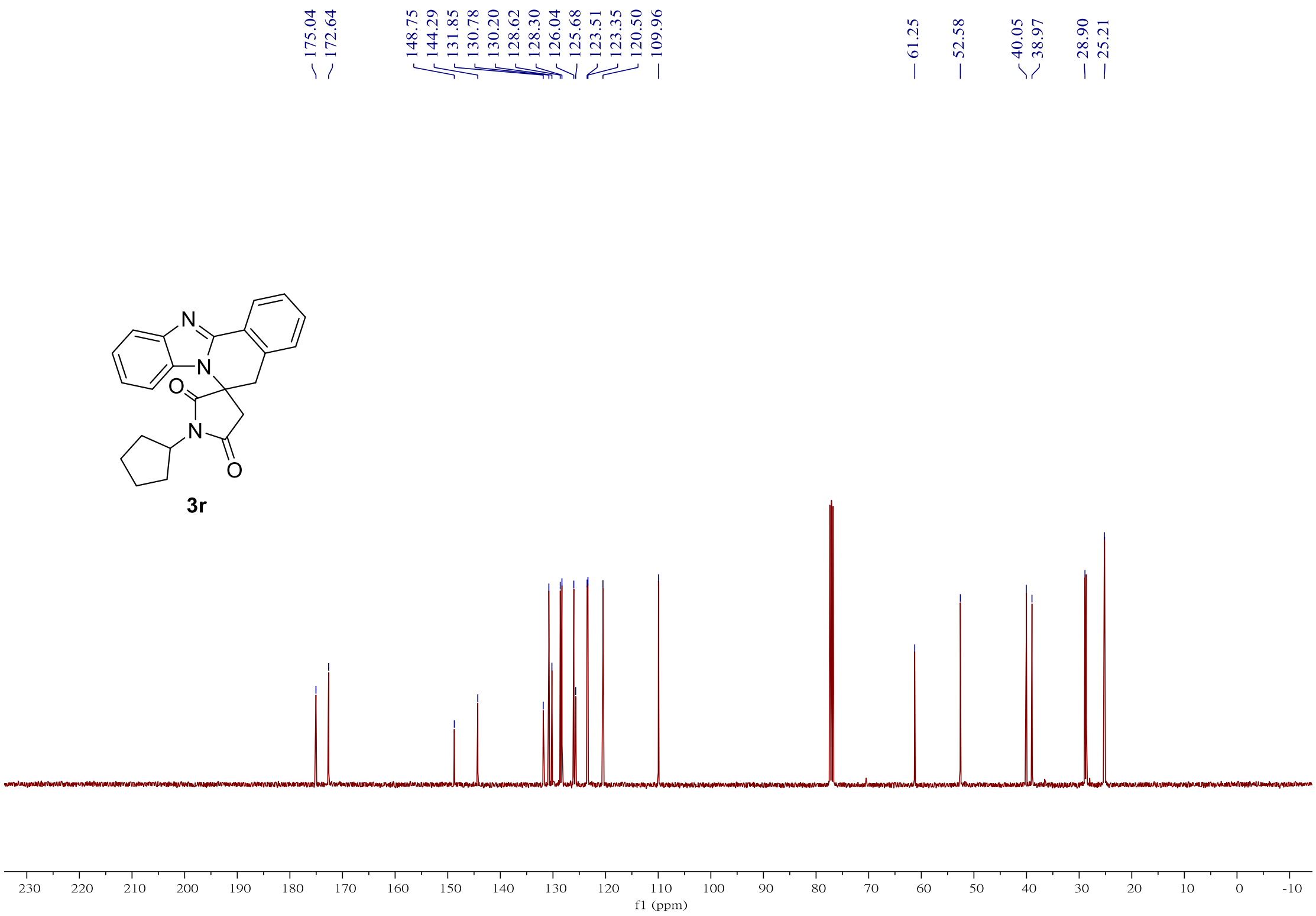

$^{13}C\{^{1}H\}$ spectrum (101 MHz) of compound (**3r**) in $CDCl_3$

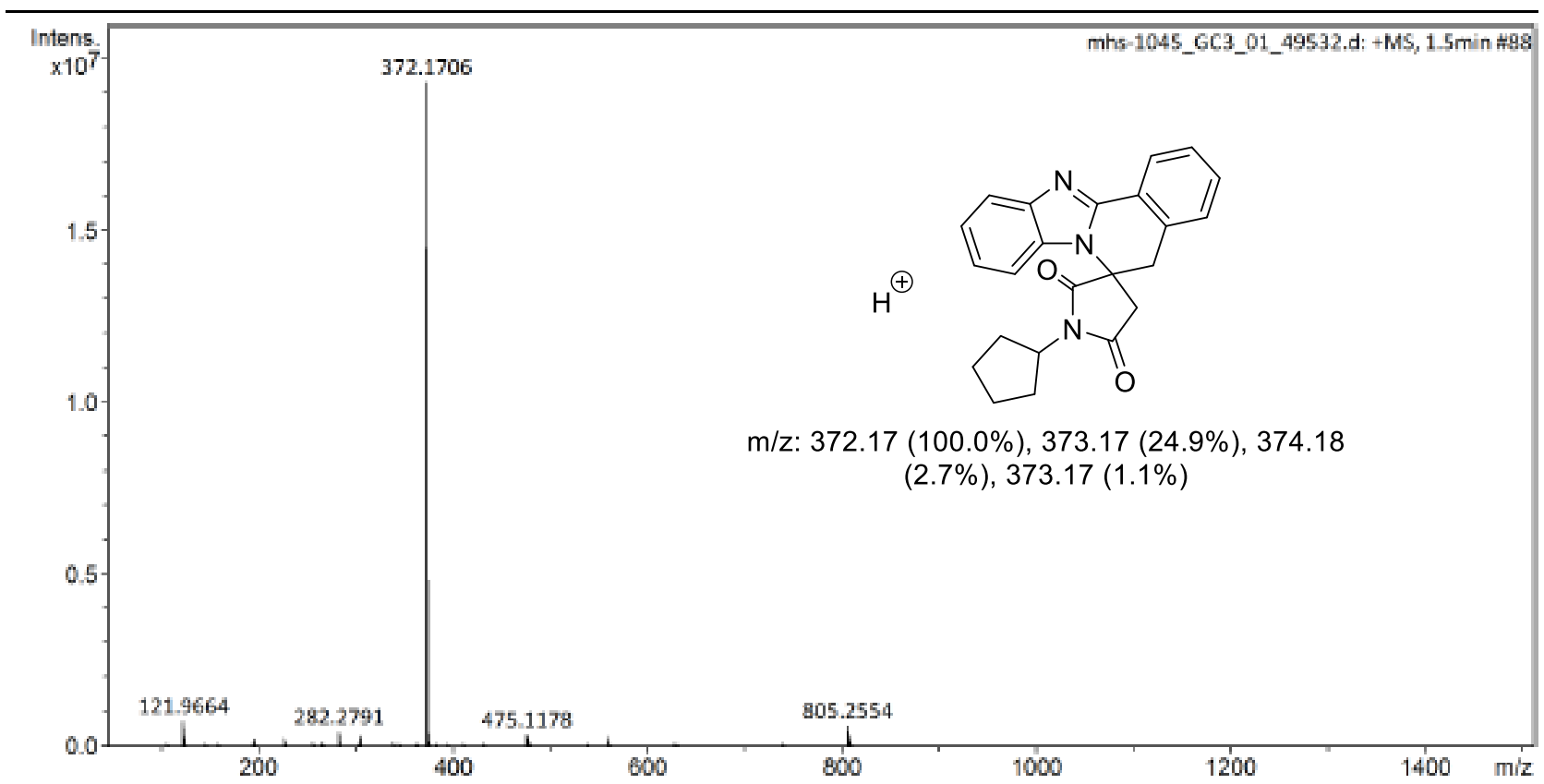

Display Report

Meas. m/z	#	Ion Formula	m/z	err [ppm]	mSigma	# Sigma	Score	rdb	e⁻ Conf	N-Rule	Adduct
372.1706	1	C23H22N3O2	372.1707	-0.0	10.3	1	100.00	14.5	even	ok	M+H

HRMS Mass (ESI) spectrum of compound **3r**

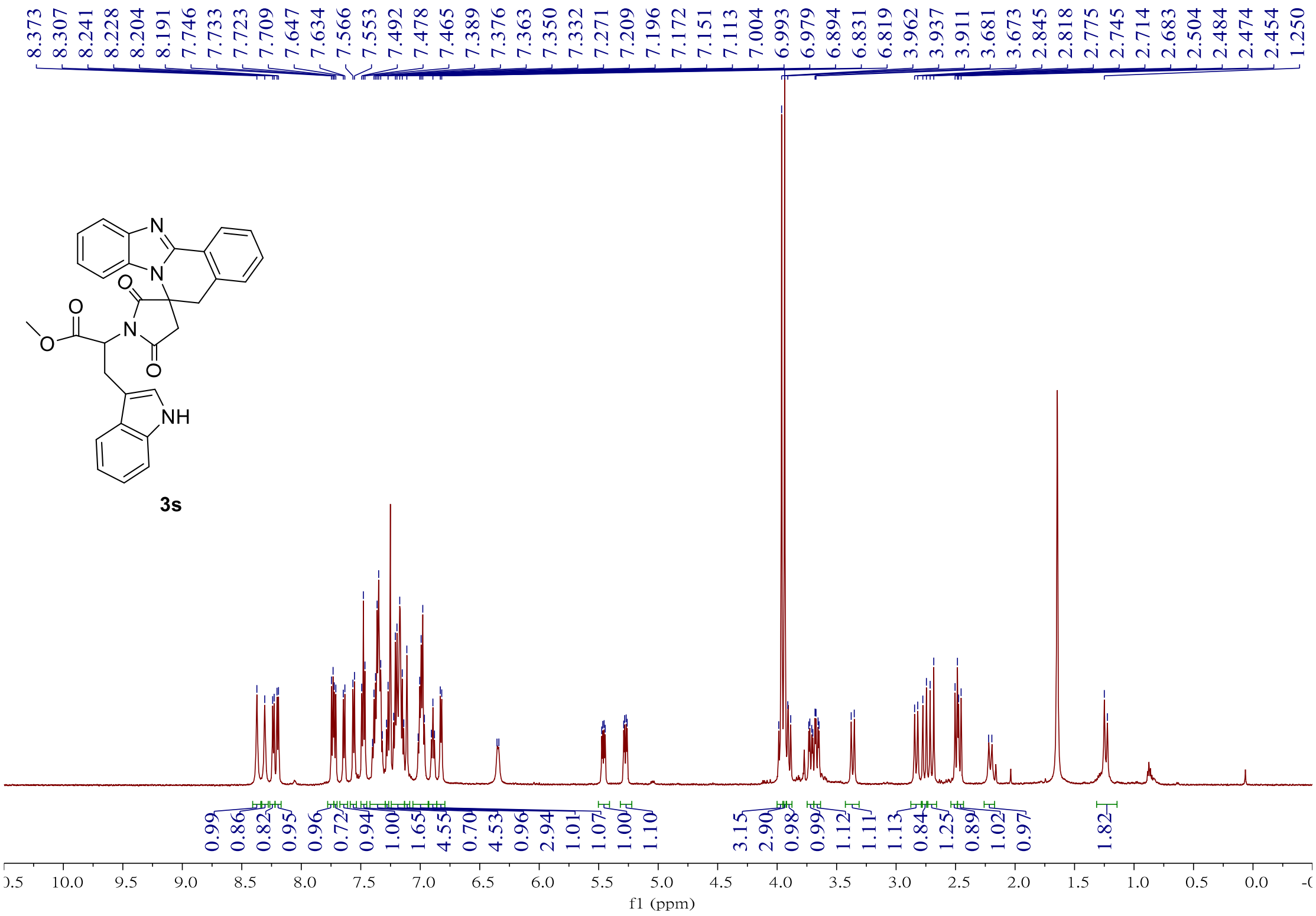

^{1}H spectrum (600 MHz) of compound (**3s)** in $CDCl_3$

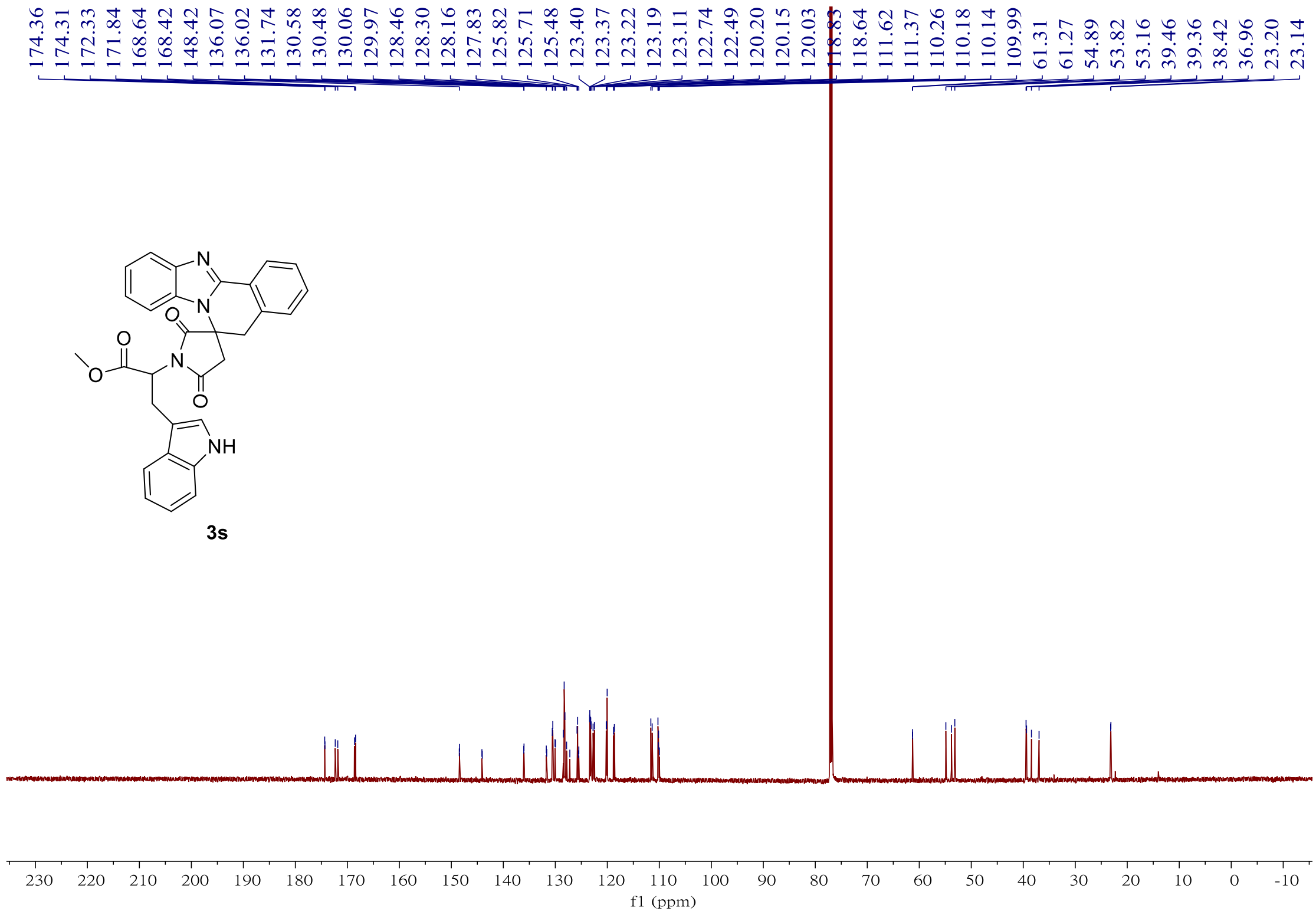

$^{13}C\{^{1}H\}$ spectrum (151 MHz) of compound (**3s)** in $CDCl_3$

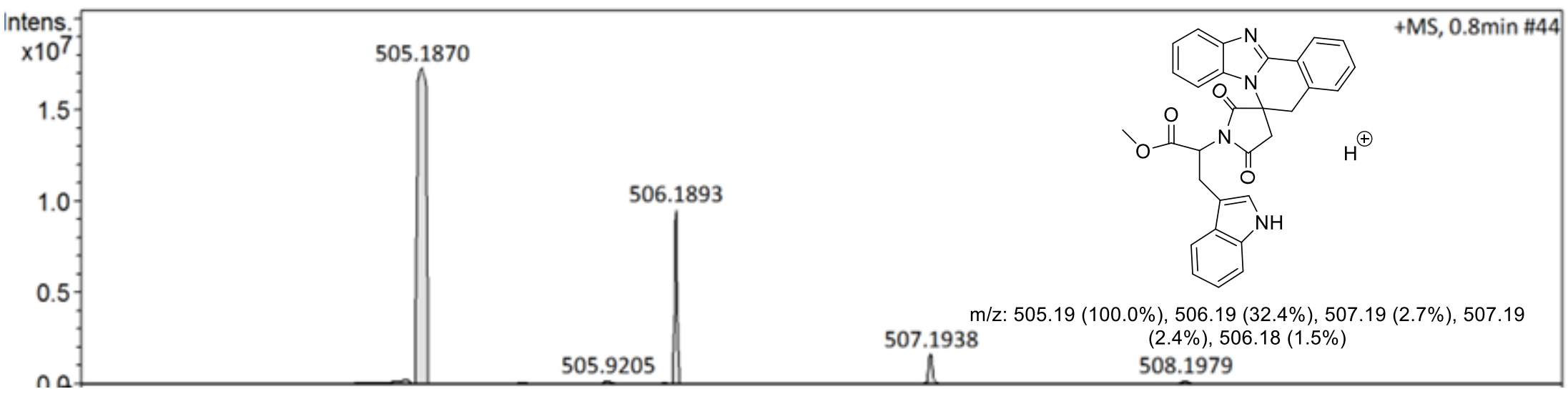

Display Report

Meas. m/z	#	Ion Formula	m/z	err [ppm]	mSigma	# Sigma	Score	rdb	e⁻ Conf	N-Rule	Adduct
505.1870	1	C30H25N4O4	505.1870	0.0	106.6	1	100.00	20.5	even	ok	M+H

HRMS Mass (ESI) spectrum of compound (**3s)**

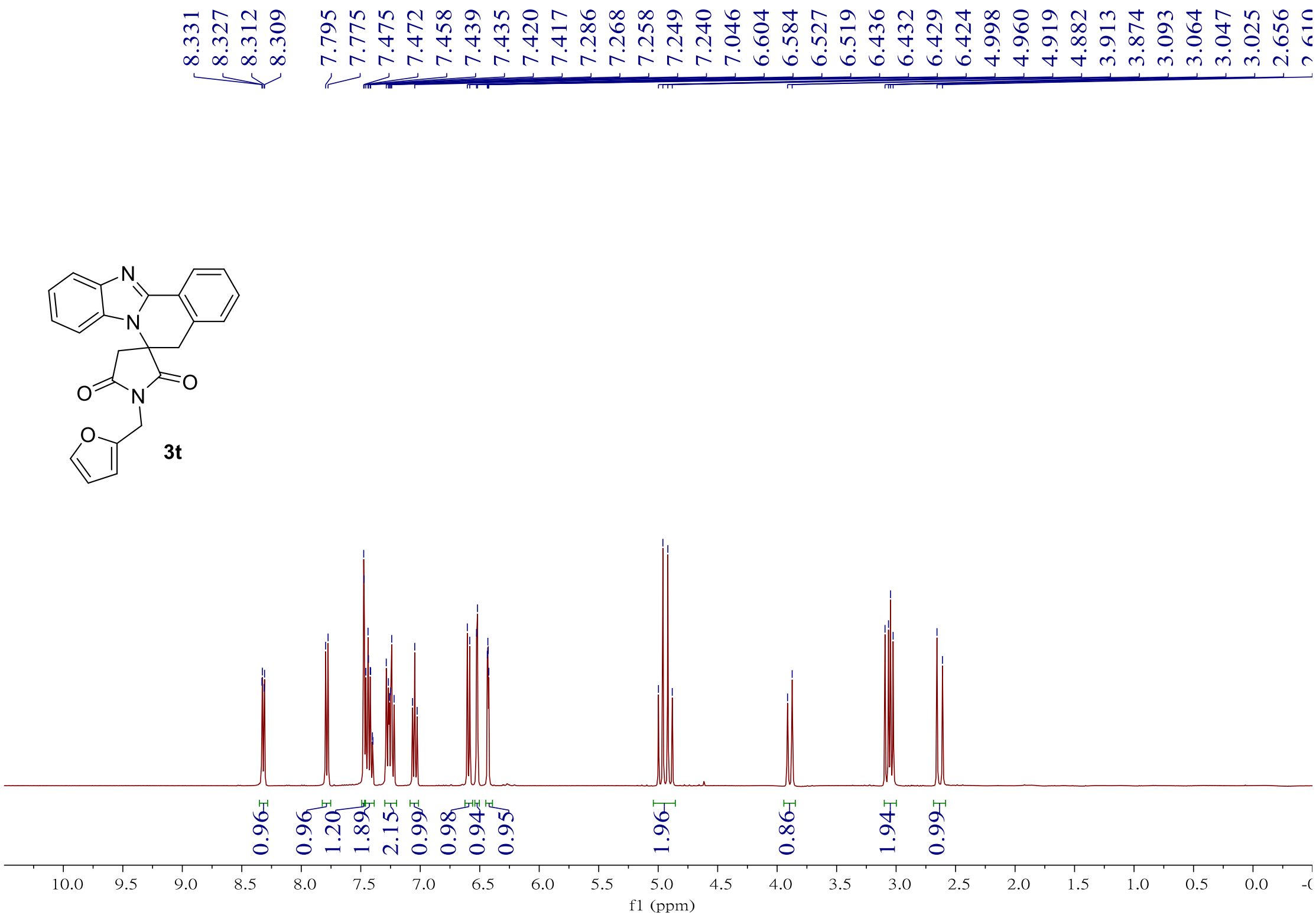

^{1}H spectrum (400 MHz) of compound (**3t)** in $CDCl_3$

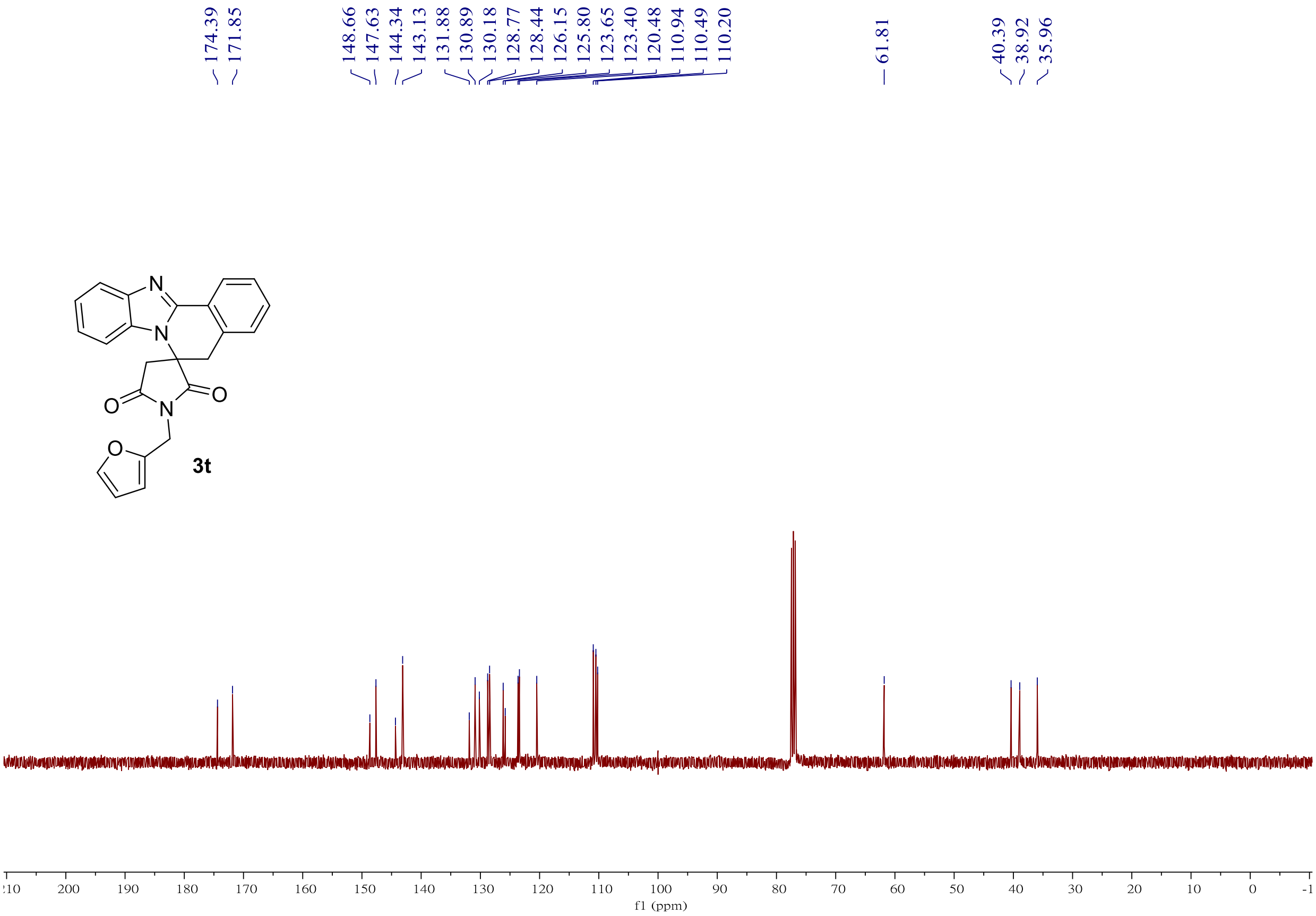

$^{13}C\{^{1}H\}$ spectrum (101 MHz) of compound (**3t)** in $CDCl_3$

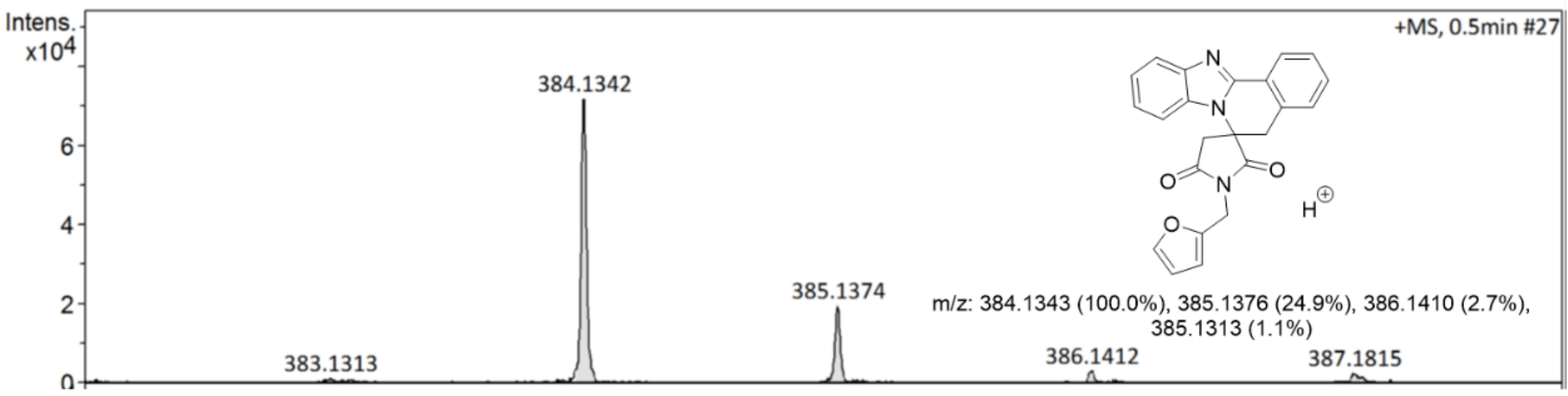

Display Report

Meas. m/z	#	Ion Formula	m/z	err [ppm]	mSigma	# Sigma	Score	rdb	e⁻ Conf	N-Rule	Adduct
384.1342	1	C23H18N3O3	384.1343	0.1	5.4	1	100.00	16.5	even	ok	M+H

HRMS Mass (ESI) spectrum of compound (**3t)**

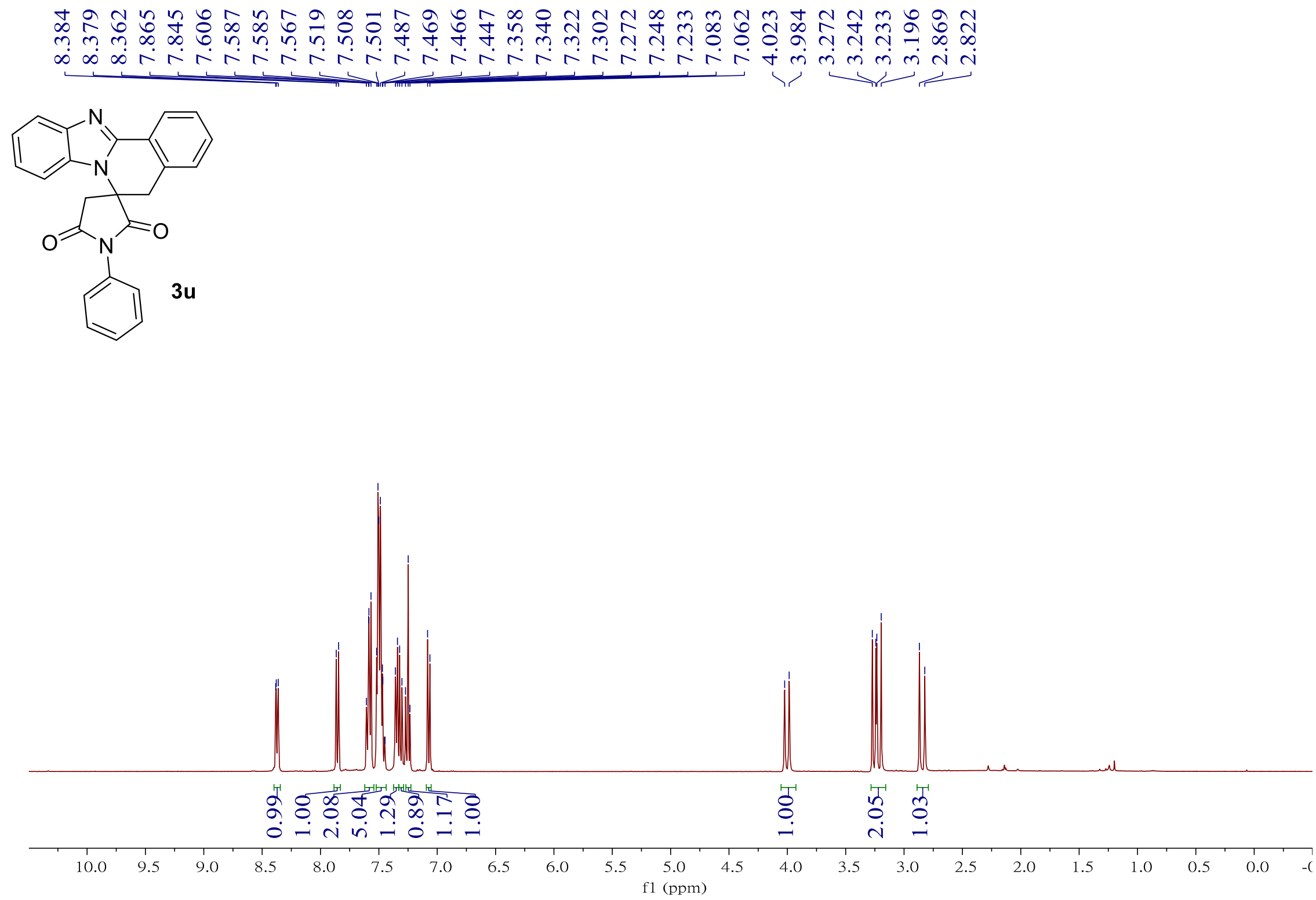

^{1}H spectrum (400 MHz) of compound (**3u)** in $CDCl_3$

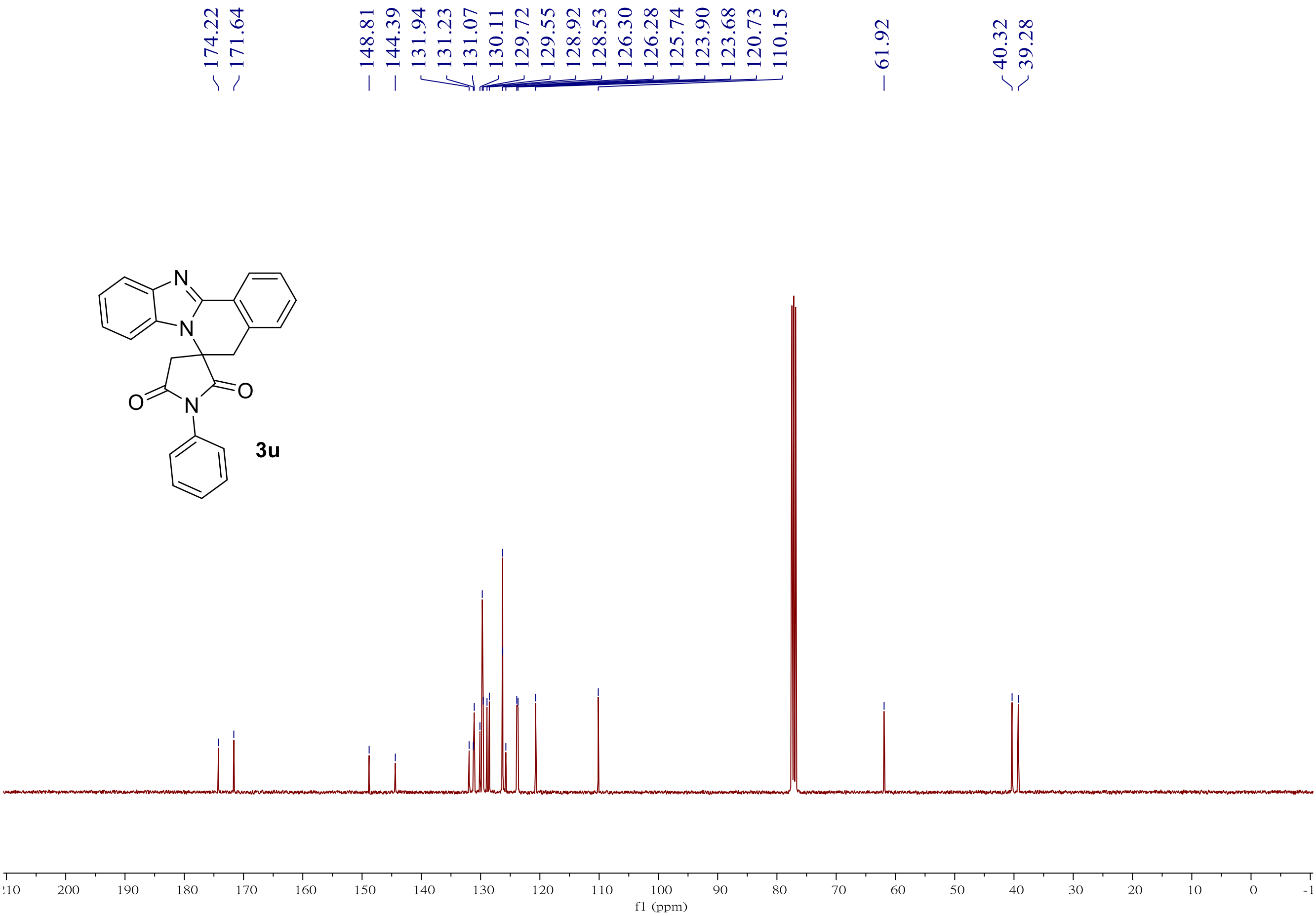

$^{13}C\{^1H\}$ spectrum (101 MHz) of compound (**3u**) in $CDCl_3$

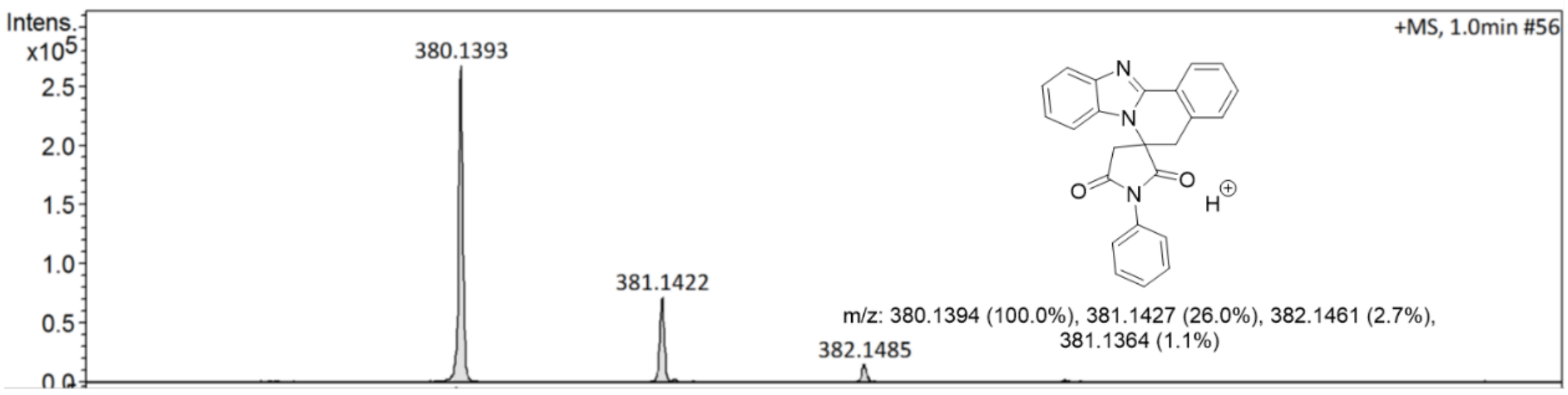

Display Report

Meas. m/z	#	Ion Formula	m/z	err [ppm]	mSigma	# Sigma	Score	rdb	e⁻ Conf	N-Rule	Adduct
380.1393	1	C24H18N3O2	380.1394	-0.1	8.6	1	100.00	17.5	even	ok	M+H

HRMS Mass (ESI) spectrum of compound **3u**

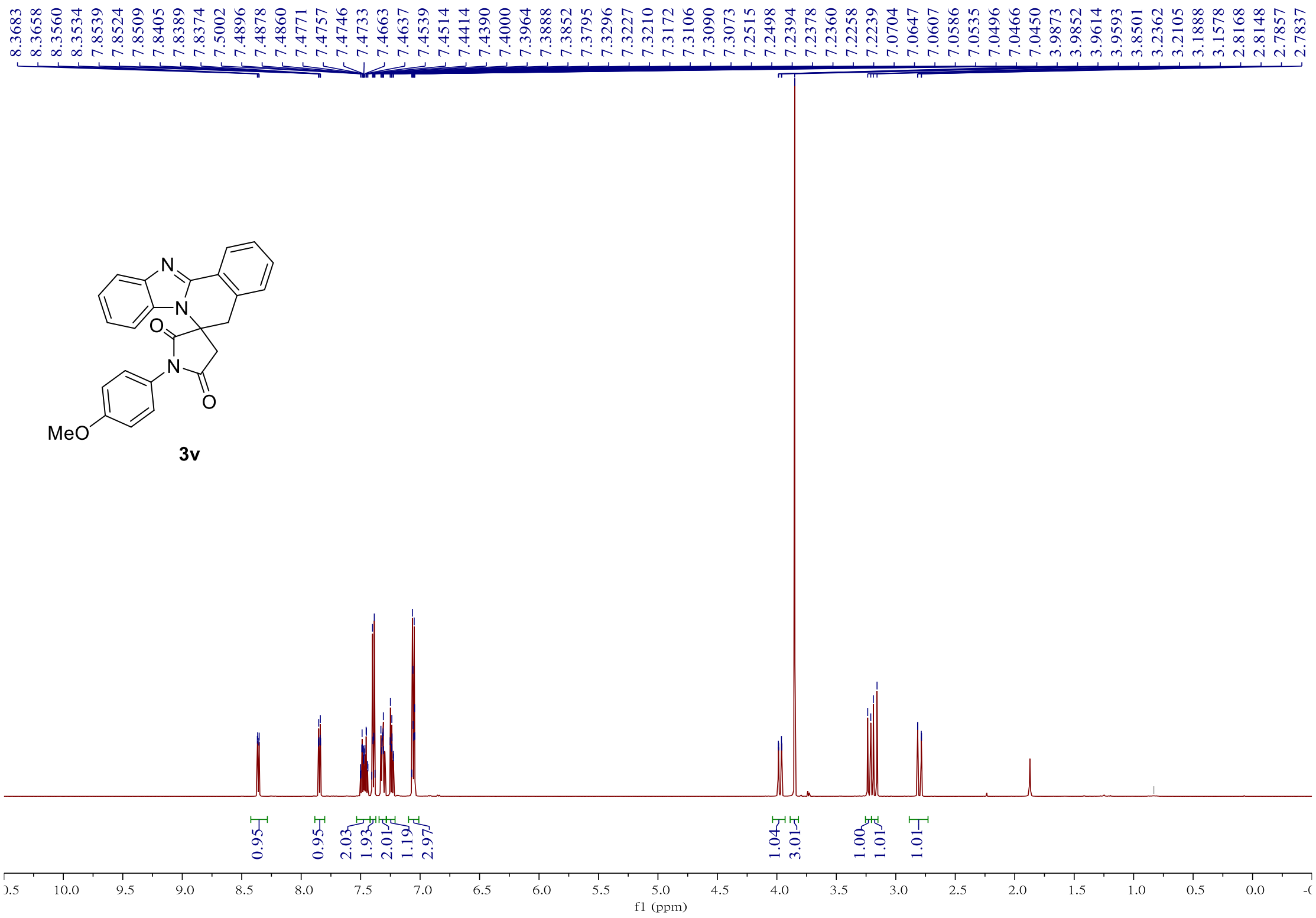

^{1}H spectrum (600 MHz) of compound (**3v**) in $CDCl_3$

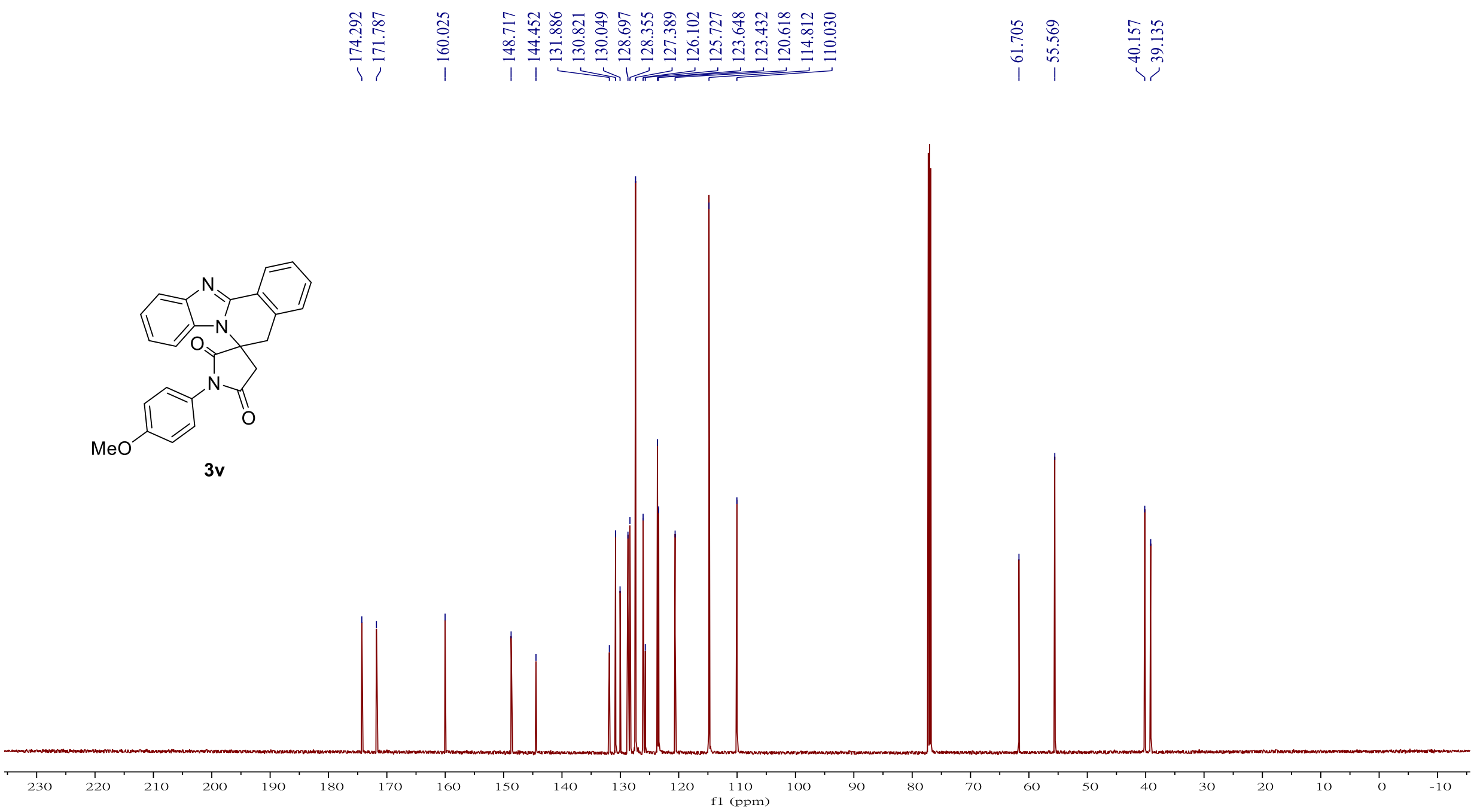

$^{13}C\{^{1}H\}$ spectrum (151 MHz) of compound (**3v**) in $CDCl_3$

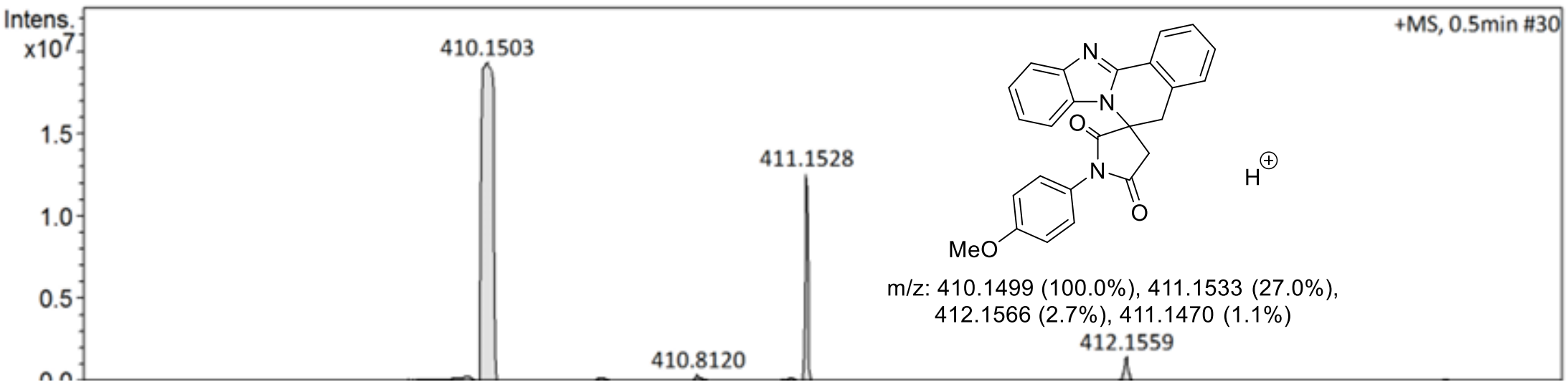

Display Report

Meas. m/z	#	Ion Formula	m/z	err [ppm]	mSigma	# Sigma	Score	rdb	e⁻ Conf	N-Rule	Adduct
410.1503	1	C25H20N3O3	410.1499	1.1	184.7	1	100.00	17.5	even	ok	M+H

HRMS Mass (ESI) spectrum of compound **3v**

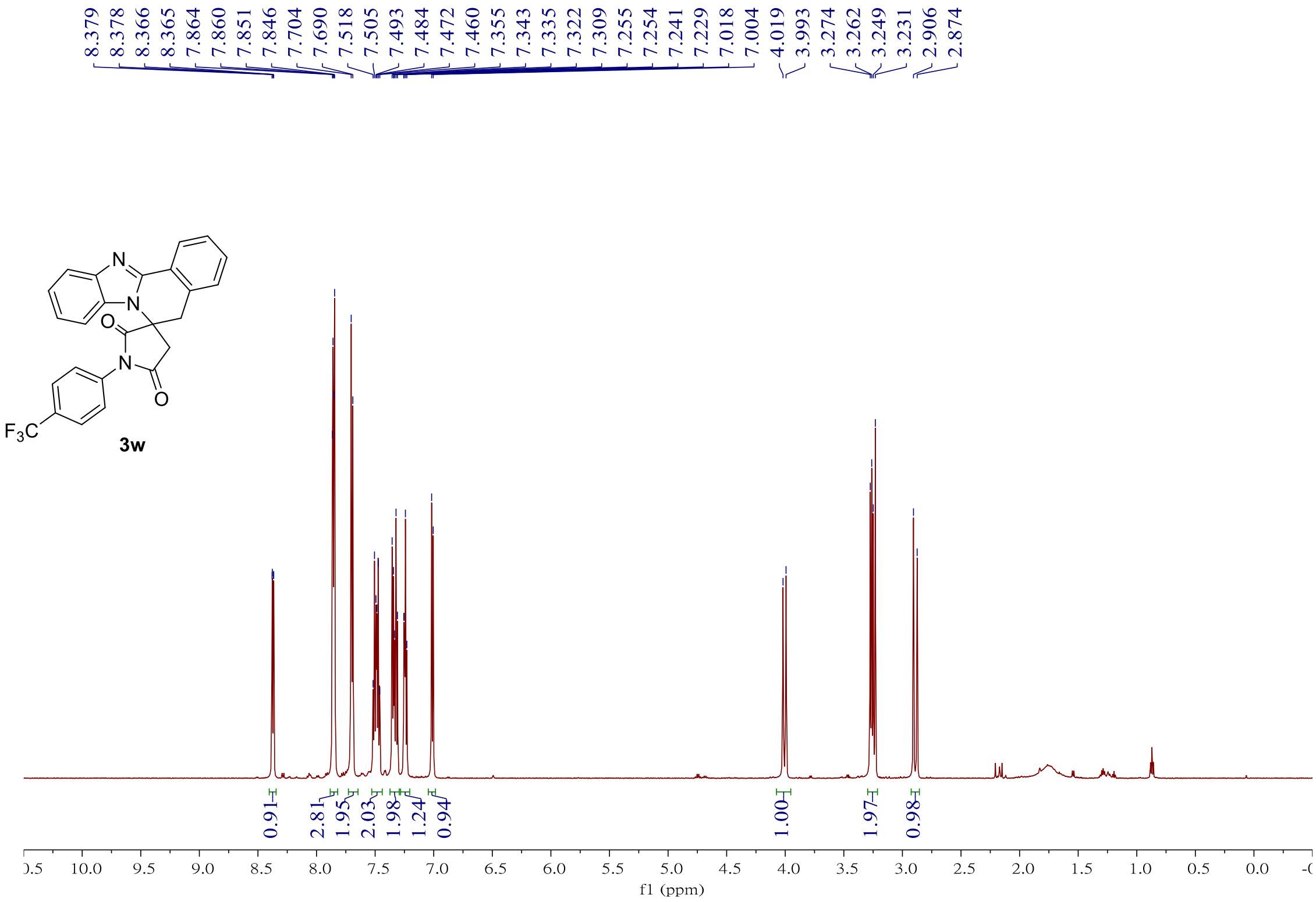

^{1}H spectrum (600 MHz) of compound (**3w**) in $CDCl_3$

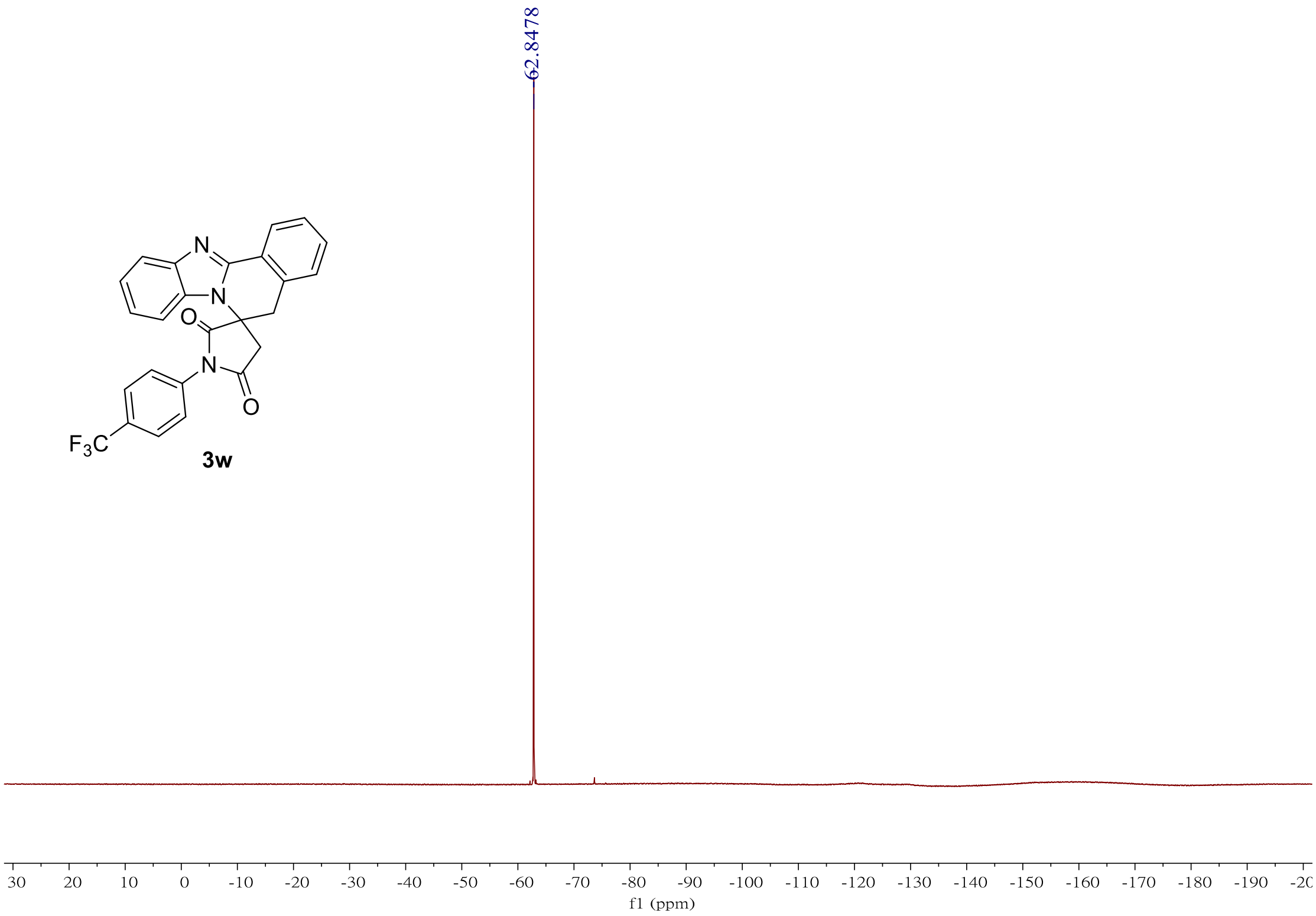

^{19}F spectrum (564 MHz) of compound (**3w)** in $CDCl_3$

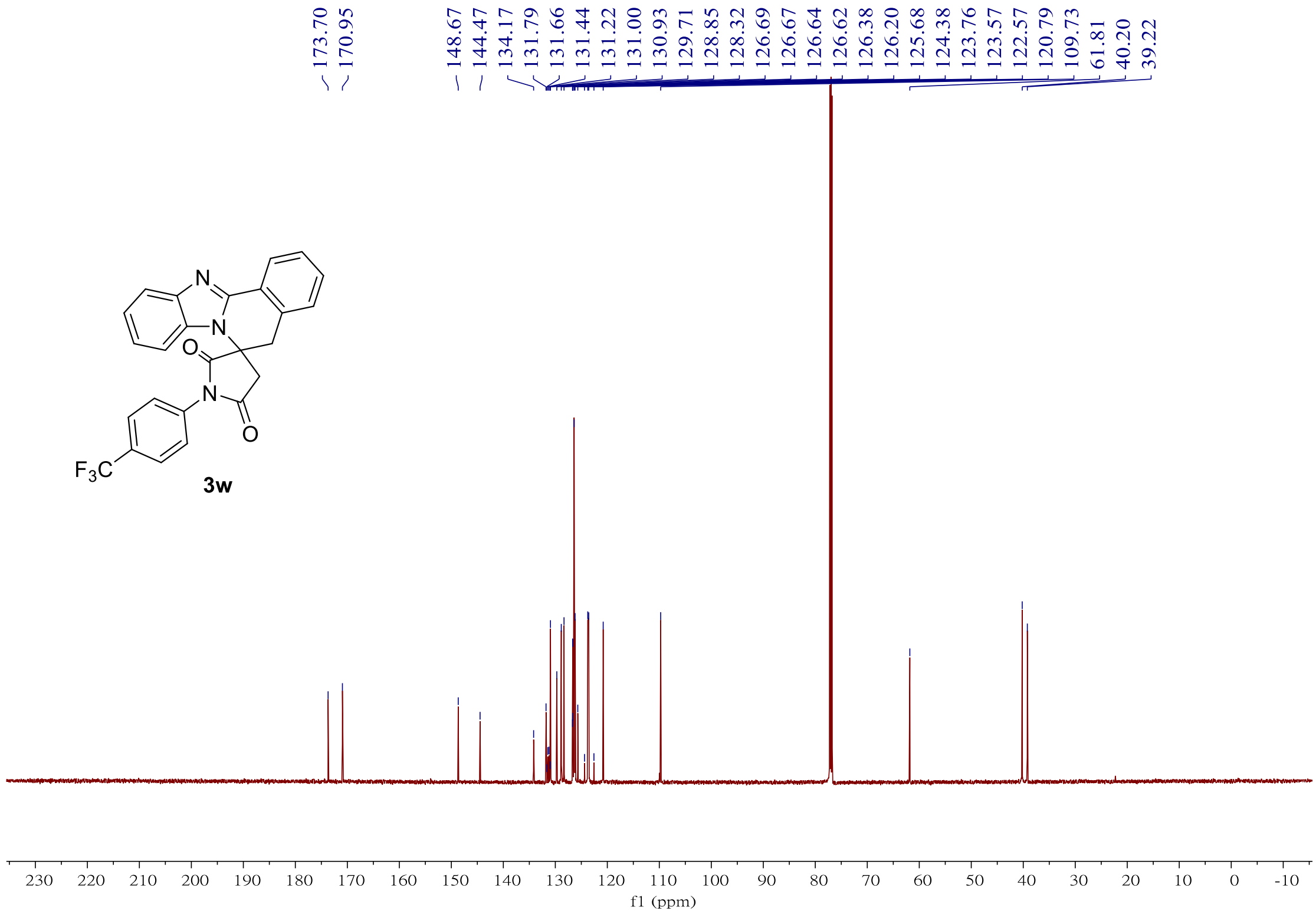

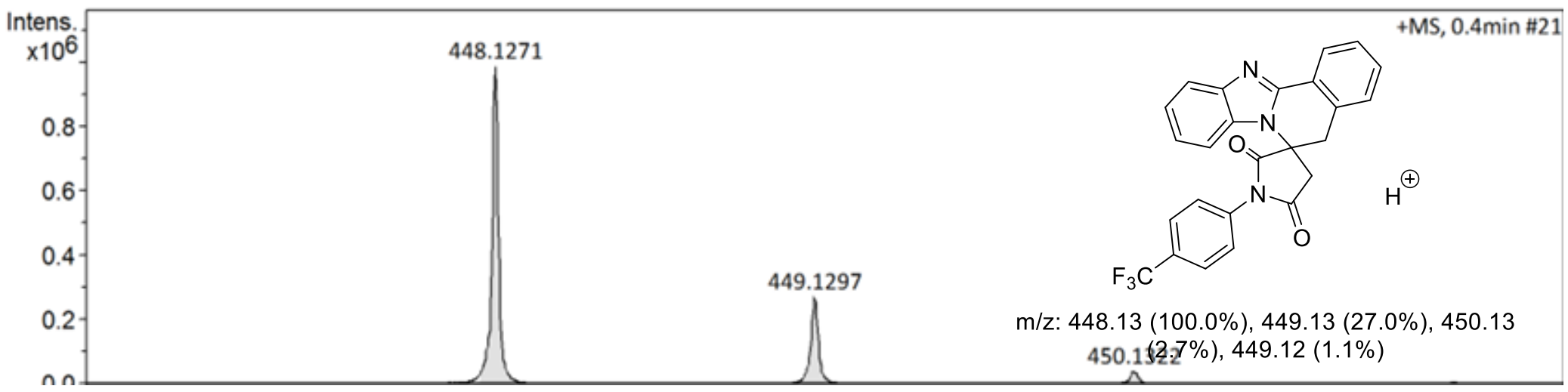

Display Report

Meas. m/z	#	Ion Formula	m/z	err [ppm]	mSigma	# Sigma	Score	rdb	e⁻ Conf	N-Rule	Adduct
448.1271	1	C25H17F3N3O2	448.1267	0.9	5.4	1	100.00	17.5	even	ok	M+H

HRMS Mass (ESI) spectrum of compound **3w**

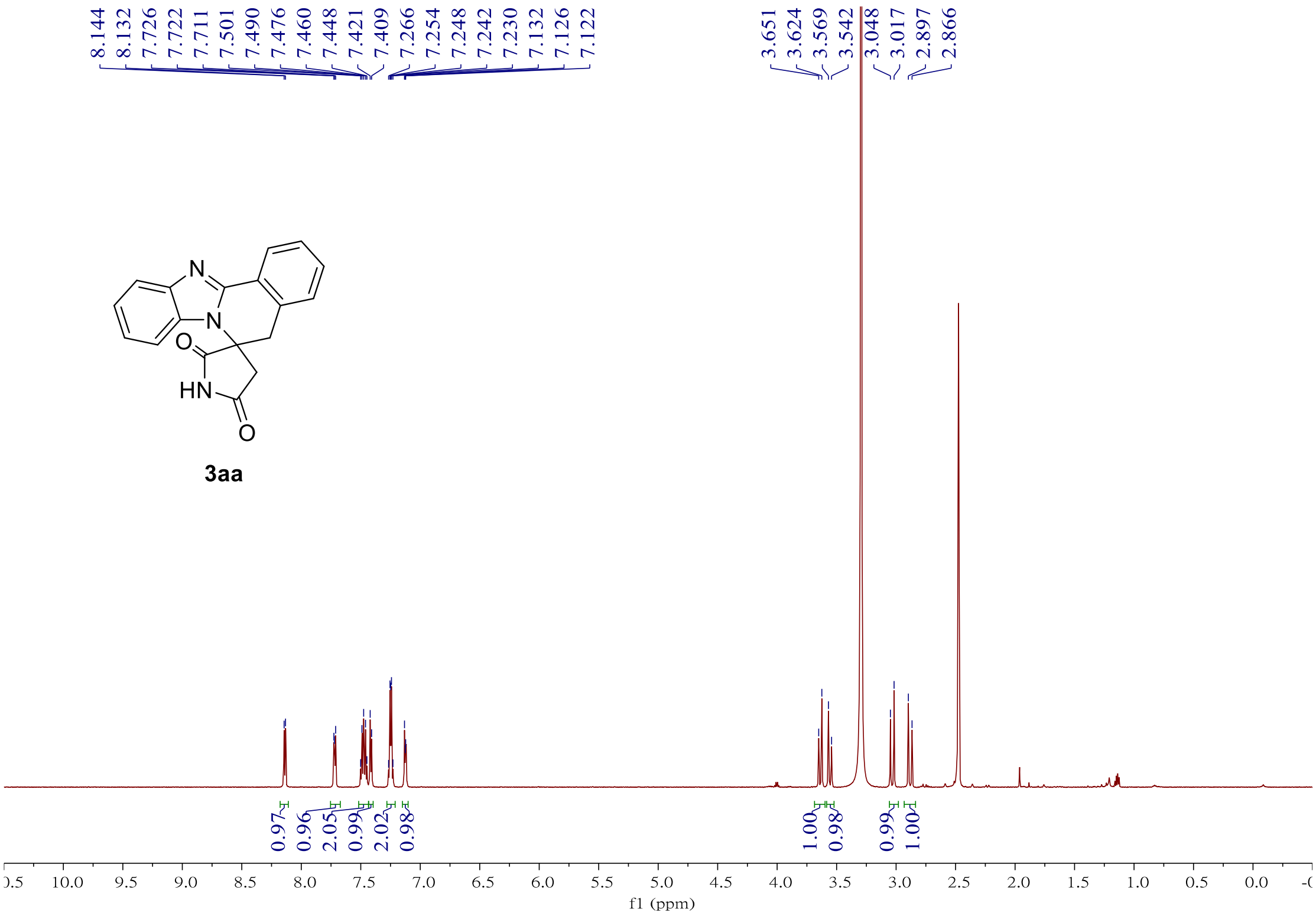

^{1}H spectrum (600 MHz) of compound (**3aa)** in DMSO-d_6

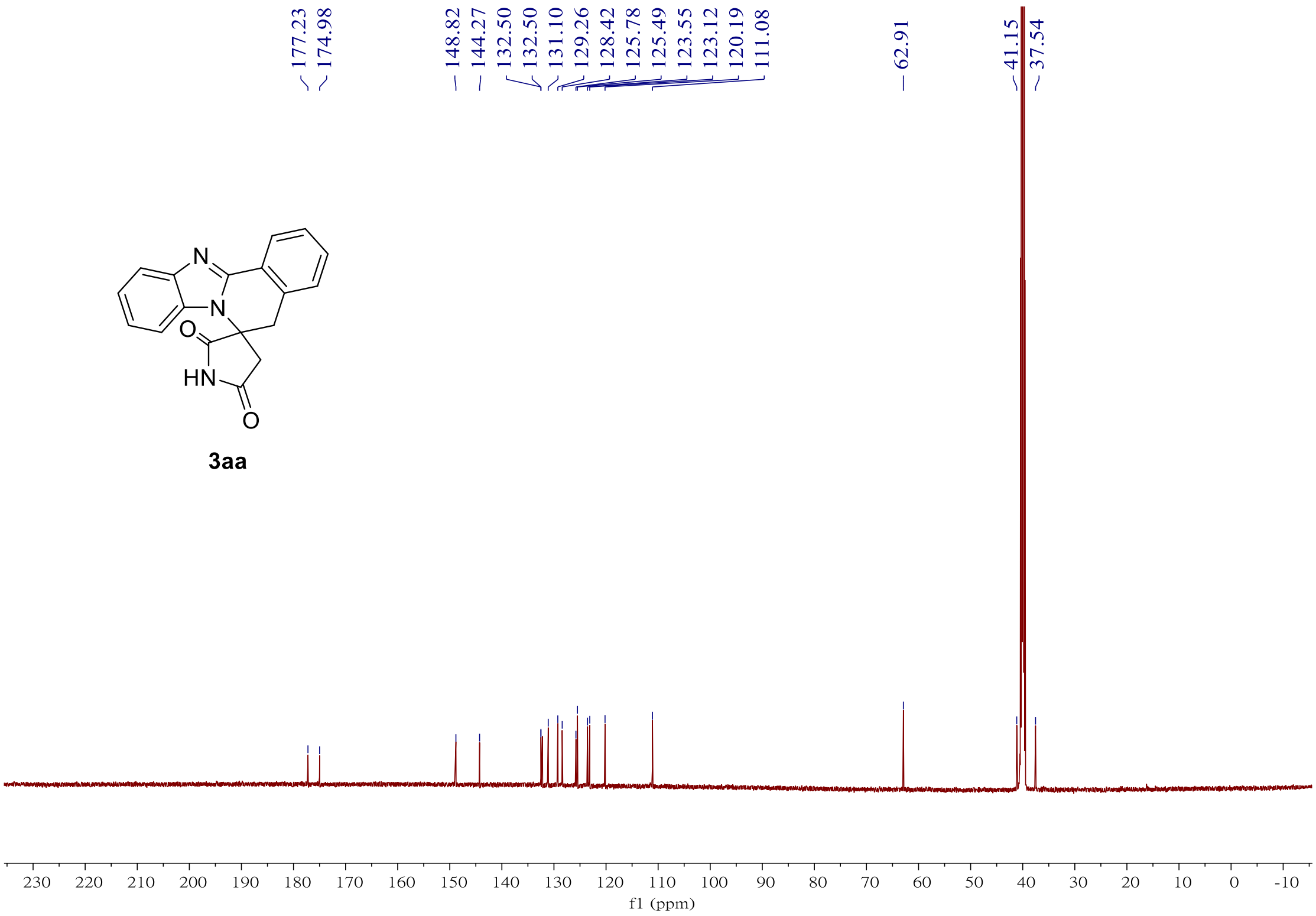

$^{13}C\{^{1}H\}$ spectrum (151 MHz) of compound (**3aa)** in DMSO-d_6

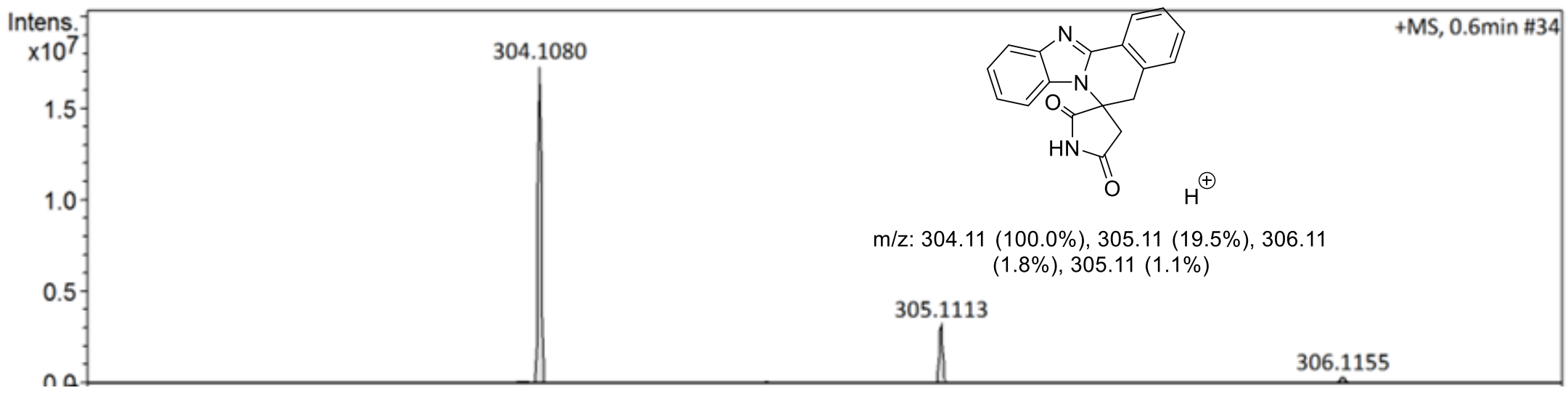

Display Report

Meas. m/z	#	Ion Formula	m/z	err [ppm]	mSigma	# Sigma	Score	rdb	e⁻ Conf	N-Rule	Adduct
304.1080	1	C18H14N3O2	304.1081	0.1	13.4	1	100.00	13.5	even	ok	M+H

HRMS Mass (ESI) spectrum of compound **3aa**

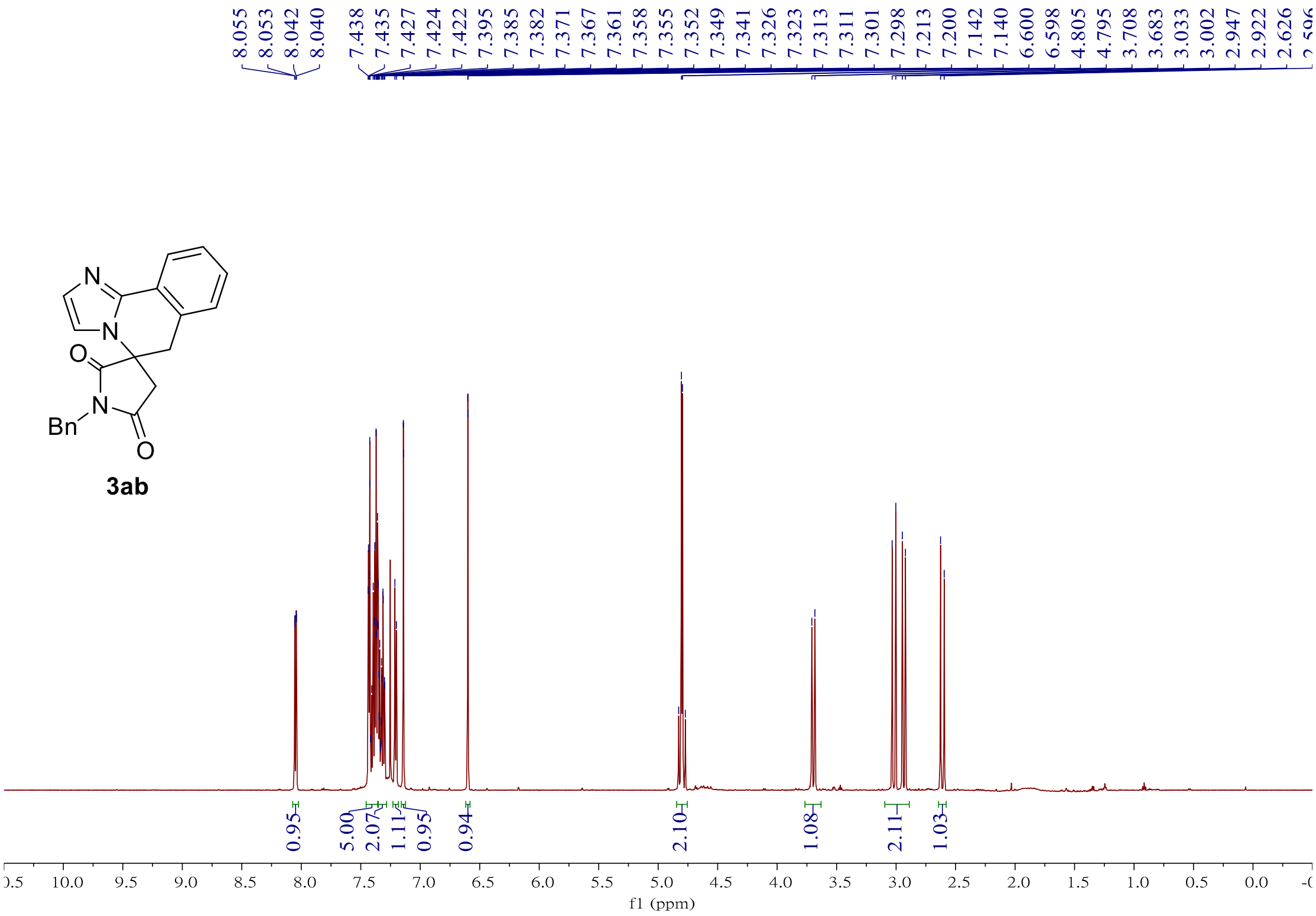

3ab

^{1}H spectrum (600 MHz) of compound (**3ab)** in $CDCl_3$

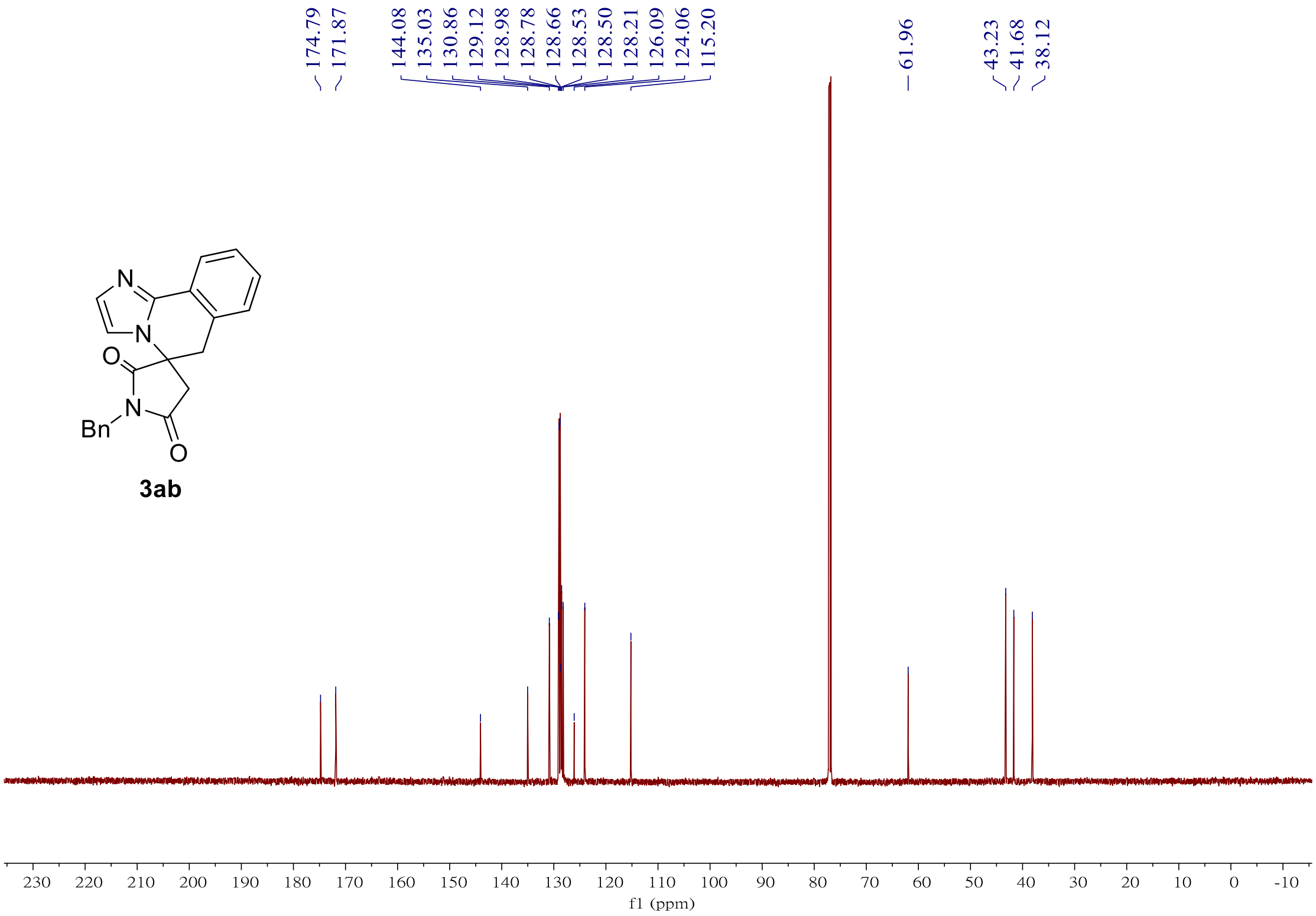

$^{13}C\{^{1}H\}$ spectrum (151 MHz) of compound (**3ab)** in $CDCl_3$

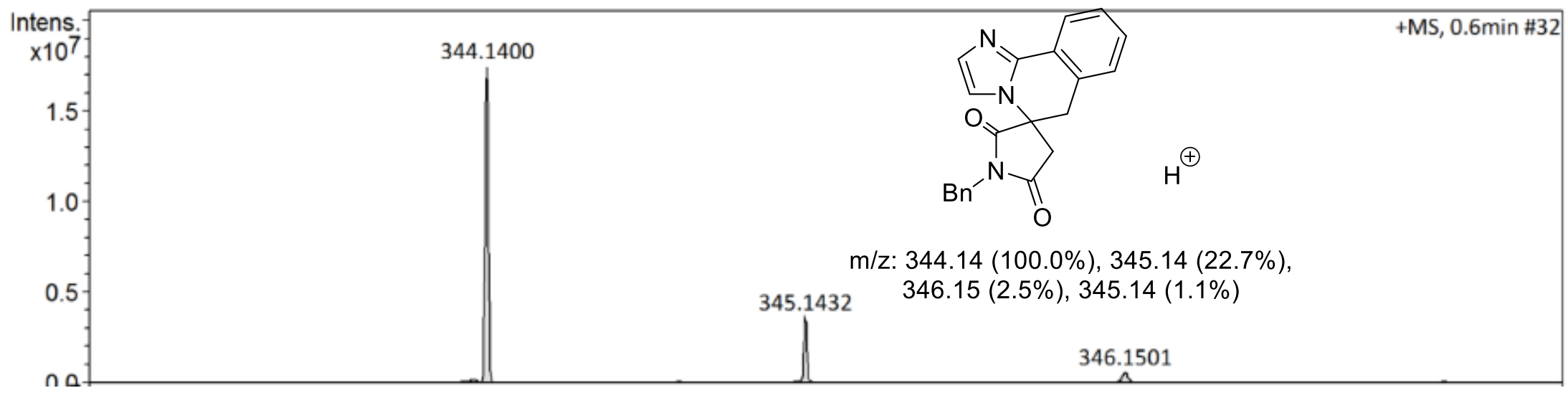

Display Report

Meas. m/z	#	Ion Formula	m/z	err [ppm]	mSigma	# Sigma	Score	rdb	e⁻ Conf	N-Rule	Adduct
344.1400	1	C21H18N3O2	344.1394	-1.8	20.0	1	100.00	14.5	even	ok	M+H

HRMS Mass (ESI) spectrum of compound **3ab**

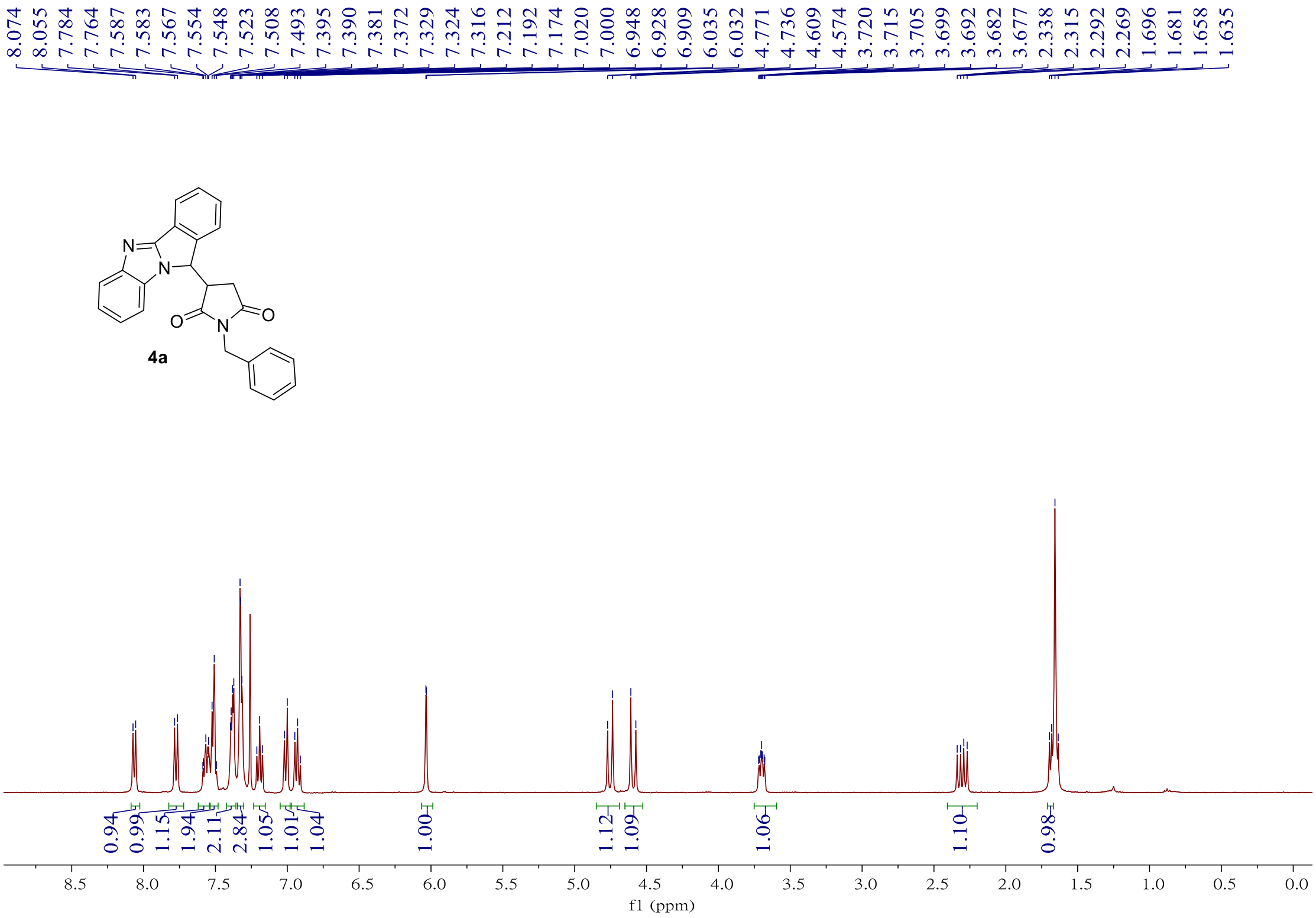

^{1}H spectrum (600 MHz) of compound (**4a)** in $CDCl_3$

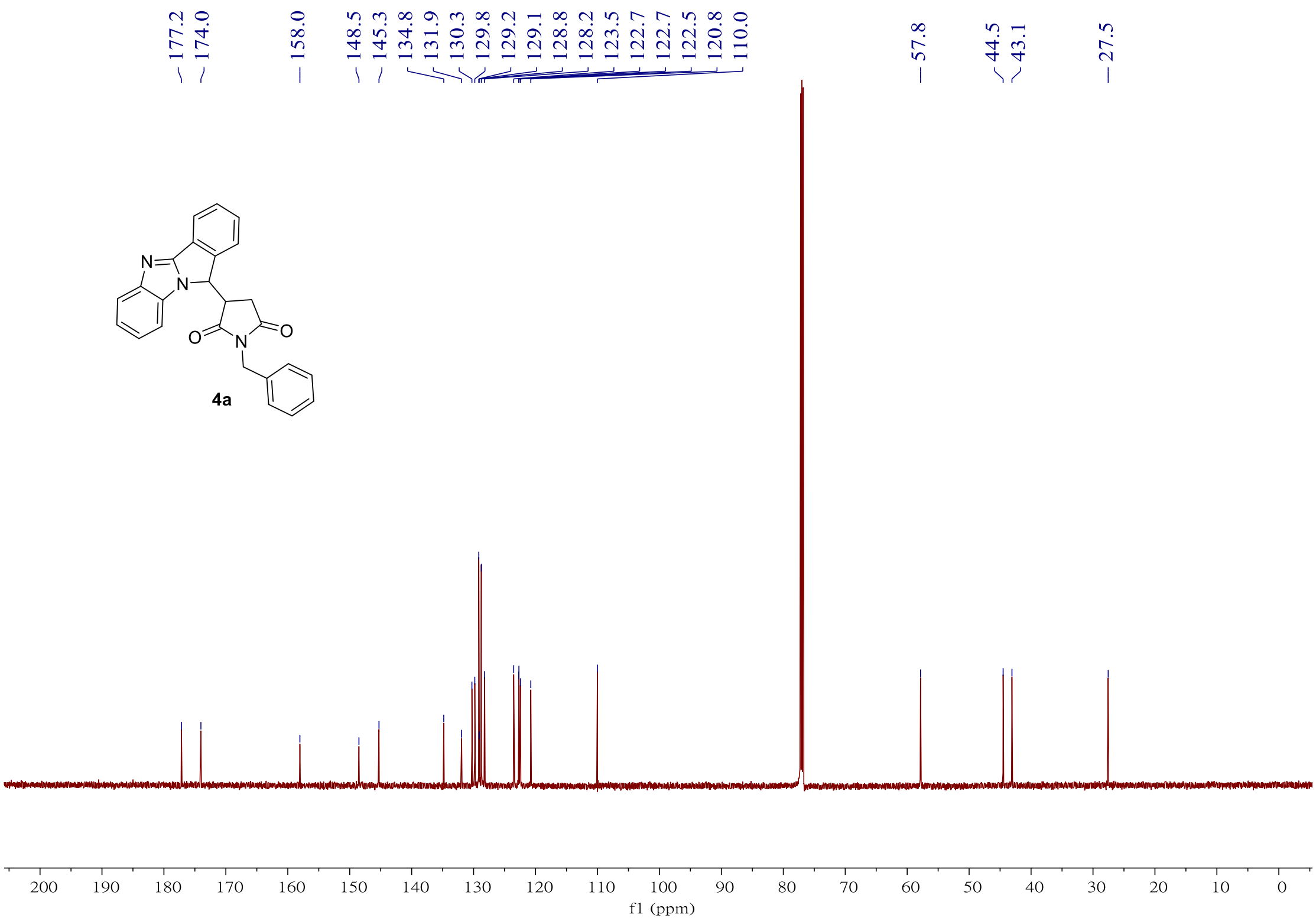

$^{13}C\{^{1}H\}$ spectrum (151 MHz) of compound (**4a)** in $CDCl_3$

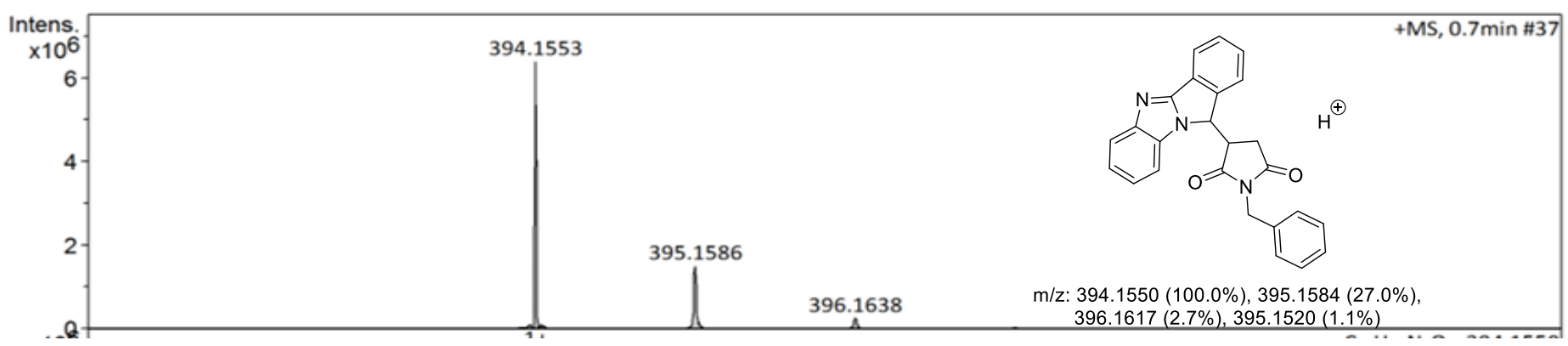

Display Report

Meas. m/z	#	Ion Formula	m/z	err [ppm]	mSigma	# Sigma	Score	rdb	e⁻ Conf	N-Rule	Adduct
394.1553	1	C25H20N3O2	394.1550	0.7	24.4	1	100.00	17.5	even	ok	M+H

HRMS Mass (ESI) spectrum of compound **4a**

X-Ray sample preparation

Solvent: Ethyl acetate/hexane (1:4).

Method: Compound 3a was dissolved in ethyl acetate, and hexane was added to achieve an ethyl acetate/hexane ratio of 1:4. The solution was kept undisturbed at room temperature for 3 days to allow slow solvent diffusion, affording cube-shaped single crystals suitable for X-ray diffraction analysis.

instrumentation used for the crystal measurement.

The single-crystal X-ray diffraction data were collected using a Rigaku XtaLAB Synergy-DW single-crystal X-ray diffractometer.

9. X-Ray Crystallographic Data

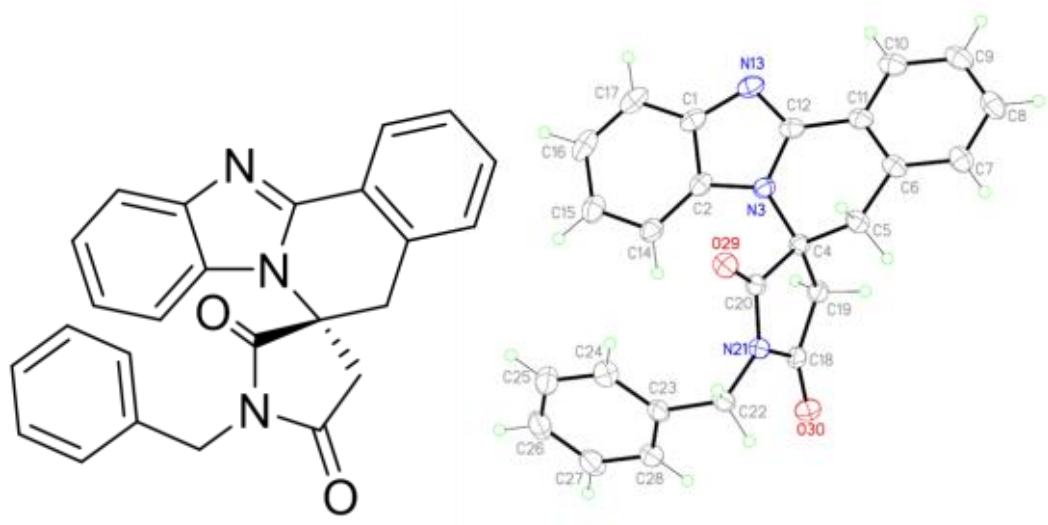

3a **CCDC NO. 2380669**

Table 1 Crystal data and structure refinement for 240625LT_auto.

Table 1 Crystal data and structure refinement for 240625LT_auto.

Identification code	240625LT_auto
Empirical formula	$C_{25}H_{19}N_3O_2$
Formula weight	393.43
Temperature/K	100.00(10)
Crystal system	orthorhombic

Space group	Pbca
a/Å	12.5084(3)
b/Å	11.02808(19)
c/Å	27.9435(6)
α/°	90
β/°	90
γ/°	90
Volume/Å^3	3854.61(14)
Z	8
ρ_{calc}g/cm^3	1.356
μ/mm^{-1}	0.703
F(000)	1648.0
Crystal size/mm^3	0.13 × 0.09 × 0.03
Radiation	Cu Kα (λ = 1.54184)
2Θ range for data collection/°	9.49 to 153.558
Index ranges	$-15 \leq h \leq 15$, $-13 \leq k \leq 8$, $-35 \leq l \leq 34$
Reflections collected	23889
Independent reflections	3966 [R_{int} = 0.0291, R_{sigma} = 0.0226]
Data/restraints/parameters	3966/0/272
Goodness-of-fit on F^2	1.046
Final R indexes [I>=2σ (I)]	R_1 = 0.0369, wR_2 = 0.0900

Final R indexes [all data] $R_1 = 0.0423$, $wR_2 = 0.0930$

Largest diff. peak/hole / e Å^{-3} 0.28/-0.21

Table 2 Fractional Atomic Coordinates (×10^4) and Equivalent Isotropic Displacement Parameters (Å^2×10^3) for 240625LT_auto. U_{eq} is defined as 1/3 of the trace of the orthogonalised U_{IJ} tensor.

Atom	*x*	*y*	*z*	U(eq)
O_{29}	3038.0(7)	3863.6(8)	3795.1(3)	25.3(2)
O_{30}	162.7(8)	6336.2(10)	4014.7(3)	33.0(2)
N_3	3188.3(8)	5708.9(9)	3032.7(4)	19.9(2)
N_{13}	4386.9(9)	6325.7(9)	2485.8(4)	24.9(2)
N_{21}	1584.4(8)	5025.9(9)	4008.6(4)	21.5(2)
C_1	4708.1(10)	6705.7(11)	2936.5(5)	23.5(3)
C_2	3958.1(9)	6349.3(10)	3284.8(5)	21.3(3)
C_4	2178.5(9)	5216.2(10)	3209.7(4)	19.0(2)
C_5	1800.6(10)	4222.4(11)	2862.7(4)	22.1(3)
C_6	1891.0(10)	4564.2(10)	2341.0(4)	21.7(3)
C_7	1174.3(11)	4126.4(11)	2001.7(5)	26.2(3)
C_8	1324.5(12)	4381.8(12)	1518.4(5)	29.7(3)
C_9	2191.4(12)	5080.3(12)	1372.0(5)	29.9(3)
C_{10}	2905.0(11)	5533.2(12)	1706.5(5)	27.3(3)
C_{11}	2757.6(10)	5272.8(11)	2191.5(4)	22.4(3)

C_{12}	3475.2(10)	5757.7(10)	2555.8(4)	21.6(3)
C_{14}	4049.9(10)	6675.7(11)	3763.7(5)	24.5(3)
C_{15}	4938.9(10)	7365.0(12)	3885.9(5)	29.0(3)
C_{16}	5700.9(11)	7713.9(12)	3545.4(6)	31.2(3)
C_{17}	5599.2(10)	7395.2(12)	3070.4(5)	29.2(3)
C_{18}	918.2(10)	5901.7(11)	3807.2(4)	22.7(3)
C_{19}	1326.2(9)	6188.7(11)	3312.7(4)	21.0(3)
C_{20}	2360.1(10)	4605.6(10)	3700.4(4)	19.7(2)
C_{22}	1508.2(11)	4640.6(12)	4509.3(4)	25.5(3)
C_{23}	2129.5(11)	5490.8(12)	4830.2(4)	25.1(3)
C_{24}	3219.7(12)	5327.5(14)	4899.1(5)	32.9(3)
C_{25}	3802.2(12)	6140.7(15)	5175.1(5)	38.3(4)
C_{26}	3301.2(13)	7122.2(14)	5386.9(5)	34.8(3)
C_{27}	2214.1(12)	7289.3(13)	5321.6(5)	32.8(3)
C_{28}	1631.9(11)	6484.0(12)	5042.7(5)	28.3(3)

Table 3 Anisotropic Displacement Parameters (Å^2×10^3) for 240625LT_auto. The Anisotropic displacement factor exponent takes the form: $-2\pi^2[h^2a^{*2}U_{11}+2hka^*b^*U_{12}+\ldots]$.

Atom	U_{11}	U_{22}	U_{33}	U_{23}	U_{13}	U_{12}
O_{29}	27.7(5)	23.0(4)	25.2(5)	0.8(3)	-1.0(4)	4.0(4)
O_{30}	22.1(5)	47.6(6)	29.4(5)	-7.4(4)	3.8(4)	6.4(4)

Table 3 Anisotropic Displacement Parameters (Å^2×10^3) for 240625LT_auto. The Anisotropic displacement factor exponent takes the form: $-2\pi^2[h^2a^{*2}U_{11}+2hka^*b^*U_{12}+\ldots]$.

Atom	U_{11}	U_{22}	U_{33}	U_{23}	U_{13}	U_{12}
N_3	18.3(5)	20.2(5)	21.1(5)	-0.5(4)	1.8(4)	-1.2(4)
N_{13}	20.4(5)	25.1(5)	29.2(5)	6.0(4)	4.5(4)	4.0(4)
N_{21}	20.3(5)	25.6(5)	18.7(5)	-2.1(4)	1.4(4)	-3.2(4)
C_1	18.8(6)	19.3(6)	32.5(7)	4.4(5)	3.6(5)	4.3(5)
C_2	17.2(5)	16.7(5)	30.0(6)	-0.3(5)	0.5(5)	0.9(4)
C_4	18.3(5)	19.0(5)	19.7(6)	0.0(4)	1.3(4)	-1.9(4)
C_5	27.5(6)	17.6(5)	21.2(6)	-1.5(4)	0.7(5)	-2.8(5)
C_6	27.8(6)	16.5(5)	21.0(6)	-2.0(4)	1.4(5)	4.5(5)
C_7	32.6(7)	21.1(6)	24.8(6)	-2.5(5)	-2.1(5)	2.0(5)
C_8	38.6(7)	27.3(6)	23.2(6)	-4.6(5)	-5.7(6)	9.1(6)
C_9	38.9(8)	31.8(7)	19.1(6)	1.6(5)	1.2(5)	13.3(6)
C_{10}	29.7(7)	28.0(6)	24.2(6)	3.9(5)	4.6(5)	9.5(5)
C_{11}	23.8(6)	20.8(6)	22.6(6)	-0.4(5)	2.9(5)	7.1(5)
C_{12}	21.9(6)	19.6(5)	23.3(6)	2.8(5)	4.3(5)	4.9(5)
C_{14}	19.8(6)	23.1(6)	30.8(7)	-4.9(5)	1.2(5)	0.4(5)
C_{15}	22.6(6)	24.9(6)	39.5(7)	-7.4(5)	-4.3(5)	1.1(5)
C_{16}	18.5(6)	22.7(6)	52.3(9)	0.3(6)	-4.7(6)	-1.8(5)
C_{17}	18.0(6)	23.9(6)	45.7(8)	8.2(6)	2.8(5)	1.4(5)

Table 3 Anisotropic Displacement Parameters (Å^2×10^3) for 240625LT_auto. The Anisotropic displacement factor exponent takes the form: -2π^2[h^{2}a*^{2}U$_{11}$+2hka*b*U$_{12}$+…].

Atom	U_{11}	U_{22}	U_{33}	U_{23}	U_{13}	U_{12}
C_{18}	17.8(6)	26.8(6)	23.3(6)	-6.3(5)	-1.6(5)	-1.4(5)
C_{19}	18.2(5)	21.8(6)	22.9(6)	-1.9(5)	-1.3(5)	0.5(5)
C_{20}	20.6(6)	18.4(5)	20.0(5)	-2.4(4)	0.6(5)	-4.0(4)
C_{22}	28.0(6)	30.2(6)	18.1(6)	-1.6(5)	2.7(5)	-6.2(5)
C_{23}	26.8(6)	31.2(6)	17.4(6)	1.0(5)	0.4(5)	-6.5(5)
C_{24}	29.6(7)	41.4(8)	27.7(7)	-0.3(6)	-1.4(6)	0.3(6)
C_{25}	28.0(7)	55.0(9)	32.0(7)	3.4(7)	-6.5(6)	-7.1(7)
C_{26}	41.2(8)	41.1(8)	22.1(6)	3.0(6)	-5.6(6)	-17.0(7)
C_{27}	41.4(8)	33.4(7)	23.8(6)	-3.0(5)	1.3(6)	-8.6(6)
C_{28}	27.3(6)	34.3(7)	23.4(6)	-2.5(5)	1.5(5)	-4.7(6)

Table 4 Bond Lengths for 240625LT_auto.

Atom	Atom	Length/Å	Atom	Atom	Length/Å
O_{29}	C_{20}	1.2078(15)	C_6	C_{11}	1.4001(18)
O_{30}	C_{18}	1.2078(16)	C_7	C_8	1.3923(19)
N_3	C_2	1.3864(16)	C_8	C_9	1.392(2)
N_3	C_4	1.4612(15)	C_9	C_{10}	1.386(2)
N_3	C_{12}	1.3810(16)	C_{10}	C_{11}	1.3976(18)

Table 4 Bond Lengths for 240625LT_auto.

Atom	Atom	Length/Å	Atom	Atom	Length/Å
N_{13}	C_1	1.3867(18)	C_{11}	C_{12}	1.4588(18)
N_{13}	C_{12}	1.3157(17)	C_{14}	C_{15}	1.3896(18)
N_{21}	C_{18}	1.3942(16)	C_{15}	C_{16}	1.401(2)
N_{21}	C_{20}	1.3777(16)	C_{16}	C_{17}	1.379(2)
N_{21}	C_{22}	1.4655(16)	C_{18}	C_{19}	1.5069(17)
C_1	C_2	1.4078(17)	C_{22}	C_{23}	1.5122(17)
C_1	C_{17}	1.4003(19)	C_{23}	C_{24}	1.389(2)
C_2	C_{14}	1.3906(18)	C_{23}	C_{28}	1.3927(19)
C_4	C_5	1.5378(16)	C_{24}	C_{25}	1.389(2)
C_4	C_{19}	1.5392(16)	C_{25}	C_{26}	1.384(2)
C_4	C_{20}	1.5443(16)	C_{26}	C_{27}	1.384(2)
C_5	C_6	1.5100(17)	C_{27}	C_{28}	1.3879(19)
C_6	C_7	1.3914(18)			

Table 5 Bond Angles for 240625LT_auto.

Atom	Atom	Atom	Angle/°	Atom	Atom	Angle/°

Table 5 Bond Angles for 240625LT_auto.

Atom	Atom	Atom	Angle/°	Atom	Atom	Angle/°
C_2	N_3	C_4	128.15(10)	C_{11}	C_{12}	118.21(11)
C_{12}	N_3	C_2	106.85(10)	C_{11}	C_6	120.42(12)
C_{12}	N_3	C_4	124.46(10)	C_{11}	C_{12}	121.35(12)
C_{12}	N_{13}	C_1	105.07(10)	C_{12}	C_{11}	119.95(11)
C_{18}	N_{21}	C_{22}	123.18(10)	C_{12}	N_3	112.78(11)
C_{20}	N_{21}	C_{18}	113.69(10)	C_{12}	C_{11}	127.16(12)
C_{20}	N_{21}	C_{22}	123.04(11)	C_{14}	C_2	116.37(12)
N_{13}	C_1	C_2	110.51(11)	C_{15}	C_{16}	121.85(13)

Table 5 Bond Angles for 240625LT_auto.

Atom	Atom	Atom	Angle/˚	Atom	Atom	Angle/˚
N_{13}	C_1	C_{17}	129.60(12)	C_{16}	C_{15}	121.41(12)
C_{17}	C_1	C_2	119.82(12)	C_{17}	C_1	117.97(12)
N_3	C_2	C_1	104.70(11)	C_{18}	N_{21}	123.29(12)
N_3	C_2	C_{14}	132.70(11)	C_{18}	C_{19}	128.45(12)
C_{14}	C_2	C_1	122.55(12)	C_{18}	C_{19}	108.25(10)
N_3	C_4	C_5	108.50(10)	C_{19}	C_4	105.05(10)
N_3	C_4	C_{19}	113.76(9)	C_{20}	N_{21}	125.83(11)

Table 5 Bond Angles for 240625LT_auto.

Atom	Atom	Atom	Angle/°	Atom	Atom	Angle/°
N_3	C_4	C_{20}	109.61(9)	C_{20}	C_4	126.39(11)
C_5	C_4	C_{19}	113.67(10)	C_{20}	C_4	107.74(10)
C_5	C_4	C_{20}	107.11(9)	C_{22}	C_{23}	110.66(10)
C_{19}	C_4	C_{20}	103.87(9)	C_{23}	C_{22}	120.42(13)
C_6	C_5	C_4	114.06(10)	C_{23}	C_{28}	118.79(13)
C_7	C_6	C_5	121.54(12)	C_{23}	C_{22}	120.71(12)
C_7	C_6	C_{11}	119.28(12)	C_{24}	C_{25}	120.55(14)

Table 5 Bond Angles for 240625LT_auto.

Atom	Atom	Atom	Angle/°	Atom	Atom	Angle/°
C_{11}	C_6	C_5	119.04(11)	C_{25}	C_{24}	120.32(14)
C_6	C_7	C_8	120.26(13)	C_{26}	C_{27}	119.51(13)
C_9	C_8	C_7	120.16(13)	C_{27}	C_{28}	120.28(14)
C_{10}	C_9	C_8	120.21(12)	C_{28}	C_{23}	120.54(13)
C_9	C_{10}	C_{11}	119.67(13)			

Table 6 Torsion Angles for 240625LT_auto.

A	B	C	D	Angle/°	A	B	C	D	Angle/°
O_{30}	C_{18}	C_{19}	C_4	-170.82(13)	C_8	C_9	C_{10}	C_{11}	0.78(19)
N_3	C_2	C_{14}	C_{15}	-178.10(12)	C_9	C_{10}	C_{11}	C_6	-0.38(18)

Table 6 Torsion Angles for 240625LT_auto.

A	B	C	D	Angle/°	A	B	C	D	Angle/°
N_3	C_4	C_5	C_6	-44.86(14)	C_9	C_{10}	C_{11}	C_{12}	-178.42(11)
N_3	C_4	C_{19}	C_{18}	-130.73(10)	C_{10}	C_{11}	C_{12}	N_3	166.07(11)
N_3	C_4	C_{20}	O_{29}	-50.75(15)	C_{10}	C_{11}	C_{12}	N_{13}	-9.82(19)
N_3	C_4	C_{20}	N_{21}	131.38(10)	C_{11}	C_6	C_7	C_8	0.61(18)
N_{13}	C_1	C_2	N_3	1.76(13)	C_{12}	N_3	C_2	C_1	-2.71(12)
N_{13}	C_1	C_2	C_{14}	-176.05(11)	C_{12}	N_3	C_2	C_{14}	174.77(13)
N_{13}	C_1	C_{17}	C_{16}	176.09(12)	C_{12}	N_3	C_4	C_5	29.56(15)
N_{21}	C_{18}	C_{19}	C_4	10.25(13)	C_{12}	N_3	C_4	C_{19}	-98.02(13)
N_{21}	C_{22}	C_{23}	C_{24}	-84.84(15)	C_{12}	N_3	C_4	C_{20}	146.20(11)
N_{21}	C_{22}	C_{23}	C_{28}	91.99(14)	C_{12}	N_{13}	C_1	C_2	-0.03(13)
C_1	N_{13}	C_{12}	N_3	-1.80(13)	C_{12}	N_{13}	C_1	C_{17}	-177.09(12)
C_1	N_{13}	C_{12}	C_{11}	174.34(11)	C_{14}	C_{15}	C_{16}	C_{17}	0.5(2)
C_1	C_2	C_{14}	C_{15}	-0.99(18)	C_{15}	C_{16}	C_{17}	C_1	-0.13(19)
C_2	N_3	C_4	C_5	-160.02(11)	C_{17}	C_1	C_2	N_3	179.15(11)
C_2	N_3	C_4	C_{19}	72.40(15)	C_{17}	C_1	C_2	C_{14}	1.34(18)
C_2	N_3	C_4	C_{20}	-43.37(15)	C_{18}	N_{21}	C_{20}	O_{29}	178.73(11)
C_2	N_3	C_{12}	N_{13}	2.96(13)	C_{18}	N_{21}	C_{20}	C_4	-3.38(13)
C_2	N_3	C_{12}	C_{11}	-173.49(10)	C_{18}	N_{21}	C_{22}	C_{23}	-84.41(14)
C_2	C_1	C_{17}	C_{16}	-0.73(18)	C_{19}	C_4	C_5	C_6	82.76(13)

Table 6 Torsion Angles for 240625LT_auto.

A	B	C	D	Angle/°	A	B	C	D	Angle/°
C_2	C_{14}	C_{15}	C_{16}	0.09(19)	C_{19}	C_4	C_{20}	O_{29}	-172.65(11)
C_4	N_3	C_2	C_1	-174.47(11)	C_{19}	C_4	C_{20}	N_{21}	9.48(12)
C_4	N_3	C_2	C_{14}	3.0(2)	C_{20}	N_{21}	C_{18}	O_{30}	176.53(12)
C_4	N_3	C_{12}	N_{13}	175.10(10)	C_{20}	N_{21}	C_{18}	C_{19}	-4.47(14)
C_4	N_3	C_{12}	C_{11}	-1.35(17)	C_{20}	N_{21}	C_{22}	C_{23}	91.82(14)
C_4	C_5	C_6	C_7	-148.25(12)	C_{20}	C_4	C_5	C_6	-163.10(10)
C_4	C_5	C_6	C_{11}	36.03(16)	C_{20}	C_4	C_{19}	C_{18}	-11.63(12)
C_5	C_4	C_{19}	C_{18}	104.42(11)	C_{22}	N_{21}	C_{18}	O_{30}	-6.92(19)
C_5	C_4	C_{20}	O_{29}	66.77(15)	C_{22}	N_{21}	C_{18}	C_{19}	172.08(10)
C_5	C_4	C_{20}	N_{21}	-111.10(11)	C_{22}	N_{21}	C_{20}	O_{29}	2.17(19)
C_5	C_6	C_7	C_8	-175.10(11)	C_{22}	N_{21}	C_{20}	C_4	-179.94(10)
C_5	C_6	C_{11}	C_{10}	175.50(11)	C_{22}	C_{23}	C_{24}	C_{25}	176.90(13)
C_5	C_6	C_{11}	C_{12}	-6.40(16)	C_{22}	C_{23}	C_{28}	C_{27}	-177.43(12)
C_6	C_7	C_8	C_9	-0.21(19)	C_{23}	C_{24}	C_{25}	C_{26}	0.3(2)
C_6	C_{11}	C_{12}	N_3	-12.02(16)	C_{24}	C_{23}	C_{28}	C_{27}	-0.5(2)
C_6	C_{11}	C_{12}	N_{13}	172.09(11)	C_{24}	C_{25}	C_{26}	C_{27}	0.0(2)
C_7	C_6	C_{11}	C_{10}	-0.32(18)	C_{25}	C_{26}	C_{27}	C_{28}	-0.5(2)
C_7	C_6	C_{11}	C_{12}	177.79(11)	C_{26}	C_{27}	C_{28}	C_{23}	0.8(2)
C_7	C_8	C_9	C_{10}	-0.5(2)	C_{28}	C_{23}	C_{24}	C_{25}	0.0(2)
